# Glucosamine Enhancement of BDNF Expression and Animal Cognitive Function

**DOI:** 10.3390/molecules25163667

**Published:** 2020-08-12

**Authors:** Lien-Yu Chou, Yu-Ming Chao, Yen-Chun Peng, Hui-Ching Lin, Yuh-Lin Wu

**Affiliations:** 1 Department of Physiology, School of Medicine, National Yang-Ming University, Taipei 11221, Taiwan; jackychou82@yahoo.com.tw (L.-Y.C.), chao.s976827@gmail.com (Y.-M.C.); hclin7@ym.edu.tw (H.-C.L.); 2 Department of Internal Medicine, Taichung Veterans General Hospital, Taichung 40705, Taiwan; pychunppp@gmail.com

**Keywords:** glucosamine, cognition, BDNF, PKA

## Abstract

Brain-derived neurotrophic factor (BDNF) is an important factor for memory consolidation and cognitive function. Protein kinase A (PKA) signaling interacts significantly with BDNF-provoked downstream signaling. Glucosamine (GLN), a common dietary supplement, has been demonstrated to perform a variety of beneficial physiological functions. In the current study, an in vivo model of 7-week-old C57BL/6 mice receiving daily intraperitoneal injection of GLN (0, 3, 10 and 30 mg/animal) was subjected to the novel object recognition test in order to determine cognitive performance. GLN significantly increased cognitive function. In the hippocampus GLN elevated tissue cAMP concentrations and CREB phosphorylation, and upregulated the expression of BDNF, CREB5 and the BDNF receptor TrkB, but it reduced PDE4B expression. With the in vitro model in the HT22 hippocampal cell line, GLN exposure significantly increased protein and mRNA levels of BDNF and CREB5 and induced cAMP responsive element (CRE) reporter activity; the GLN-mediated BDNF expression and CRE reporter induction were suppressed by PKA inhibitor H89. Our current findings suggest that GLN can exert a cognition-enhancing function and this may act at least in part by upregulating the BDNF levels via a cAMP/PKA/CREB-dependent pathway.

## 1. Introduction

Learning and memory are two critical functions of the brain and several different regions within the brain have been demonstrated to have involvement in the consolidation of diverse forms of learning/memory, including the cortex, striatum, amygdala and hippocampus [1]. The cortex has involvement with spatial learning; the striatum correlates with motor skills; the amygdala is related to emotional memory; and finally, the hippocampus is involved in spatial learning and working and recognition memory. Many have generally recognized the hippocampus as the most critical region [2,3].

A variety of neurotrophin (NT) polypeptides play important roles in neural activities by regulating cell proliferation, differentiation, maturation and plasticity. Among the NTs, the brain-derived neurotrophic factor (BDNF) in general performs the highest expression in the brain [4]. In the mouse model, BDNF has been shown to be required for neurogenesis in the hippocampus [5] and a declined BDNF level was noted in the ageing adults, indicating a possible connection of low BDNF to reduced memory, neurodegeneration and cognitive impairments [6]. In neurons, activation of the cAMP/PKA/cAMP-responsive element binding (CREB) protein signaling pathway can lead to the induction of an array of genes, including BDNF [7]. It has been proposed that while BDNF interacts with its cognate kinase receptor TrkB, the PKA pathway can activate and cause a positive feedback-like circle to amplify the BDNF-modulated physiological activities [8]. Phosphodiesterase (PDE) is the enzyme capable of degrading cAMP and thus it is able to attenuate the PKA signaling by reducing the availability of the intracellular cAMP. In fact, PDE4 is a cAMP-specific PDE isoform detected in various tissues, including several brain regions [9,10]. Indeed PDE4 has been regarded as a potential therapeutic target, for example for the treatment for the cognitive impairment [11]. All of these studies have pointed out that maintaining the cellular cAMP/PKA/CREB signaling by increasing the cAMP and/or by decreasing the PDE activity appears to be a potential strategy for treating a decline in cognitive functions [11].

Glucosamine (GLN) is a crucial component within glycoproteins and proteoglycans [12]. The clinical value of GLN was not established until it was first suggested for use in treating osteoarthritis [13]. Besides the glycolysis-related events, GLN and its derivatives have been demonstrated to have involvement in a variety of cellular activities in a glycolysis-independent manner [14]. GLN is involved in the *O*-linked *N*-acetylglucosaminylation (*O*-GlcNAcylation) of different proteins and this should lead to a wide range of regulation in cell physiology, such as cellular signal transduction, transcription, protein modification and more [14,15]. Importantly, most GLN administrated orally can be absorbed from the gastrointestinal system and the resultant GLN has been shown to pass the blood–brain barrier (BBB) to reach the brain [16,17], indicating that GLN can possibly reach any tissue of the body.

Previous studies have reported a list of different potential functions of GLN [15]. The involvement of *O*-GlcNAcylation in the regulation of protein homeostasis has been well-recognized; *O*-GlcNAcylation modification is highly prevalent in the mammalian brain and errors in this mechanism have been suggested to contribute to many cellular cascades in relation to neurological or neurodegenerative diseases [12,18]. Therefore, this study aimed to disclose the impact of GLN in brain cognitive performance in relation to BDNF production and PKA signaling with in vivo and in vitro approaches.

## 2. Results

### 2.1. GLN Enhancement on Animal Cognitive Function

**Figure 1:**
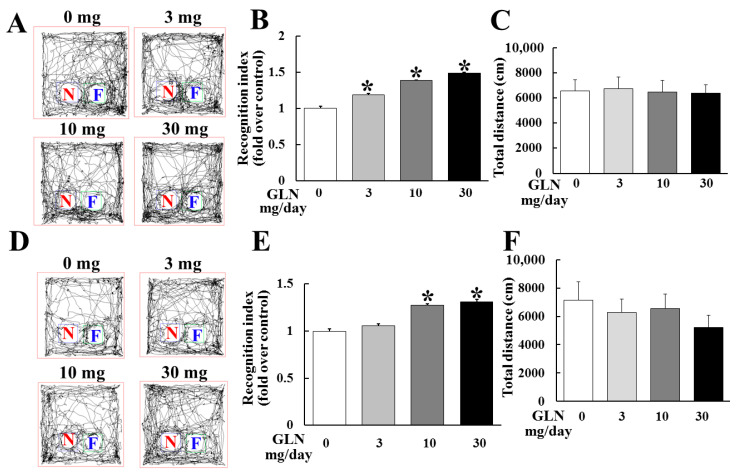
GLN enhancement of animal cognitive performance. The C57BL/6 male mice received a daily intraperitoneal (IP) GLN injection (0, 3, 10 and 30 mg/mouse) for 7 (**A**–**C**) or 14 days (**D**–**F**), followed by the novel object recognition test (NORT). A set of objects with a previously presented familiar object (**F**) and a new novel object (N) were presented to the trained animals. The moving tracks of the animals were recorded (**A**,**D**). The recognition index (**B**,**E**) and total moving distance (**C**,**F**) were determined. The results represent the means ± S.D. (*n* = 8) *, *p* < 0.05, compared with the 0 mg group.

During the 2 weeks of GLN injection (0, 3, 10 and 30 mg/mouse/day), GLN did not cause any change in body weight among different treatment groups (Supplementary Figure S1). To examine the GLN impact on cognitive function, the 7-week-old mice receiving 14 consecutive days of daily GLN intraperitoneal (IP) injection were subjected to the novel object recognition test (NORT) at day 7 and day 14 to evaluate the cognitive performance of the animals. The recorded video analyzed by the software revealed the total time spent and the tracks in relation to the familiar (F) and novel (N) objects (Figure 1A,D). The recognition index was derived from the formula of TN/TN + TF (TN: time spent exploring the novel object and TF: time spent exploring the familiar object). It appeared that the total time was not different among different treatment groups (Figure 1C,F), indicating that GLN did not affect general locomotive activity. Interestingly, there was a significant elevation of the recognition index by GLN at 3, 10 and 30 mg/mouse at day 7 (Figure 1B) and at 10 and 30 mg/mouse at day 14 (Figure 1E).

### 2.2. GLN Induction of the Genes Potentially Associated with the Brain Cognitive Functions

**Figure 2:**
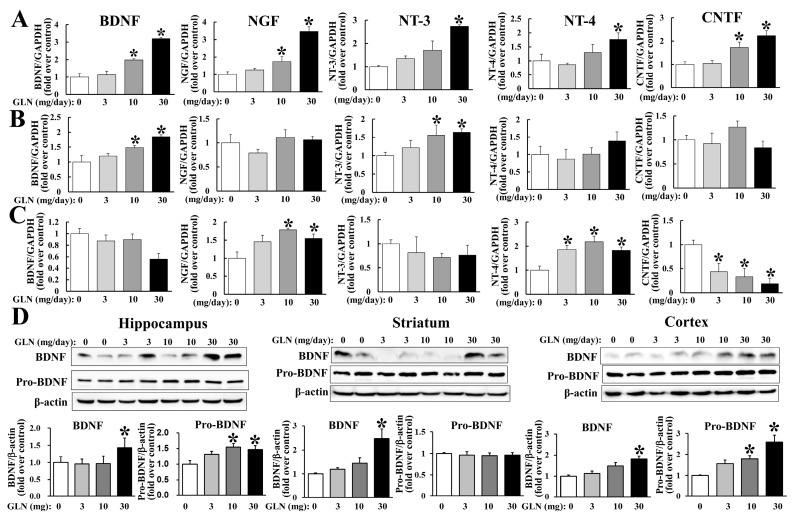
GLN impact on the expression of mRNAs encoding neurotrophins and the BDNF protein in the hippocampus, striatum and cortex. The RNAs were extracted from the hippocampus (**A**), striatum (**B**) and cortex (**C**), followed by the RT-PCR assay to detect the mRNAs of BDNF, NGF, NT-3, NT-4 and CNTF using GAPDH as an internal control. Protein samples were also prepared from the same tissues and the expression of BDNF and pro-BDNF was analyzed by Western blotting with β-actin as an internal control (**D**). The results represent the means ± S.D. (*n* = 8) *, *p* < 0.05, compared with the 0 mg group.

To examine whether GLN may regulate the genes encoding various NTs, which have been reported as important in brain functions, the hippocampus, striatum and cortex from GLN-treated animals were harvested at day 14, followed by RNA extraction, and the mRNA levels were monitored by a quantitative RT-PCR analysis. All the NTs examined, such as BDNF, NGF, NT-3, NT-4 and CNTF were all induced by GLN in hippocampus (Figure 2A); BDNF and NT-3 were induced in striatum (Figure 2B) and NGF and NT-4 were induced, but CNTF was reduced by GLN in cortex (Figure 2C). In parallel, BDNF protein expression was also examined in all three tissues. GLN was able to increase the mature form BDNF levels in all three tissues and the pro-BDNF levels in the hippocampus and cortex (Figure 1D). Notably, the BDNF cognate receptor TrkB mRNA expression in the hippocampus was also increased by GLN (Supplementary Figure S2).

### 2.3. GLN Impact on Expression of BDNF, CREB5 and PDE4B, and the PKA Signaling in the Hippocampus and HT22 Hippocampal Cells

**Figure 3:**
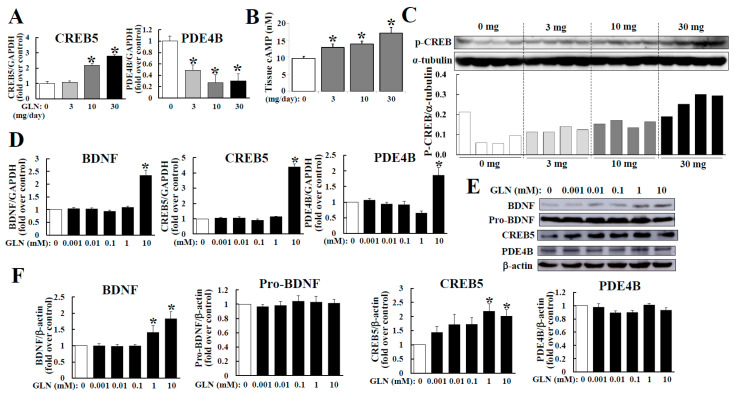
GLN regulation of CREB5 and PDE4B mRNAs, cAMP accumulation and CREB phosphorylation in the hippocampus and the expression of BDNF, CREB5 and PDE4B in the hippocampal cells. The RNA samples prepared from GLN-injected animal’s hippocampus (**A**) and from GLN-treated HT22 cells (**D**) were subjected to RT-PCR to analyze the mRNAs of BDNF, CREB5 and PDE4B. The cAMP concentration (**B**) and CREB phosphorylation (**C**) in hippocampal tissues were determined. Protein expression of BDNF, CREB5 and PDE4B in GLN-treated HT22 cells were analyzed by Western blotting (**E**,**F**). The results represent the means ± S.D. (*n* = 8). *, *p* < 0.05 compared with the 0 mg (**A**–**C**) or the 0 mM group (**D**,**F**).

As the hippocampus is the critical tissue to regulate the cognitive function and as the cAMP/PKA pathway is crucial to brain function [3,11], we therefore looked into GLN’s impact on the expression of CREB5 and PDE4B, both of which are involved in regulating cAMP/PKA signaling [9,10]. The GLN treatment (10 and 30 mg/mouse) was able to increase the CREB5 mRNA concentration, but decreased PDE4B mRNA levels in the hippocampus (Figure 3A). The cAMP concentration in the hippocampal tissue elevated after GLN treatment (Figure 3B). Meanwhile, the corresponding downstream signal activation of the cAMP/PKA in terms of CREB phosphorylation in the hippocampus seemed to be promoted by GLN (Figure 3C). In addition, we examined the GLN effect on regulation of the BDNF, CREB5 and PDE4B genes in HT22 cells. The GLN treatment (10 mM) increased the mRNA levels of BDNF, CREB5 and PDE4B (Figure 3D), while both BDNF and CREB5 protein levels were increased by GLN (1 and 10 mM), but the PDE4B protein expression remained unaffected by GLN (Figure 3E,F). To assure that the GLN treatment in HT22 cells does not affect the cellular viability, MTT and alamarBlue assays were used to confirm that GLN at all doses did not result in significant changes in cell viability (Supplementary Figure S3).

### 2.4. Delineation of the GLN Regulation of the cAMP/PKA/CREB Pathway in Relation to BDNF Production

**Figure 4:**
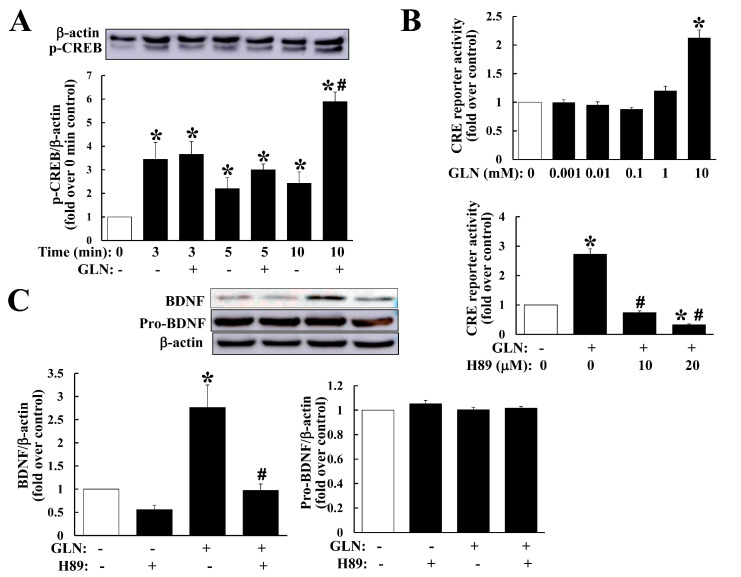
GLN impact on the cAMP/PKA signaling in relation to BDNF production in HT22 cells. HT22 cells were exposed to GLN (10 mM) for indicated times and the CREB phosphorylation manner was analyzed with Western blotting normalized by the β-actin (**A**). To examine the GLN effect on the CRE reporter activity, HT22 cells were cotransfected with a CRE reporter plasmid and a *p*-CMV-β-gal control plasmid, followed by the treatment with GLN (10 mM) alone or in combination with H89 (10 and 20 µM) for 24 h. The luciferase activity in cell lysates was analyzed and normalized against the β-gal activity within the same sample (**B**). To reveal the significance of the GLN-induced cAMP/PKA signaling in BDNF expression, HT22 cells were treated with GLN (10 mM) alone or in combination with H89 (20 µM) for 24 h and BDNF expression was determined (**C**). The results represent the means ± S.D. (*n* = 3–4). *, *p* < 0.05 compared with the 0 mM group (**A**–**C**); #, *p* < 0.05 compared with the control group within the same time point (**A**) or with the GLN alone group (**B**,**C**).

We observed GLN upregulation on BDNF in the hippocampus (Figure 2A,D) and HT22 cells (Figure 3D–F), CREB5 in the hippocampus (Figure 3A) and HT22 cells (Figure 3D–F), and CREB phosphorylation in the hippocampus (Figure 3C), and further, the downregulation on PDE4B in the hippocampus (Figure 3A). The peculiar question emerged as to how these regulatory profiles by GLN would contribute to BDNF production. In HT22 hippocampal cells, we first analyzed the GLN effect on CREB phosphorylation and GLN indeed mediated a significant induction of CREB phosphorylation (Figure 4A). GLN also induced CRE reporter activation and such an induction was suppressed by PKA inhibitor H89 (Figure 4B). More importantly, the GLN-mediated BDNF production was inhibited by H89 (Figure 4C).

## 3. Discussion

Our current study clearly demonstrated that GLN mediates an enhancement on cognitive performance in mice and an upregulation of BDNF production and the cAMP/PKA/CREB signaling in the hippocampus and hippocampal cell line. The GLN-mediated cAMP/PKA signaling appeared to connect with the induction of BDNF production.

The significance of the superfamily of NTs, including BDNF, NGF, NT-3, NT-4 and CNTF in the proliferation or differentiation of neural cells has been well-recognized [19,20]. Our findings of the upregulation of all or some of these NTs in the hippocampus, striatum or cortex and particularly the BDNF protein induced by GLN in all three tissues (Figure 2D) have strongly suggested that GLN may improve cognitive function by modulating the local production of these NTs in the brain. In fact, whenever BDNF bounds to its cognate receptor TrkB, several genes involved in neuronal survival, differentiation and synaptic plasticity would be induced [21]. The GLN upregulation of TrkB receptor expression in the hippocampus (Supplementary Figure S2) further suggests the potential role of BDNF signaling in such GLN-mediated cognition enhancement.

In the neural system, the cAMP/PKA/CREB signaling is critical in neural functions and memory formation [22]. This is consistent with our findings of increased CREB5, CREB phosphorylation and cAMP accumulation by GLN in vivo and in vitro. Previous studies have noted the importance of BDNF/TrkB and CREB signaling in promoting cognition and memory formation [23,24,25]. The functioning of PDE4, which presumably reduces the cAMP levels, is significant for attenuating the PKA signaling, and thus the inhibitors for PDE4 have been a therapeutic option to treat different CNS diseases, including memory impairment [11]. Another finding of the GLN downregulation of PD4B mRNA in the hippocampus implies that GLN may act not only by inducing CREB5, but also by suppressing PDE4B expression in the hippocampus and consequently this would lead to the upregulation of cAMP levels and CREB phosphorylation in the hippocampus (Figure 3). This appears to make GLN a potential alternative option for treating cognitive or memory diseases by improving cognitive function.

Similar to our finding of the PKA-dependence for the GLN-mediated BDNF production (Figure 4C), the cAMP/CREB-dependent induction of BDNF in developing neurons was previously reported [26]. A recent study has demonstrated a transcriptional autoregulation of BDNF in the rat hippocampus during a BDNF-induced long-term potentiation, suggesting an important intra-hippocampal transcriptional autoregulation mechanism of BDNF via the CREB activation [27]. In this study, we noted GLN’s consistent impact on upregulation of BDNF, CREB5, cAMP accumulation, CREB phosphorylation and also on the downregulation of PDE4B in the hippocampus (Figure 3), as well as the GLN-induced CRE reporter activation and CREB phosphorylation and finally a PKA-dependence of GLN-mediated BDNF production in HT22 cells (Figure 4). These highly suggest that GLN may activate the PKA pathway to first induce BDNF production, and the resultant BDNF may therefore initiate a potential autoregulation of its own expression in the hippocampus.

As a popular supplement with the capability to pass the BBB after consumption or injection, GLN is presumably able to reach the hippocampus, striatum and cortex [16,17]. Meanwhile, several different transporters for glucose or GLN have been detected in the brain [28] and the glucose transporter 2 (GLUT2) indeed performs the highest affinity for GLN and it has been detected in neurons [29,30]. For the brain, conditional GLUT2 knockout would result in a defect in neural functions and increased cell death [31]. These studies highly support the significance of GLN in brain functioning. In addition, previous studies have demonstrated that GLN administration in animals with large doses (5000–15,000 mg/kg) did not result in apparent toxicity and the median lethal dose LD50 in rats and mice was >8000 mg/kg. The subacute and chronic administration in rats, mice, rabbits and dogs receiving doses from 159 to 2700 mg/kg/day for 12–365 days did not cause significant adverse effects [32]. Thus, the daily administration of GLN of 3, 10 and 30 mg/mouse (120–1200 mg/kg) for 14 days in our current study could be regarded as relatively safe. From our current data, the question of whether a higher dose of GLN can mediate an even more potent enhancement on cognitive performance should warrant further investigation.

In addition to its role in energy metabolism, GLN and its derivatives have been demonstrated to involve themselves in a variety of cellular events in a glycolysis-independent manner [14]. For example, the involvement of GLN in the O-GlcNAcylation of a variety of proteins should lead to the modulation of a wide range of regulation in cell physiology, including cellular signal transduction, transcription, protein modification and more [14,15]. Given that the O-GlcNAcylation modification is highly prevalent in the mammalian brain and that O-GlcNAcylation has been suggested to regulate many cellular cascades in relation to neurological or neurodegenerative diseases [12,18], what molecules GLN would target in relation to O-GlcNAcylation to mediate the noted enhancement on cognitive performance certainly calls for further endeavors.

Collectively, this study provides clear evidence that GLN does appear to promote both cognitive function and increases in BDNF production in the hippocampus, striatum and cortex. Specifically, GLN treatment significantly facilitates the cAMP/PKA/CREB signaling by increasing CREB5 levels and by decreasing PDE4B levels in the hippocampus, and this possibly leads to the induction of BDNF production to enhance cognitive function.

## 4. Materials and Methods

### 4.1. Chemicals and Reagents

Fetal bovine serum (FBS) was purchased from HyClone (Waltham, MA, USA). Reverse transcriptase and SYBR green reagent were obtained from ThermoFisher (ThermoFisher Scientific, Waltham, MA, USA). Antibodies were purchased from different companies: rabbit monoclonal anti-BDNF (abcam, Cambridge, MA, USA), rabbit polyclonal anti-PDE4B (abcam), mouse monoclonal anti-CREB5 (ThermoFisher Scientific), rabbit monoclonal anti-phospho-CREB antibody (Cell signaling, Danvers, MA, USA) and mouse monoclonal anti-β-actin antibody (Novus, Centennial, CO, USA). Unless otherwise specified, all the other chemicals and reagents used in this study were from Sigma Chemicals (St. Louis, MO, USA).

### 4.2. Animal Ethics and Experiments

Seven-week-old male C57BL/6 mice were obtained from the National Laboratory Animal Center in Taipei, Taiwan. All the animal procedures were approved by the Institutional Animal Care and Use Committee of the National Yang-Ming University (Permit Number 1080203). After daily intraperitoneal (IP) injection of GLN (0, 3, 10 and 30 mg/mouse) for 7 or 14 days, the mice were subjected to the novel object recognition test (NORT). In brief, each individual animal was habituated to an acrylic chamber (40 cm × 30 cm × 20 cm) on three consecutive days, including habituation (20 min), acquisition trial (20 min), and test trial (15 min). During the training, two randomly selected objects were presented to each animal for 20 min. One day after training, another set of objects (one previously presented familiar object (F) and a new novel object (N)) was presented to the trained animals [33]. The time spent exploring each object (F or N) of each animal was recorded with a video device, followed by software analysis (SMART video tracking software 3.0, Panlab (Holliston, MA, USA)).

### 4.3. Cell Culture

The mouse hippocampal cell line HT22 was a generous gift from Dr. David Schubert (Salk Institute, La Jolla, CA, USA) [34]. HT22 cells were maintained in DMEM-high glucose medium with 10% fetal bovine serum, 100 units/mL penicillin and 100 µg/mL streptomycin. HT22 cells were seeded the previous night into 6-well plate to reach 70–80% confluence in the following day, and then the cells were treated with serum-free medium containing different compounds for 6 h to determine mRNA concentrations or for 24 h to measure protein levels in cell lysates.

### 4.4. Determination of Cellular Protein Expression

To extract proteins from treated cells, 200 µL of lysis buffer (50 mM Tris, 5 mM EDTA, 300 mM NaCl, 1% Triton X-100, 5 mM PMSF, 10 µg/mL aprotinin and 10 µg/mL leupeptin-hemisulfate) were used to harvest cells. Cell lysates were scratched down and collected into 1.5 mL eppendorf tubes. Similarly, to collect tissue proteins, 200 µL of lysis buffer was mixed with a tissue of an appropriate size. Then the harvested cells or tissues in lysis buffer were sonicated 2 s for 3 times, followed by centrifugation at 13,500 rpm for 30 min to collect the proteins in the supernatants. Protein concentrations were determined using the Bio-Rad protein assay reagent (Bio-Rad, Hercules, CA, USA). The total protein concentrations were adjusted with 5 × SDS sample loading buffer (312 mM Tris-HCl, 10% SDS, 25% β-mercaptoethanol, 50% glycerol and 0.05% bromophenol blue) and heated to 100 °C for 10 min before regular Western blotting assay. Fifty micrograms of each protein sample was separated on 10% SDS-PAGE, transferred onto a PVDF membrane, blocked with 5% milk at room temperature for 1 h and incubated at 4 °C overnight with various specific antibodies (rabbit monoclonal anti-BDNF (1:1000); rabbit polyclonal anti-PDE4B (1:1000); mouse monoclonal anti-CREB5 (1:1000); rabbit monoclonal anti-phospho-CREB antibody (1:1000); mouse monoclonal anti-β-actin antibody (1:2000) and mouse monoclonal anti-α-tubulin antibody (1:5000), followed by incubation for 2 h with the corresponding horseradish peroxidase-coupled secondary antibodies (1:5000)). After incubation with secondary antibodies, membranes were washed 3 times and the ECL solution (Millipore, Burlington, MA, USA) was added and incubated for 1 min at room temperature. The chemiluminescence signal on the blot was monitored by the GE Amersham Imager 600 (Chicago, IL, USA) and the protein signals were quantified by Multi Gauge 3.0 software (FUJIFILM, Tokyo, Japan).

### 4.5. Measurement of cAMP by Enzyme-Linked Immunosorbent Assay (ELISA)

The concentration of the cAMP in the hippocampal tissue was determined using a cAMP-Glo™ assay kit from Promega (Madison, WI, USA). The assay was performed according to the manufacturer’s instructions.

### 4.6. Measurement of mRNA Concentration by Quantitative Real-Time Polymerase Chain Reaction (RT-PCR)

Total cellular RNAs were extracted from the harvested tissues and treated cells using Tri-reagent according to the manufacturer’s instructions (Sigma). The purified RNA samples were dissolved in RNase-free water and each sample underwent quantitative RT-PCR to measure the levels of mRNAs of various genes. The mouse primer sequences used and the resultant product sizes were: BDNF (75 bp): Forward (F): 5′-TAA ATG AAG TTT ATA CAG TAC AGT GGT TCT ACA-3′, Reverse (R): 5′-AGT TGT GCG CAA ATG ACT GTT T-3′; NGF (nerve growth factor) (212 bp): F: 5′-CAC AGC CAC AGA CAT CAG GGC-3′, R: 5′-CCT GCT TCT CAT CTG TTG TC-3′; NT-3 (79 bp): F: 5′-GGT AGC CAA TAG AAC CTC ACC AC-3′, R: 5′-GTC ACA CAC TGA GTA CTC TCC TC-3′; NT-4 (235 bp); F: 5′-CCC TGC GTC AGT ACT TCT TCG AGA C-3′, R: 5′-CTG GAC GTC AGG CAC GGC CTG TTC-3′; CNTF (ciliary neurotrophic factor) (124 bp): F: 5′-ACA GTG GAC TGT GAG GTC TAT CC-3′, R: 5′-GGA GAC AGA GGC AAG AGT TAA GAG-3′; CREB5 (106 bp): F: 5′-TGT GCC TCC TTG AAA CAA GCC ATT-3′, R: 5′-ACC AGC ATA TGC CCA GAC TG-3′; PDE4B (188 bp): F: 5′-CTG CAG CCT AAC TAC CTG TC-3′, R: 5′-ACA CTT GGT TCC CTG ATC TG-3′ and GAPDH (222 bp): F: 5′-AAG GTC ATC CCA GAG CTG AA-3′, R: 5′-CTG CTT CAC CAC CTT CTT GA-3′. In brief, the reverse transcription was carried out by using 1 µg of total RNAs in RNase-free H_2_O (8.5 µL) and 1 µL oligo dT (0.5 µg/µL) and heated at 70 °C for 5 min. Then the denatured RNAs were mixed with 5 µL 5 × reaction buffer, 2 µL dNTP (10 mM stock), 2.5 µL dithiothreitol (100 mM stock), 0.5 µL Moloney murine leukemia virus reverse transcriptase (200 U/µL) and 0.5 µL RNase inhibitor to a total 25 µL in volume, and then incubated at 42 °C for 60 min, followed by 70 °C for 10 min (MyCylerTM thermal cycler system, Bio-Rad, Hercules, CA, USA). To perform real-time PCR, 2 µL of cDNAs, 0.16 µL of forward and reverse primers (100 µM stock), 8 µL of SYBR Green and appropriate amounts of H_2_O to bring up the total volume to 20 µL were used and transferred into the 8-strip PCR tube. The real-time PCR System (ABI StepOne Plus, ABI QuanStudio 3, Waltham, MA, USA) was used for the PCR reaction. The temperature was set at 95 °C for 2 min for denaturation, followed by the PCR cycle: 2 min at 95 °C for denaturing, 10 s at 60 °C for annealing and 20 s at 72 °C for elongating. The PCR cycle would repeat 40 times to monitor the fluoresce signal of SYBR Green. The threshold cycle (Ct) values for the target genes were normalized with the Ct value of the housekeeping gene GAPDH. Normalization was performed based on the following formula:

relative mRNA expression = 2^−ΔCt^ (ΔCt = Ct^target gene^ − Ct^GAPDH^) (1)

### 4.7. Monitoring the CRE Reporter Activity

To analyze regulation of the CRE-mediated transcription, a minimal promoter sequence bearing a CRE-driven luciferase reporter gene (Addgene, Watertown, MA, USA) was transfected into HT22 cells seeded in 24-well plate and a *p*CMV-β-Gal plasmid was co-transfected as a control. In brief, a mixture including 1 µg CRE reporter plasmid, 0.1 µg of the *p*CMV-β-Gal plasmid and 1 µL P3000^TM^ enhancer reagent (Invitrogen, Waltham, MA, USA) in 25 µL serum-free medium were prepared for each well. Meanwhile, 1 µL of Lipofectamine^TM^ 3000 transfection reagent (Invitrogen) was dissolved in 25 µL serum-free medium for each well. Two parts were then mixed and incubated at room temperature for 30 min. Consequently, 50 µL of the resultant mixture and 200 µL of serum-free medium were added to each well. Cells were then incubated at 37 °C for 4 h, followed by various treatments for an additional 24 h. After the cultured medium was removed, 150 µL of Glo lysis buffer (Promega) was added to collect total cell lysates. After the centrifugation at 13,500 rpm for 10 min, 50 µL of the harvested supernatant was mixed with 50 µL of luciferase substrate (Britelite^TM^, PerkinElmer Inc., Waltham, MA, USA) and the luminescence signal was measured by FB12-single tube luminometer (Berthold Detectin Systems, Level Biotechnology Inc., Taipei, Taiwan). To measure β-galactosidase activity, 50 µL supernatant was mixed with 50 µL substrate ONPG (*O*-nitrophenyl-β-*D*-galactopyranoside) in a 96-well plate and incubated at room temperature for 30 min. The readout by measuring the absorbance at 482 nm wavelength was used to provide the transfection efficiency. The luciferase activity determined was then normalized against the β-galactosidase activity within the same sample. There were triplicate wells of each treatment group of each independent experiment and 4 independent experiments were performed.

### 4.8. Statistical Analysis

Experimental data are expressed as the mean plus/minus the standard deviation (mean ± S.D.) for the indicated number of repeated observations. The results were analyzed using the Student’s *t*-test for two-group comparisons or a one-way analysis of variance followed by the Dunnett’s test, where appropriate, for multiple-group comparisons. In all cases, *p* < 0.05 was regarded as statistically significant.

## Supplementary Materials

The following are available online: Quantitative RT-PCR for TrkB gene in the hippocampus. The purified hippocampal RNA samples from GLN-injected mice underwent quantitative RT-PCR to measure the mRNA levels of TrkB. The mouse primer sequences used and the resultant product sizes were Forward: 5′-CAA GAA CGA GTA TGG GAA GGA TGA G-3′ and Reverse: 5′-TTG GCG TGG TCC AGT CTT CAT A-3′ to give a 107 bp product. Figure S1: No effect of GLN treatment on body weight (BW) of mice. Seven-week-old C57BL/6 male mice were injected with different doses of GLN (0, 3, 10 and 30 mg) for 14 days. The changes in BW were recorded and compared among different GLN treatment groups. The results represent the means ± S.D. (*n* = 8), Figure S2: GLN induction of TrkB mRNA expression in hippocampus. The RNA samples prepared from GLN-injected animal’s hippocampus were subjected to RT-PCR to determine the TrkB mRNA levels using GAPDH as an internal control. The results represent the means ± S.D. (*n* = 8), *, *p* < 0.05 compared with the 0 mg group. Figure S3: No effect of GLN on cell viability of HT22 cells. Plated HT22 cells were treated with GLN (0, 0.001, 0.01, 0.1, 1 and 10 mM) for 24 h and then the 3-(4,5-cimethylthiazol-2-yl)-2,5-diphenyl tetrazolium bromide (MTT) or alamarBlue reagent was included for an additional 4 h. Consequently the readouts were obtained by measuring the OD with a test wavelength at 560 nm and a reference wavelength at 630 nm for MTT, and by monitoring an excitation wavelength at 550 nm and an emission at 590 nm for alamarBlue, respectively. The results represent the means ± S.D. (*n* = 4).
