# Supplementary material for: Glucosamine Enhancement of BDNF Expression and Animal Cognitive Function
Source: Molecules. 2020 Aug 12;25(16):3667. doi: 10.3390/molecules25163667 (PMC7465318; doi:10.3390/molecules25163667)
Supplement: Supplementary file 1 [file molecules-25-03667-s001.zip › molecules-891615 - proofread supplementary/Raw data of the Blots/Figure 2D/Hippocampus/Fig 2D-Hippocampus marker description.pdf]

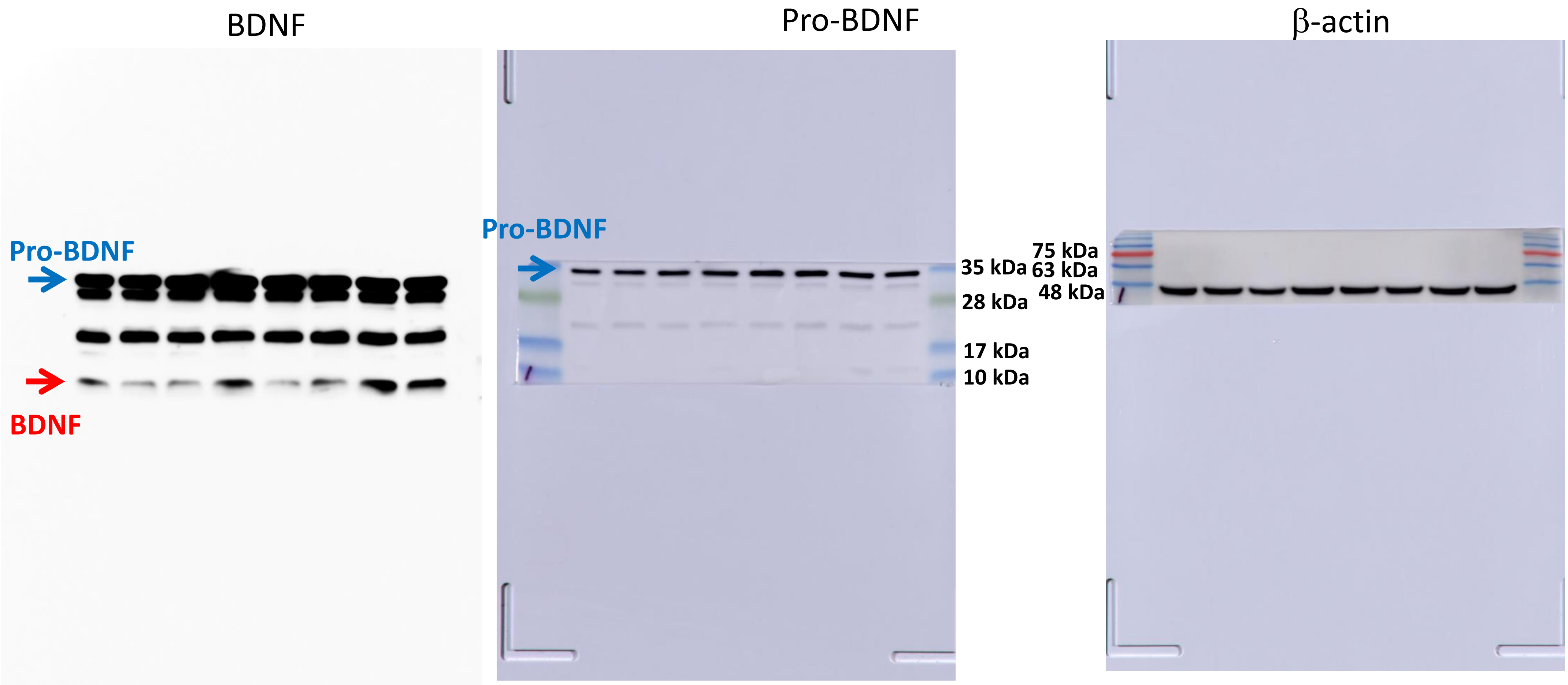

Marker: orange as 75 KDa; green as 28 kDa

Due to the longer exposure (luminescence) to obtain the BDNF image, before updating the software (Amersham imager 680), the image obtained from machine can not be combined with the one from shorter exposure image showing marker (visible light). Therefore, there is no marker on the BDNF image.
