# Supplementary figures and images for: Glucosamine Enhancement of BDNF Expression and Animal Cognitive Function
Source: Molecules. 2020 Aug 12;25(16):3667. doi: 10.3390/molecules25163667 (PMC7465318; doi:10.3390/molecules25163667)

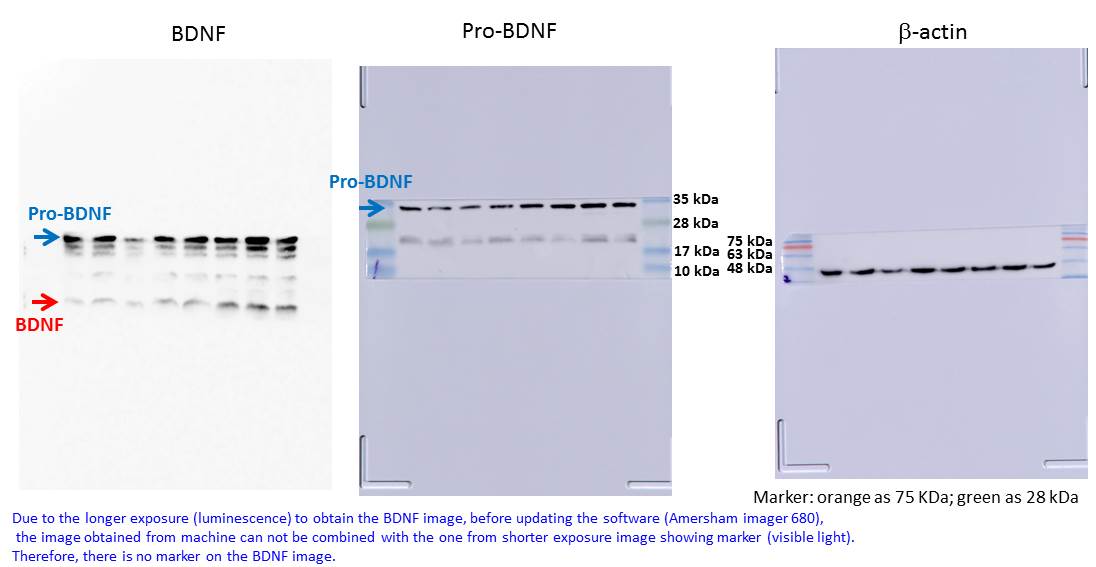

Supplement: Supplementary file 1 [file molecules-25-03667-s001.zip › molecules-891615 - proofread supplementary/Raw data of the Blots/Figure 2D/Cortex/35-Fig 2D-Cortex marker description.jpg]

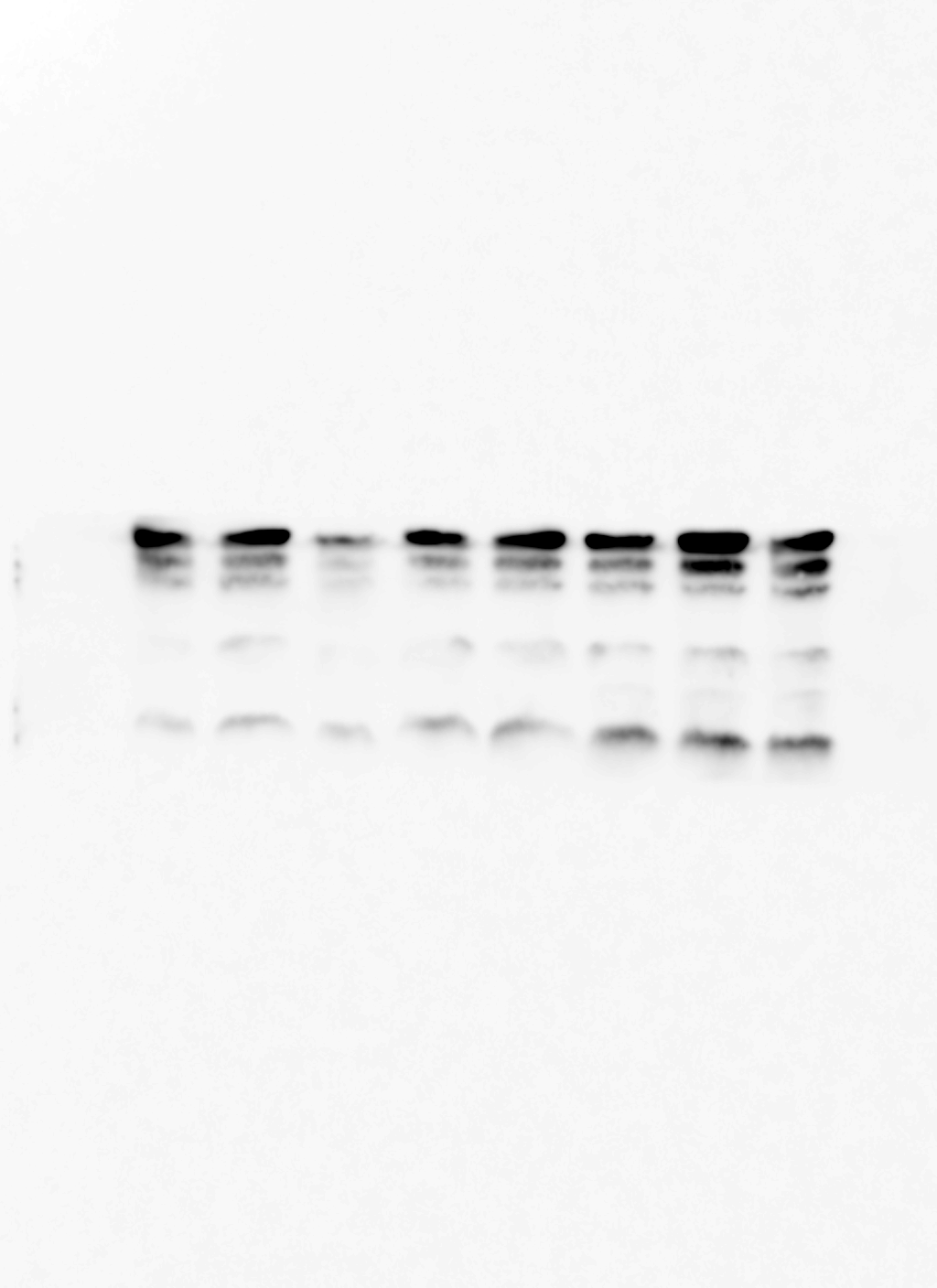

Supplement: Supplementary file 1 [file molecules-25-03667-s001.zip › molecules-891615 - proofread supplementary/Raw data of the Blots/Figure 2D/Cortex/36-Fig 2D-cortex_BDNF-1.bmp]

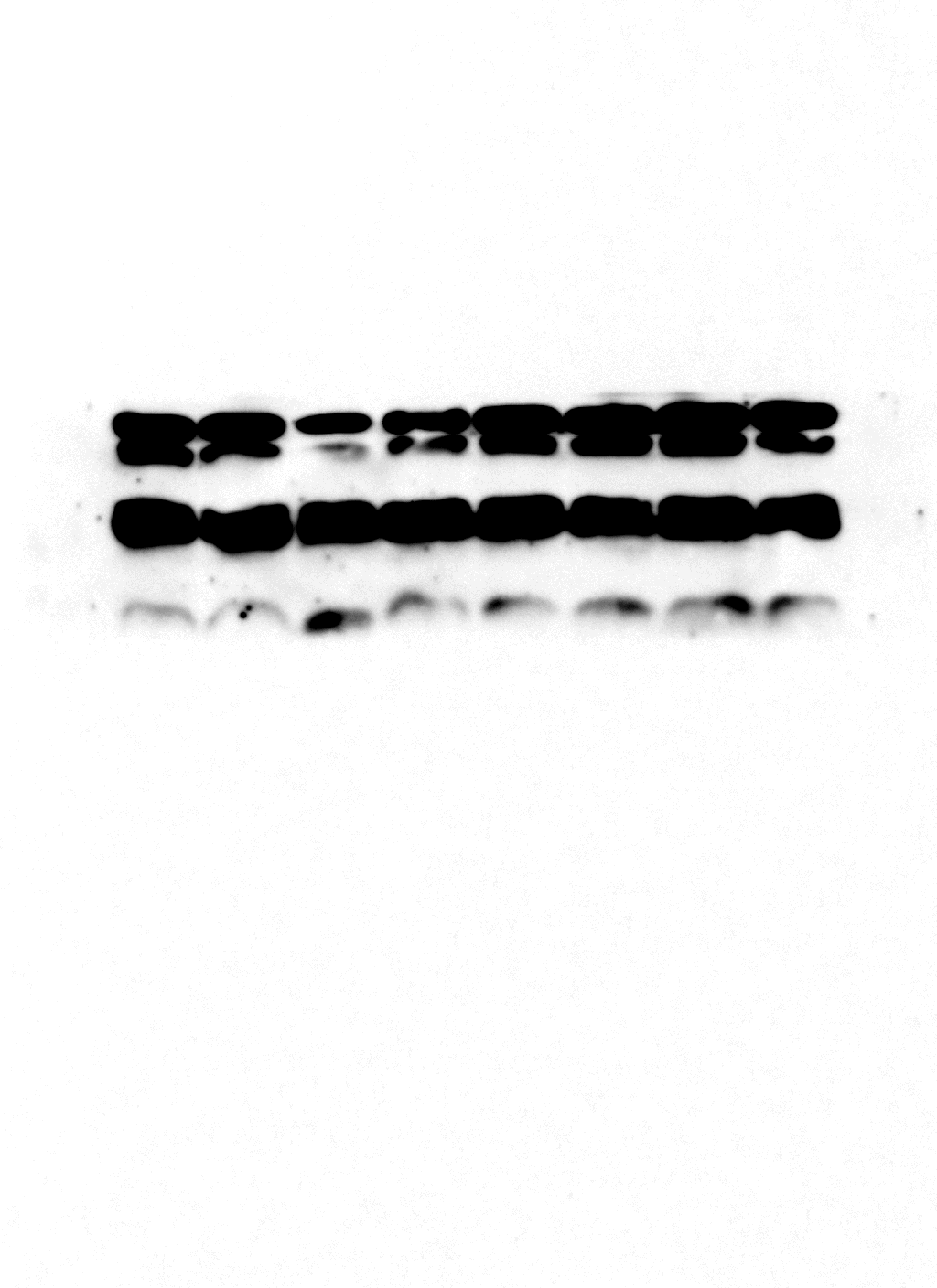

Supplement: Supplementary file 1 [file molecules-25-03667-s001.zip › molecules-891615 - proofread supplementary/Raw data of the Blots/Figure 2D/Cortex/37-Fig 2D-cortex_BDNF-2.bmp]

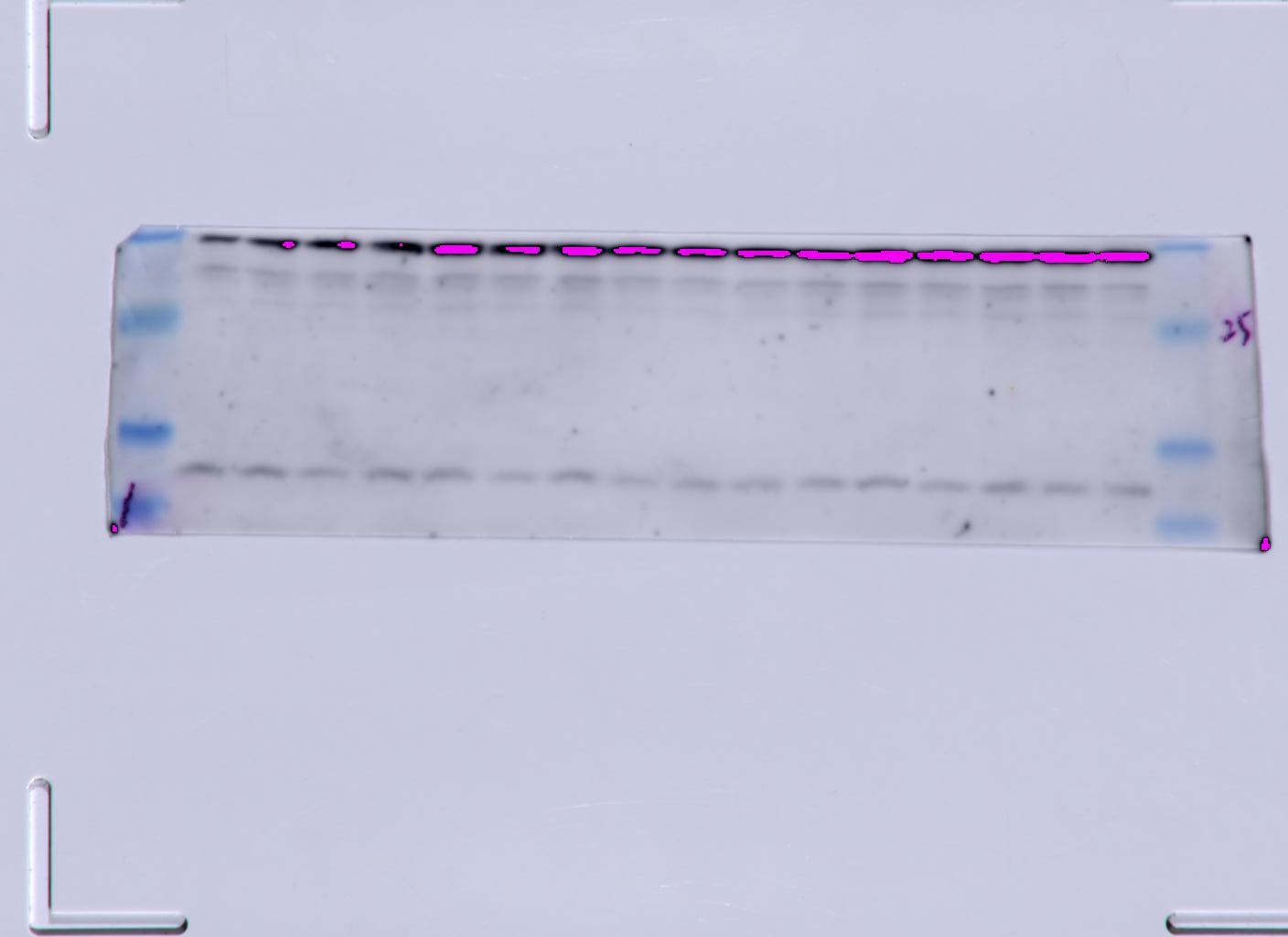

Supplement: Supplementary file 1 [file molecules-25-03667-s001.zip › molecules-891615 - proofread supplementary/Raw data of the Blots/Figure 2D/Cortex/38-Fig 2D-cortex_BDNF-3.jpg]

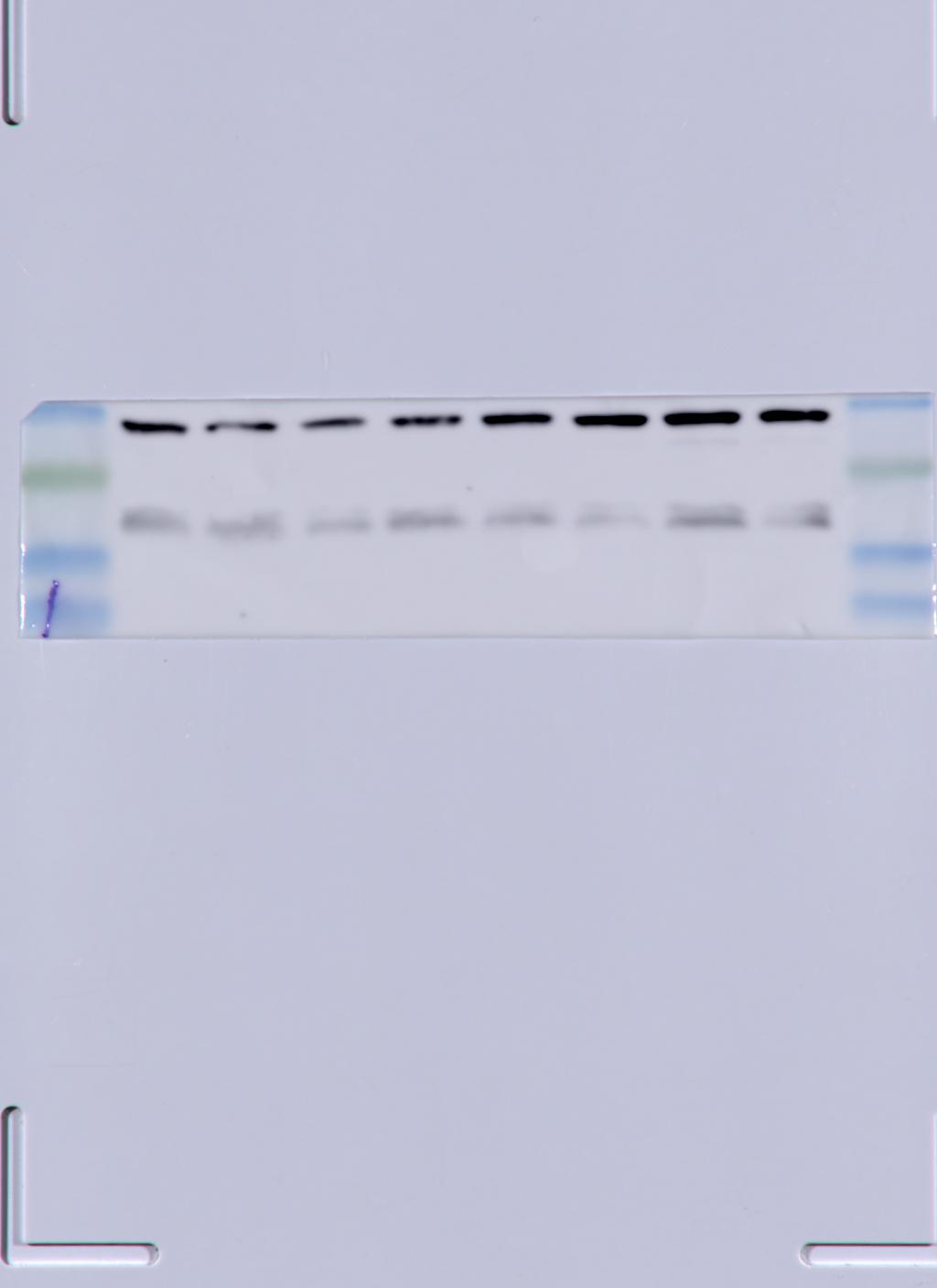

Supplement: Supplementary file 1 [file molecules-25-03667-s001.zip › molecules-891615 - proofread supplementary/Raw data of the Blots/Figure 2D/Cortex/39-Fig 2D-cortex-proBDNF-1.jpg]

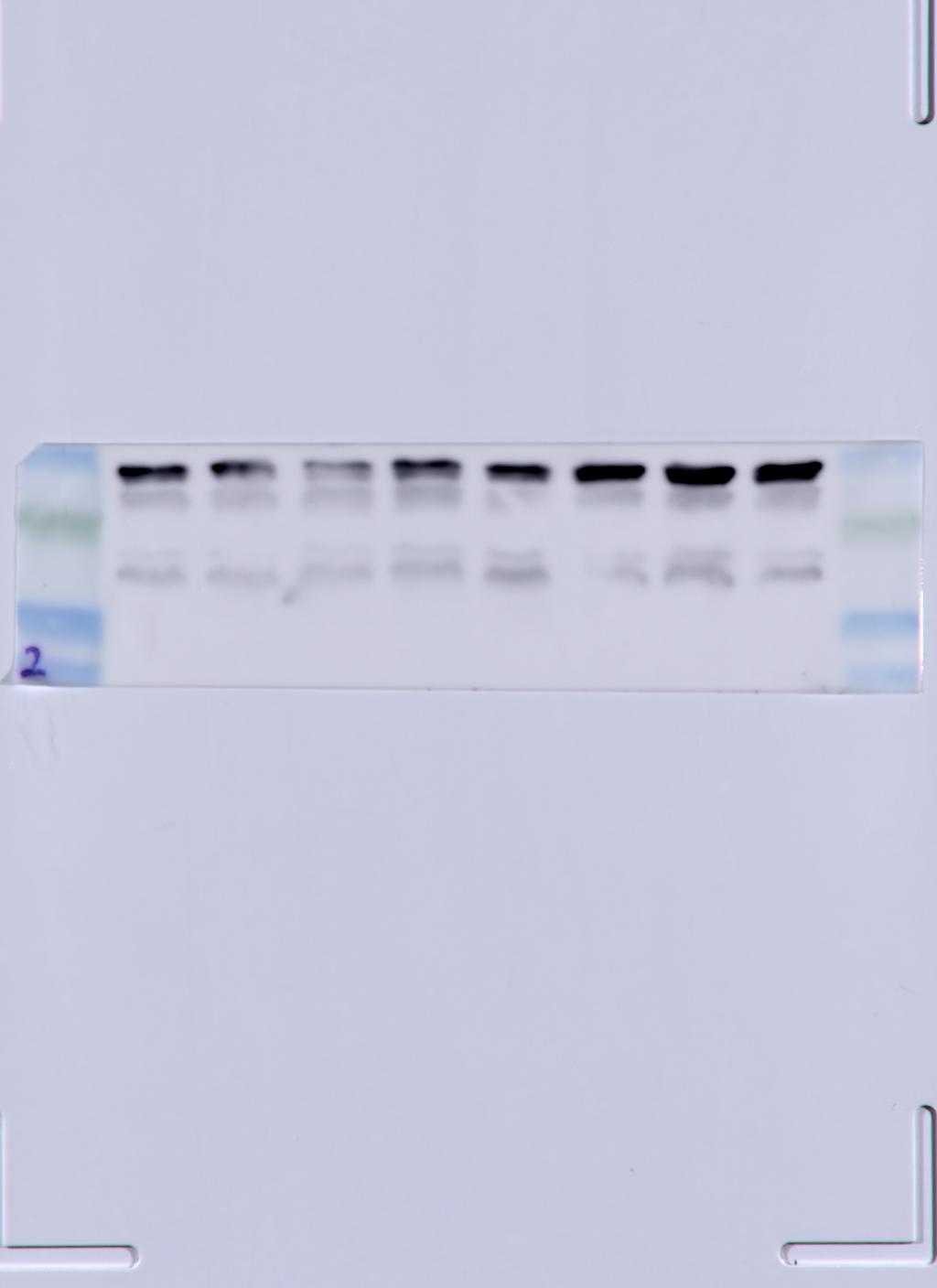

Supplement: Supplementary file 1 [file molecules-25-03667-s001.zip › molecules-891615 - proofread supplementary/Raw data of the Blots/Figure 2D/Cortex/40Fig 2D-cortex_proBDNF-2.jpg]

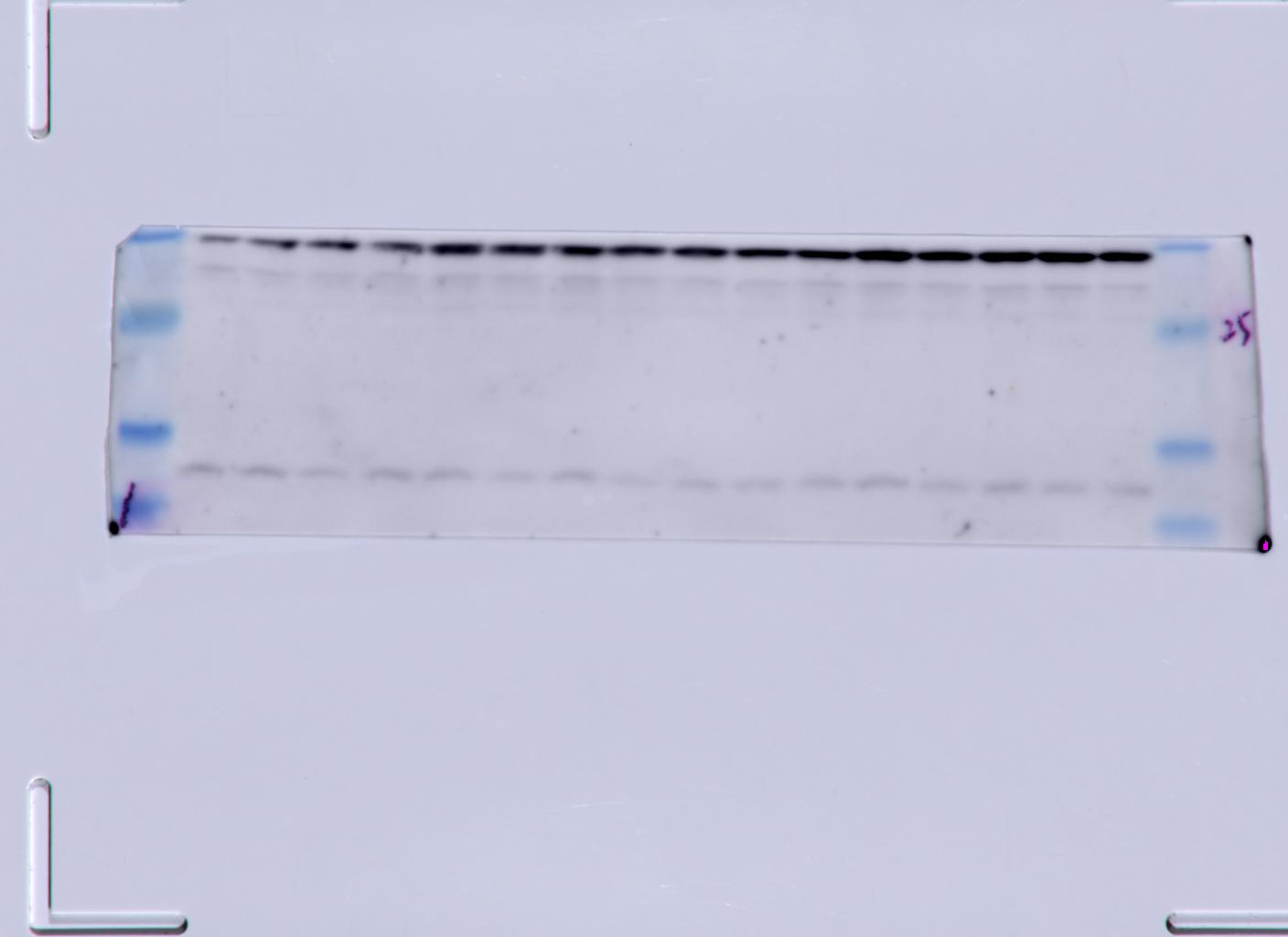

Supplement: Supplementary file 1 [file molecules-25-03667-s001.zip › molecules-891615 - proofread supplementary/Raw data of the Blots/Figure 2D/Cortex/41-Fig 2D-cortex_proBDNF-3.jpg]

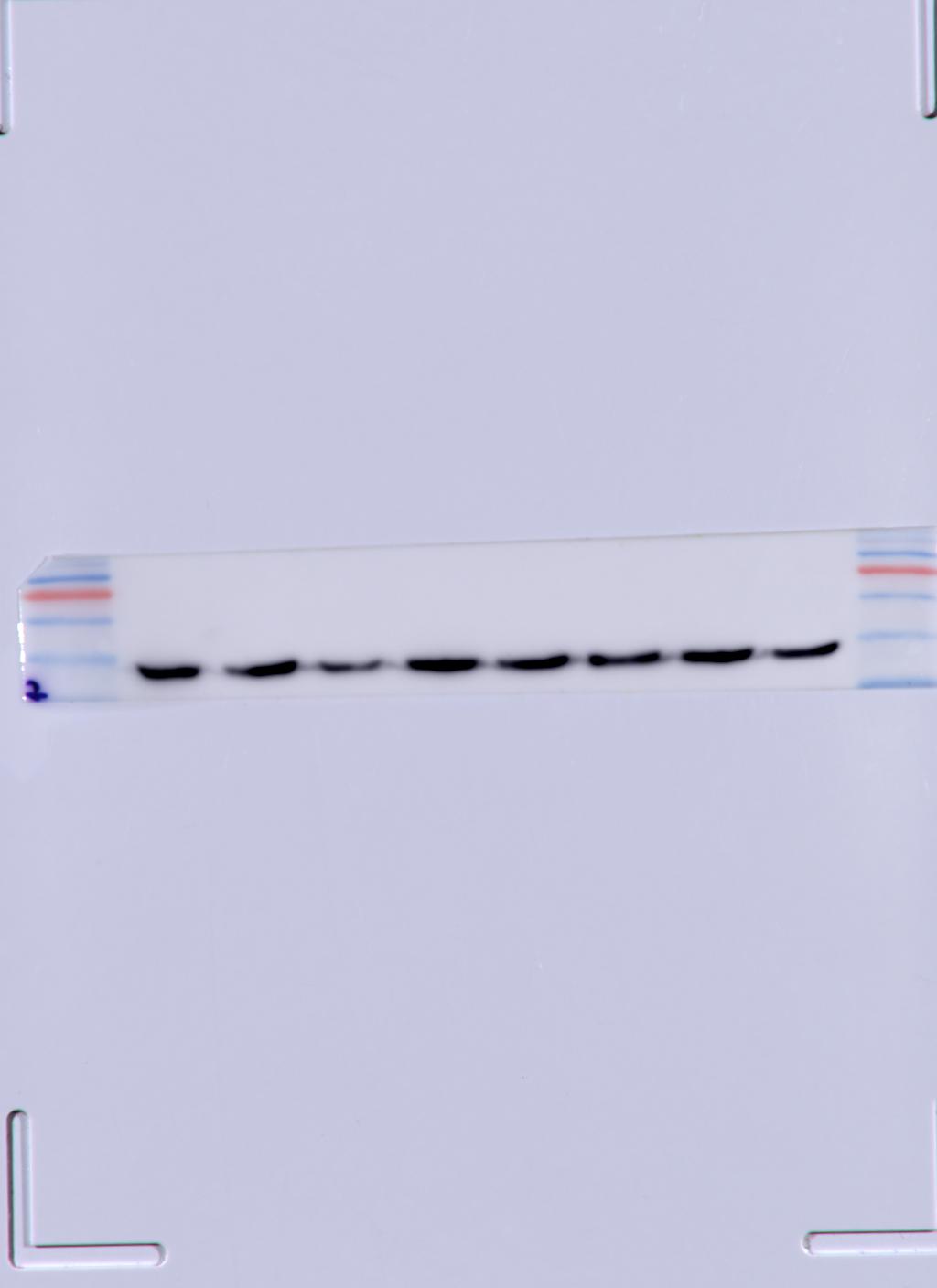

Supplement: Supplementary file 1 [file molecules-25-03667-s001.zip › molecules-891615 - proofread supplementary/Raw data of the Blots/Figure 2D/Cortex/42-Fig 2D-cortex_actin-1.jpg]

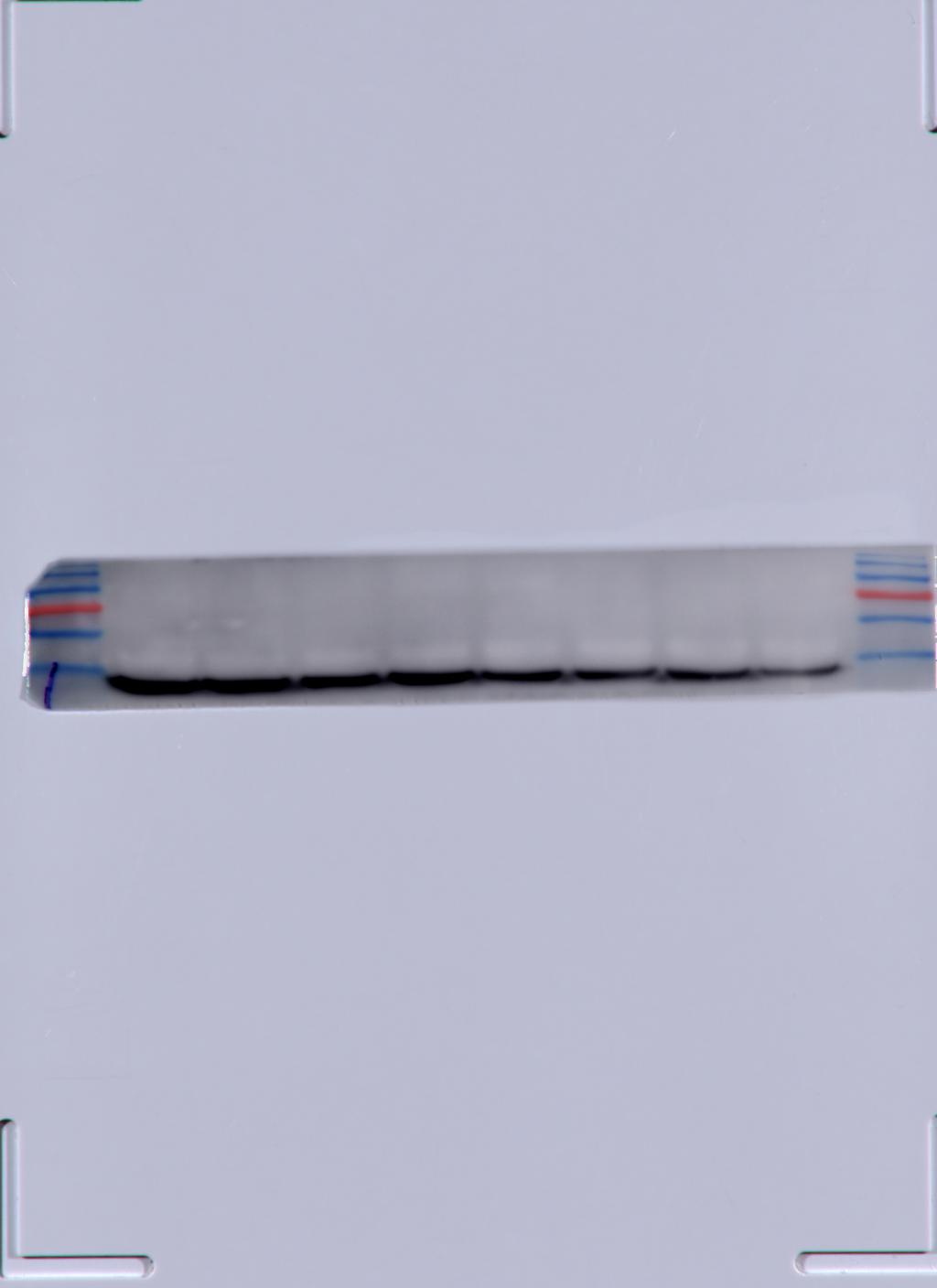

Supplement: Supplementary file 1 [file molecules-25-03667-s001.zip › molecules-891615 - proofread supplementary/Raw data of the Blots/Figure 2D/Cortex/43-Fig 2D-cortex_actin-2.jpg]

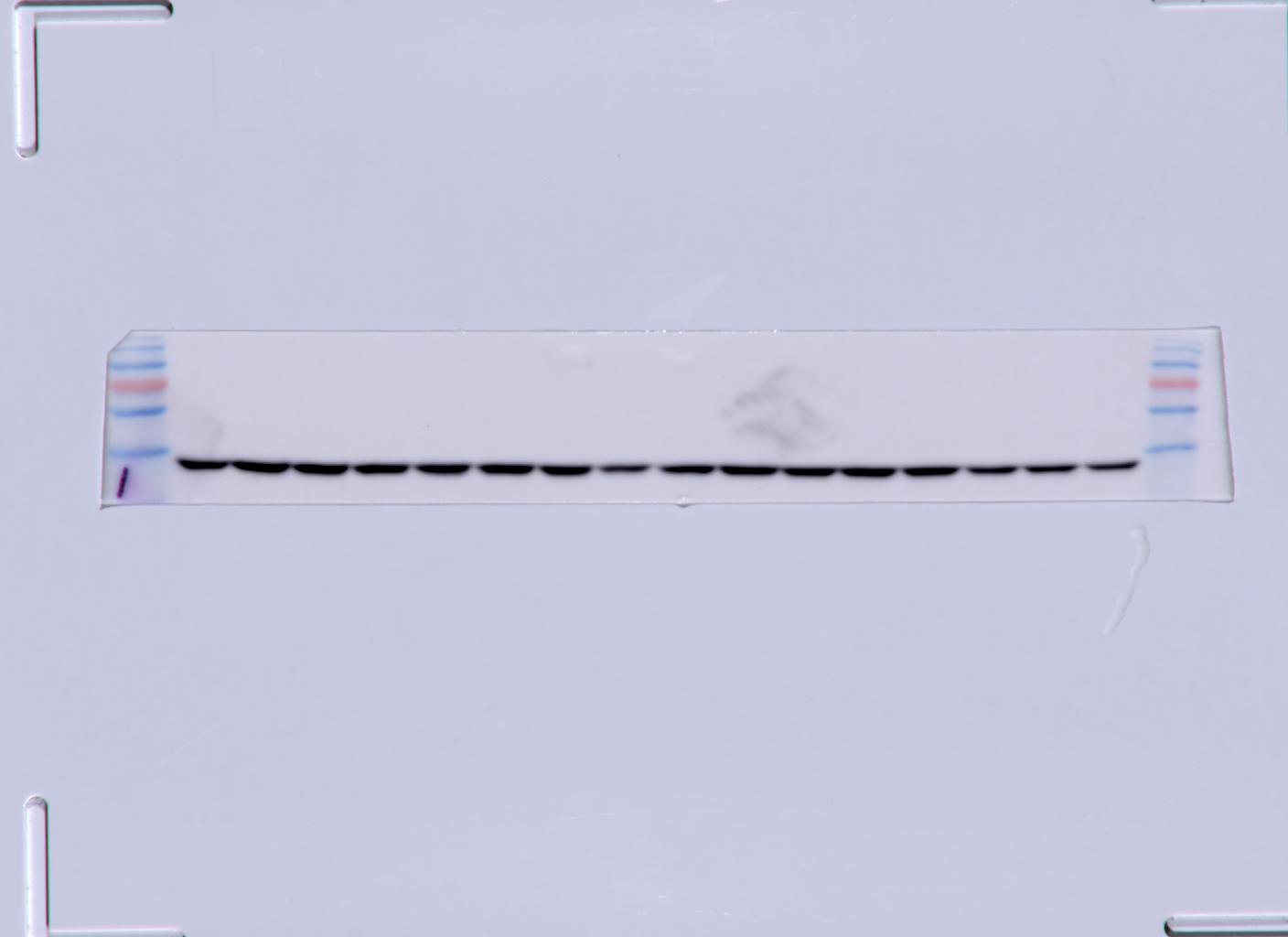

Supplement: Supplementary file 1 [file molecules-25-03667-s001.zip › molecules-891615 - proofread supplementary/Raw data of the Blots/Figure 2D/Cortex/44-Fig 2D-cortex_actin-3.jpg]

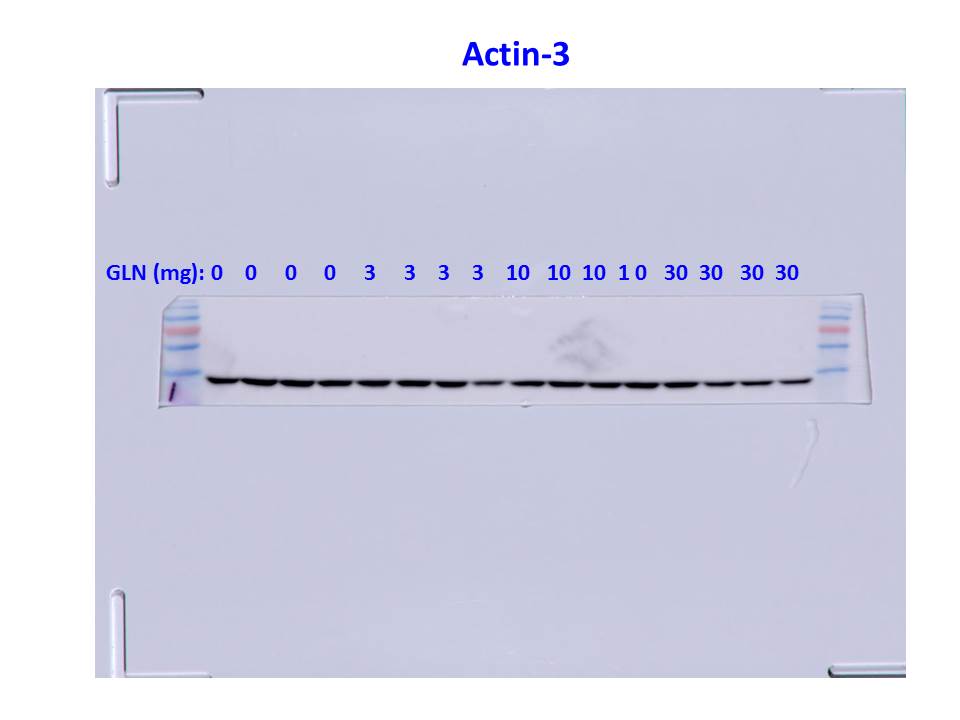

Supplement: Supplementary file 1 [file molecules-25-03667-s001.zip › molecules-891615 - proofread supplementary/Raw data of the Blots/Figure 2D/Cortex/45-Fig 2D-Description for 16 samples-cortex-actin-3.jpg]

# Actin-3

GLN (mg): 0 0 0 0 3 3 3 3 10 10 10 10 30 30 30 30

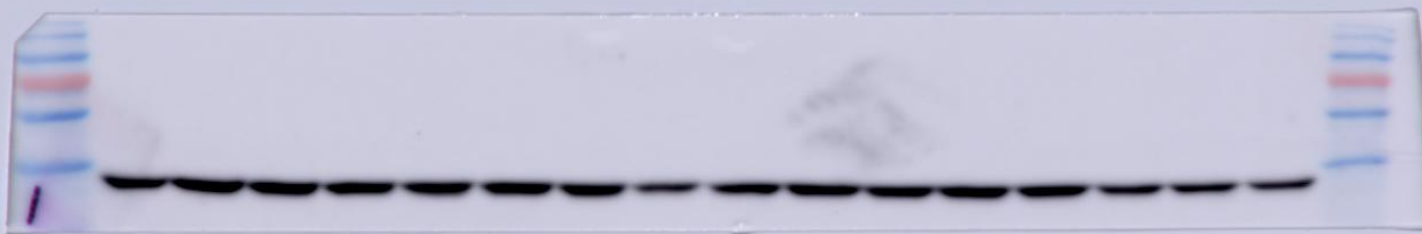

Supplement: Supplementary file 1 [file molecules-25-03667-s001.zip › molecules-891615 - proofread supplementary/Raw data of the Blots/Figure 2D/Cortex/Fig 2D-Description for 16 samples-cortex-actin-3.pdf]

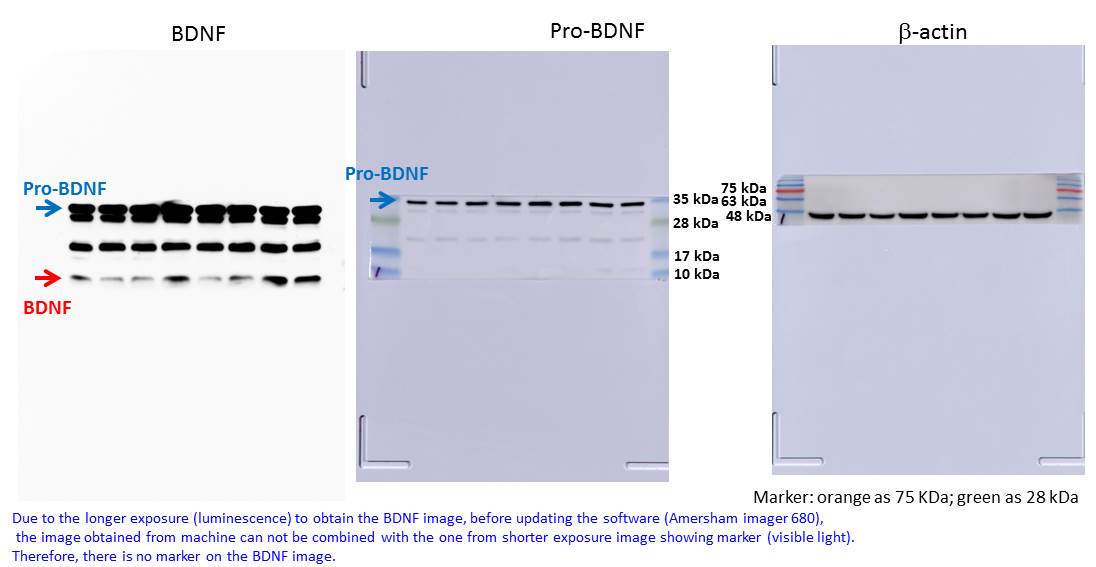

Supplement: Supplementary file 1 [file molecules-25-03667-s001.zip › molecules-891615 - proofread supplementary/Raw data of the Blots/Figure 2D/Hippocampus/13-Fig 2D-Hippocampus marker description.jpg]

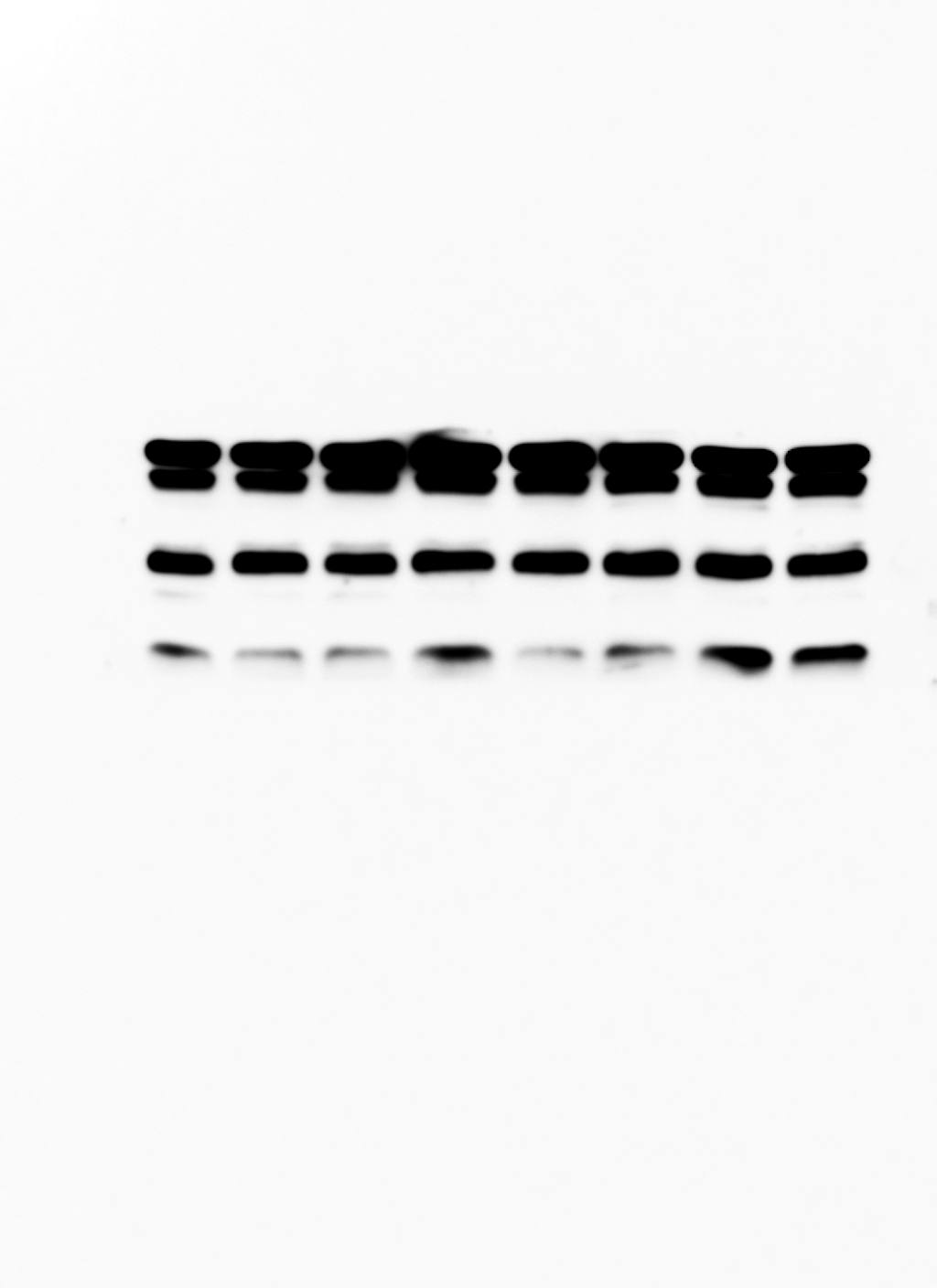

Supplement: Supplementary file 1 [file molecules-25-03667-s001.zip › molecules-891615 - proofread supplementary/Raw data of the Blots/Figure 2D/Hippocampus/14-Fig 2D-hippocampus-BDNF-1.bmp]

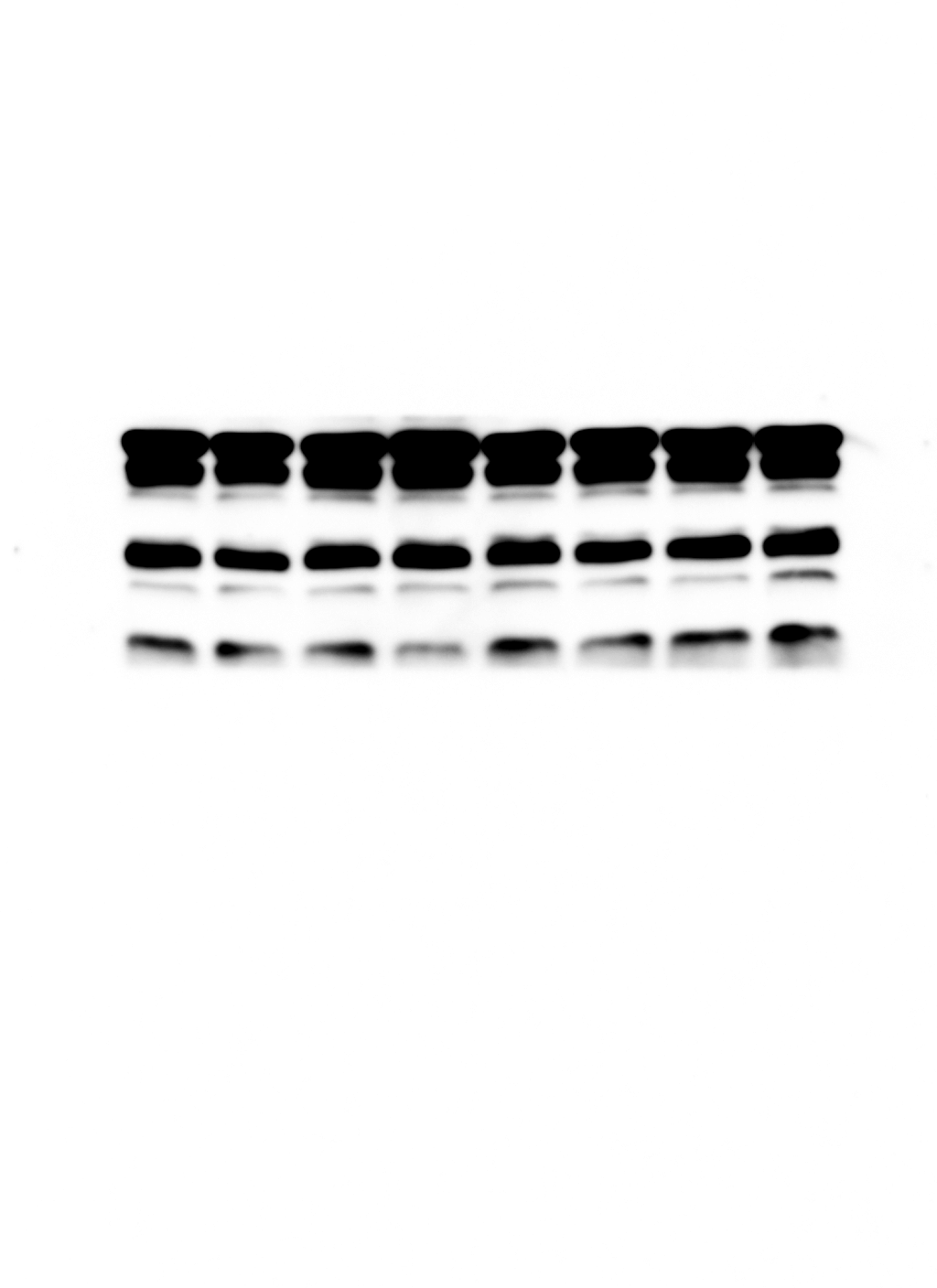

Supplement: Supplementary file 1 [file molecules-25-03667-s001.zip › molecules-891615 - proofread supplementary/Raw data of the Blots/Figure 2D/Hippocampus/15-Fig 2D-hippocampus-BDNF-2.bmp]

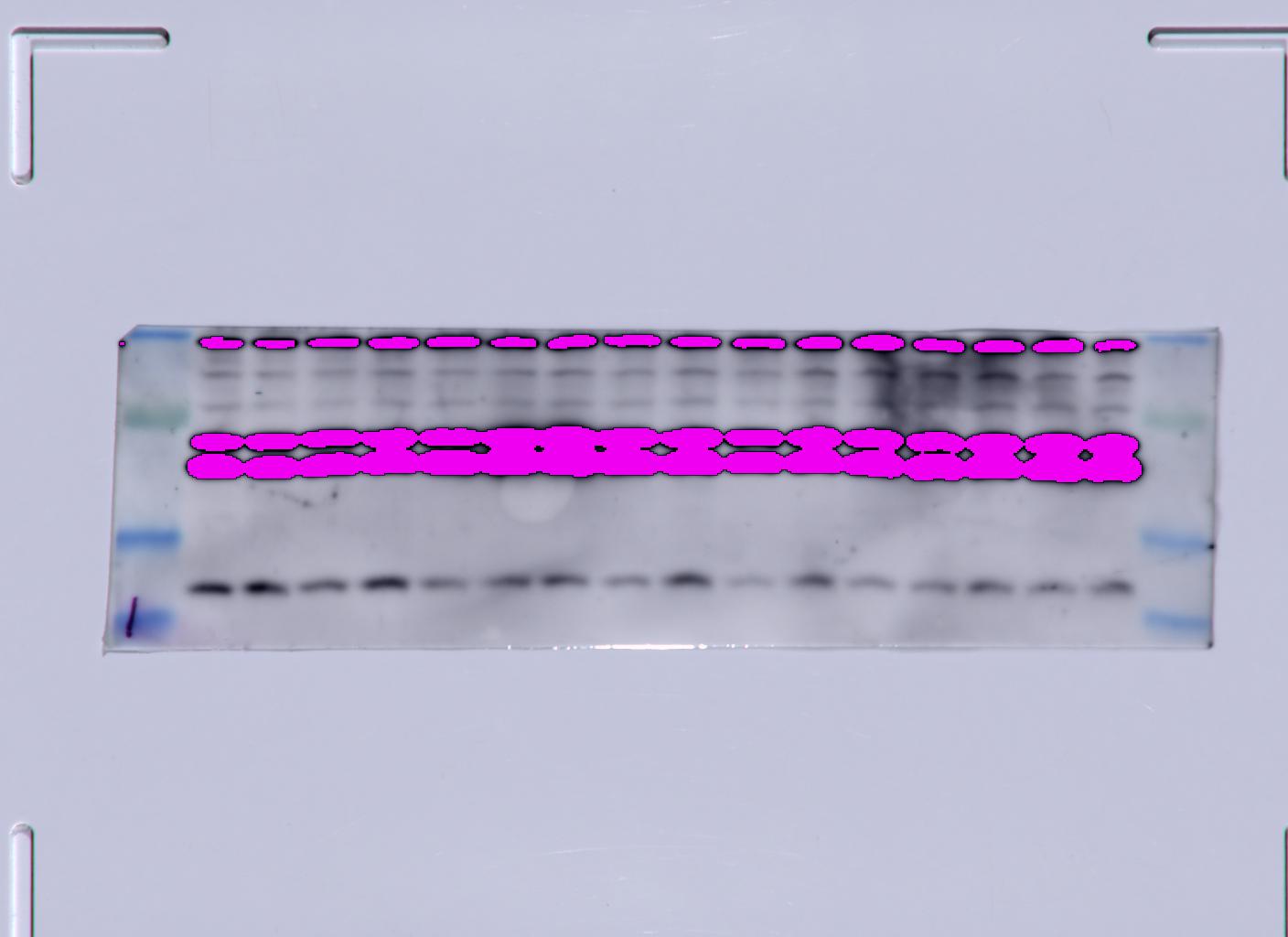

Supplement: Supplementary file 1 [file molecules-25-03667-s001.zip › molecules-891615 - proofread supplementary/Raw data of the Blots/Figure 2D/Hippocampus/16-Fig 2D-hippocampus-BDNF-3.jpg]

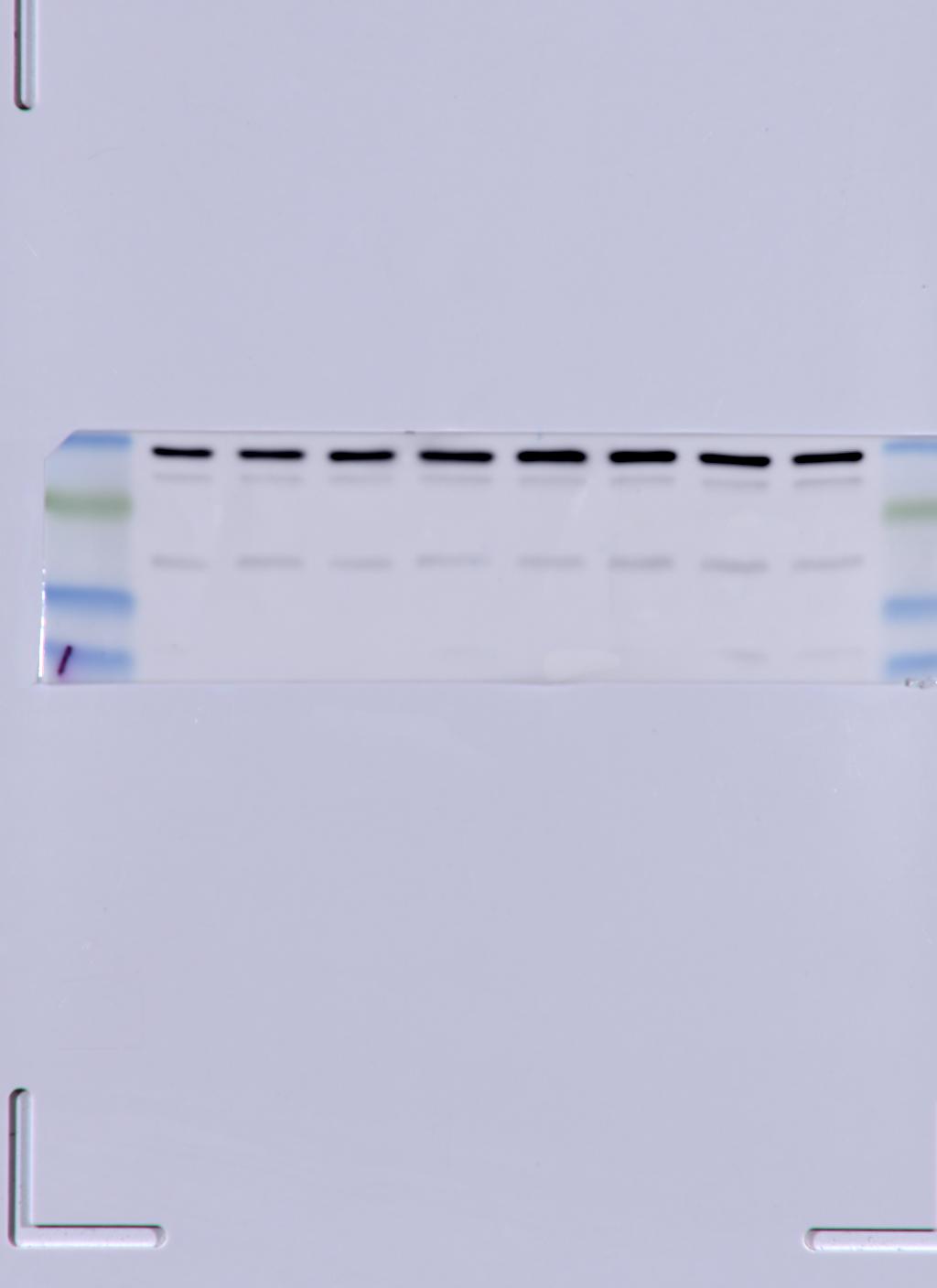

Supplement: Supplementary file 1 [file molecules-25-03667-s001.zip › molecules-891615 - proofread supplementary/Raw data of the Blots/Figure 2D/Hippocampus/17-Fig 2D-hippocampus-proBDNF-1.jpg]

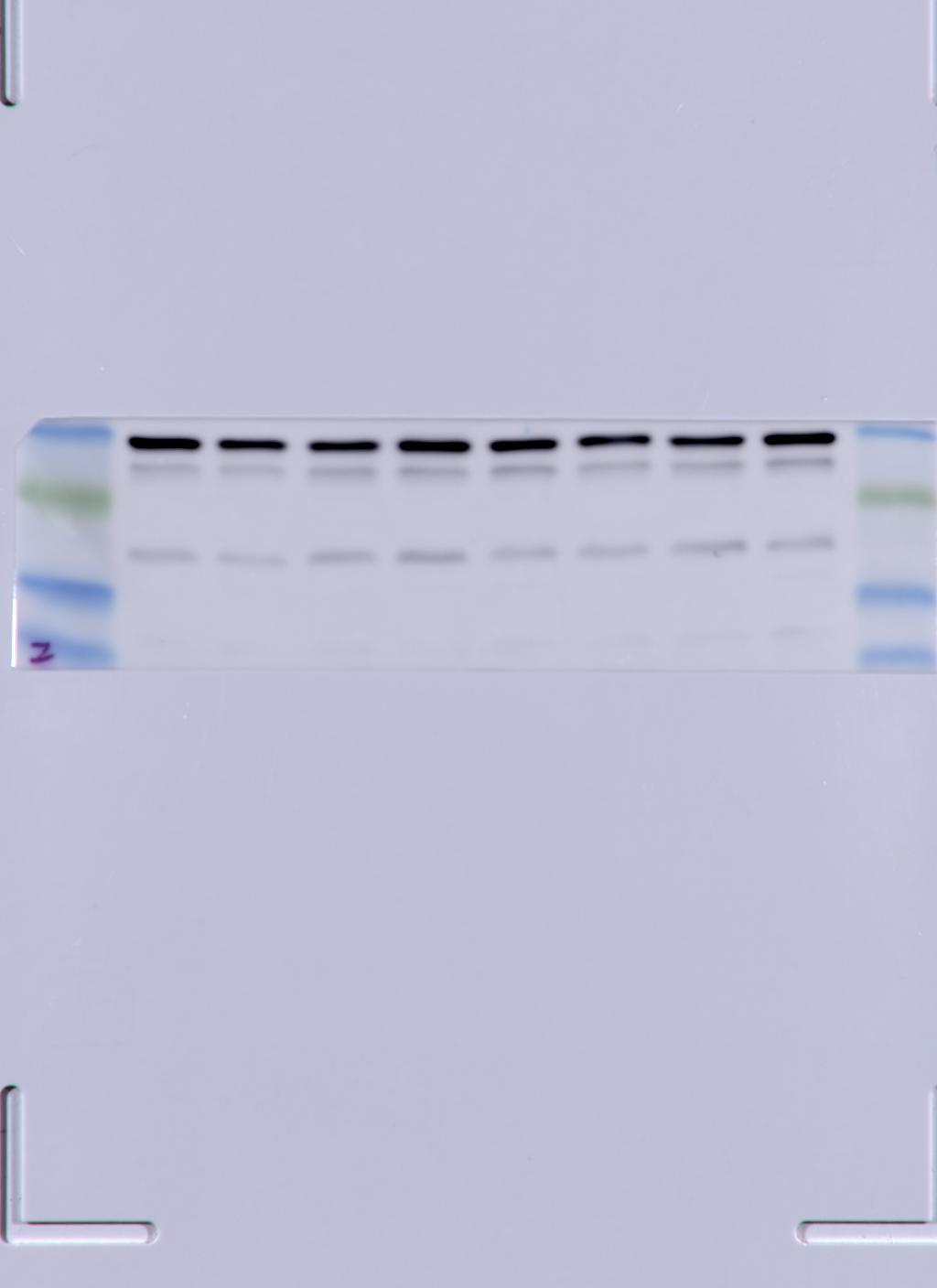

Supplement: Supplementary file 1 [file molecules-25-03667-s001.zip › molecules-891615 - proofread supplementary/Raw data of the Blots/Figure 2D/Hippocampus/18-Fig 2D-hippocampus-proBDNF-2.jpg]

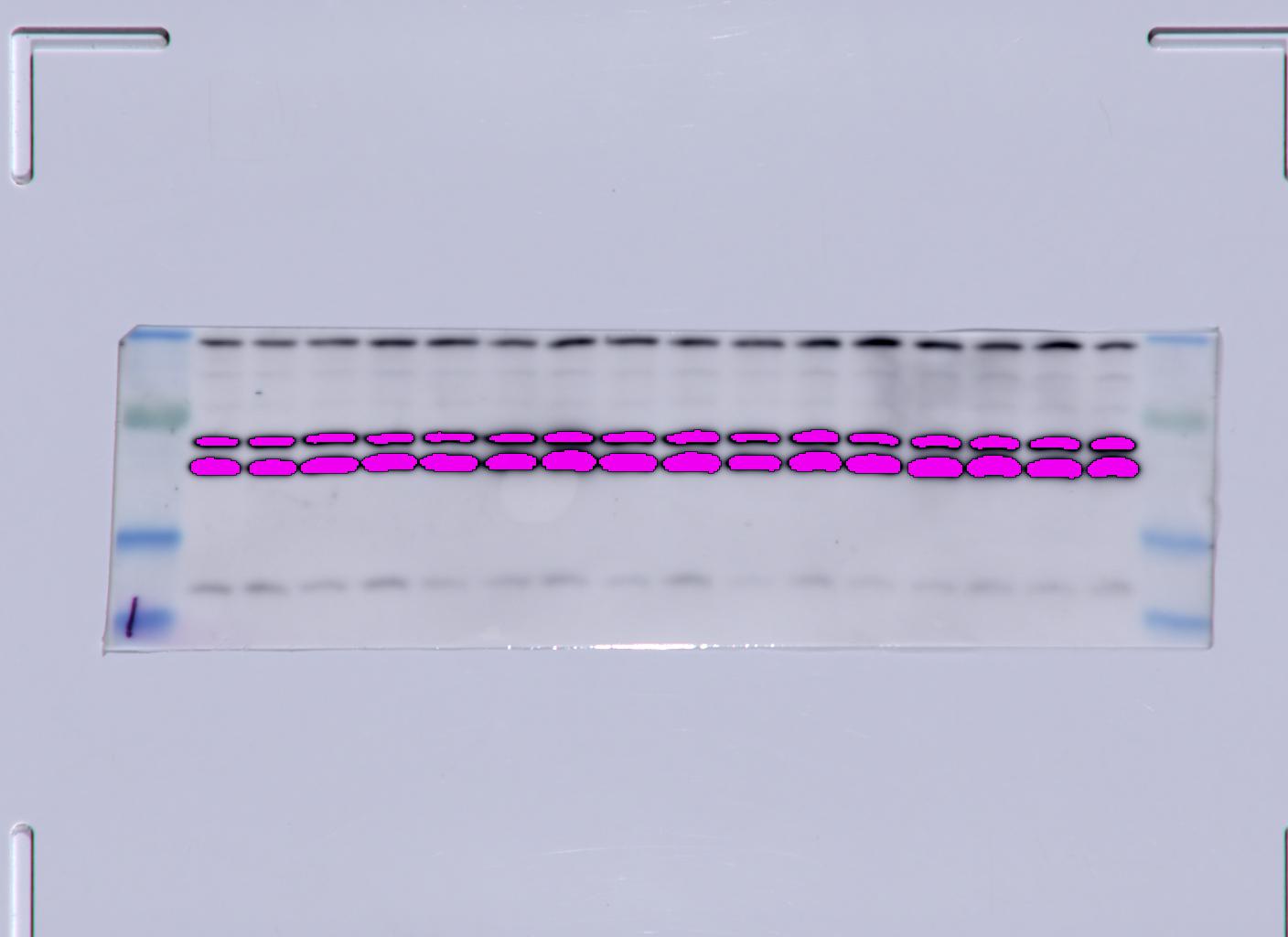

Supplement: Supplementary file 1 [file molecules-25-03667-s001.zip › molecules-891615 - proofread supplementary/Raw data of the Blots/Figure 2D/Hippocampus/19-Fig 2D-hippocampus-proBDNF-3.jpg]

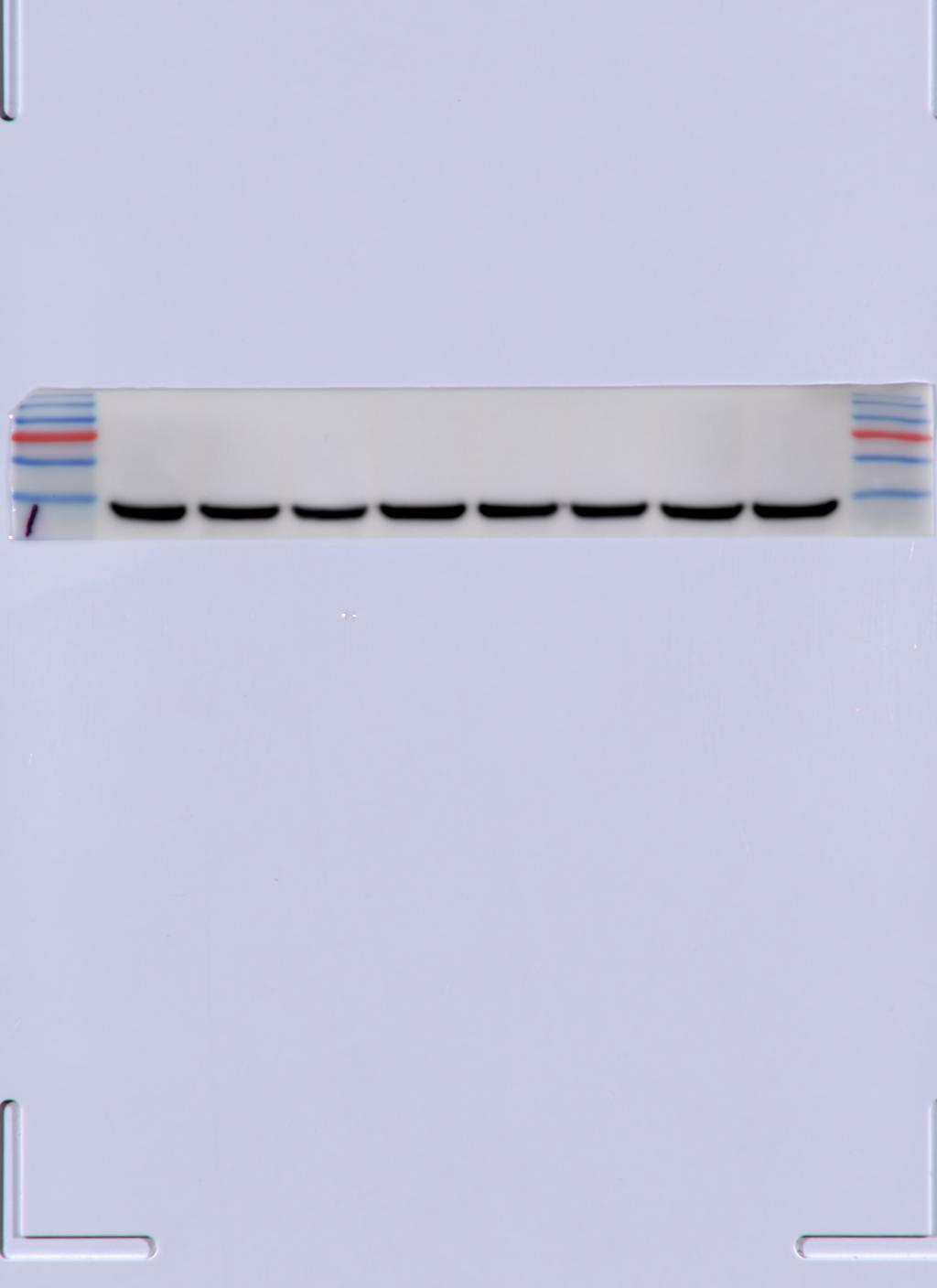

Supplement: Supplementary file 1 [file molecules-25-03667-s001.zip › molecules-891615 - proofread supplementary/Raw data of the Blots/Figure 2D/Hippocampus/20-Fig 2D-hipppocampus-actin-1.jpg]

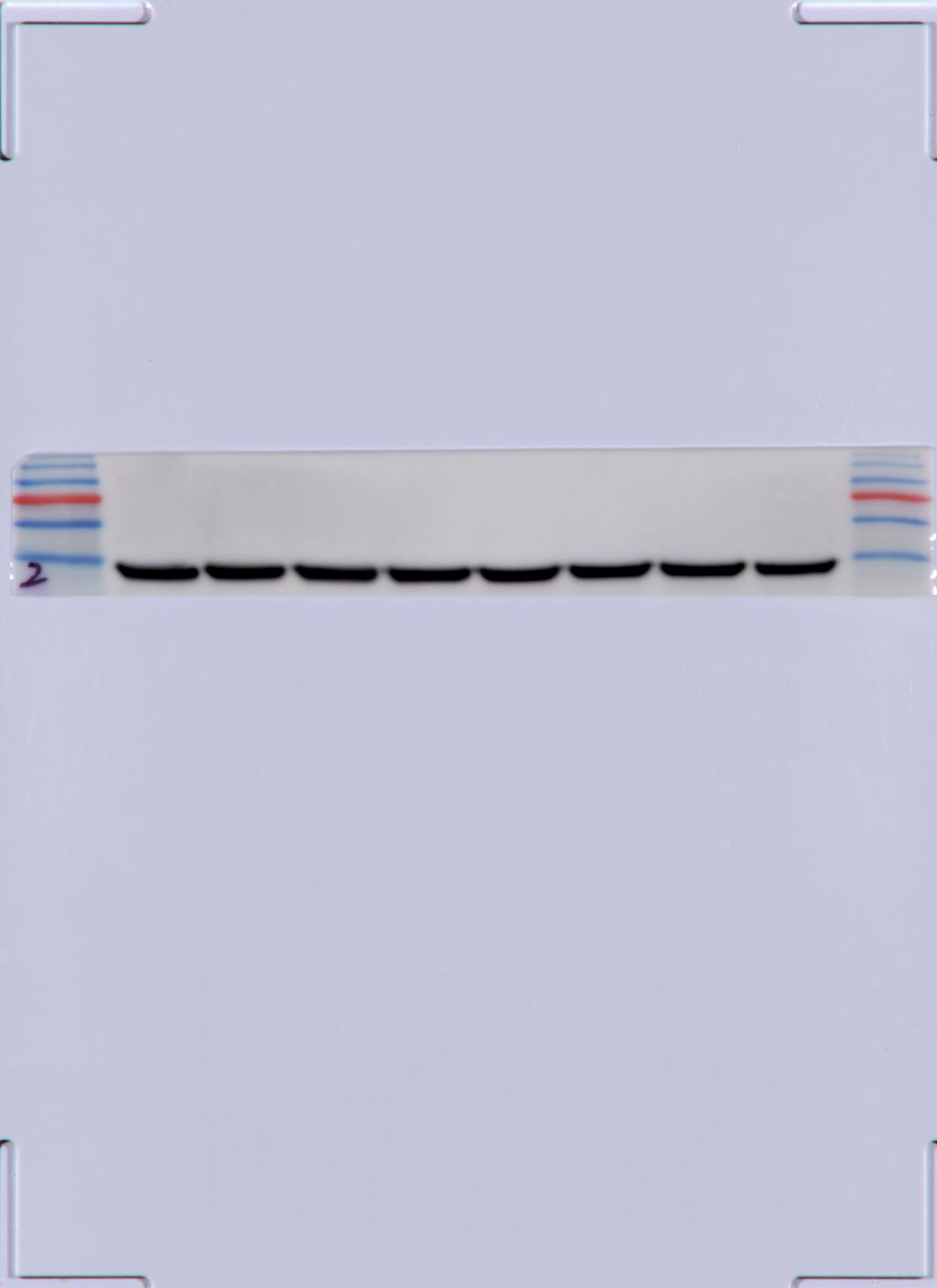

Supplement: Supplementary file 1 [file molecules-25-03667-s001.zip › molecules-891615 - proofread supplementary/Raw data of the Blots/Figure 2D/Hippocampus/21-Fig 2D-hippocampus-actin-2.jpg]

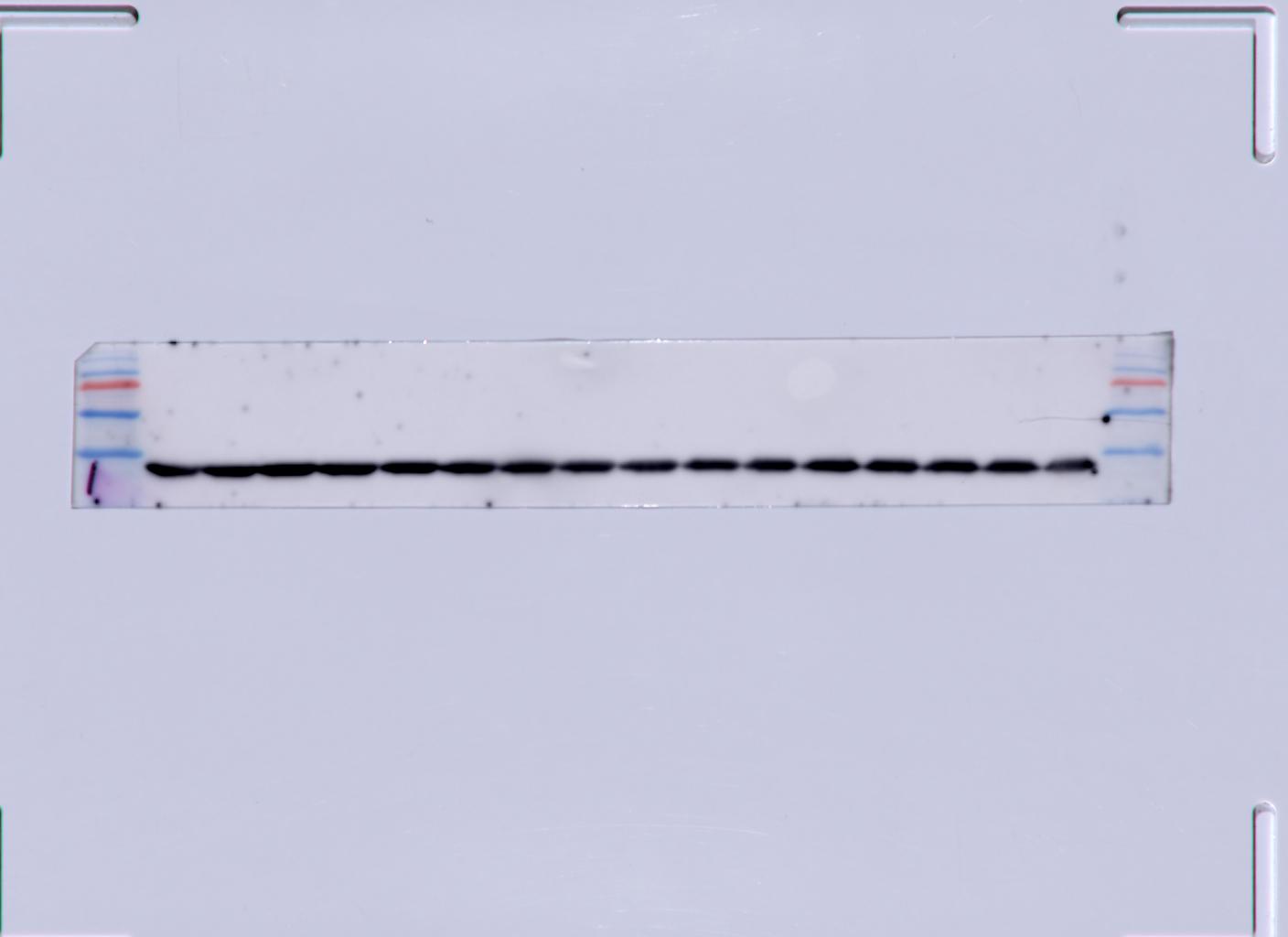

Supplement: Supplementary file 1 [file molecules-25-03667-s001.zip › molecules-891615 - proofread supplementary/Raw data of the Blots/Figure 2D/Hippocampus/22-Fig 2D-hippocampus-actin-3.jpg]

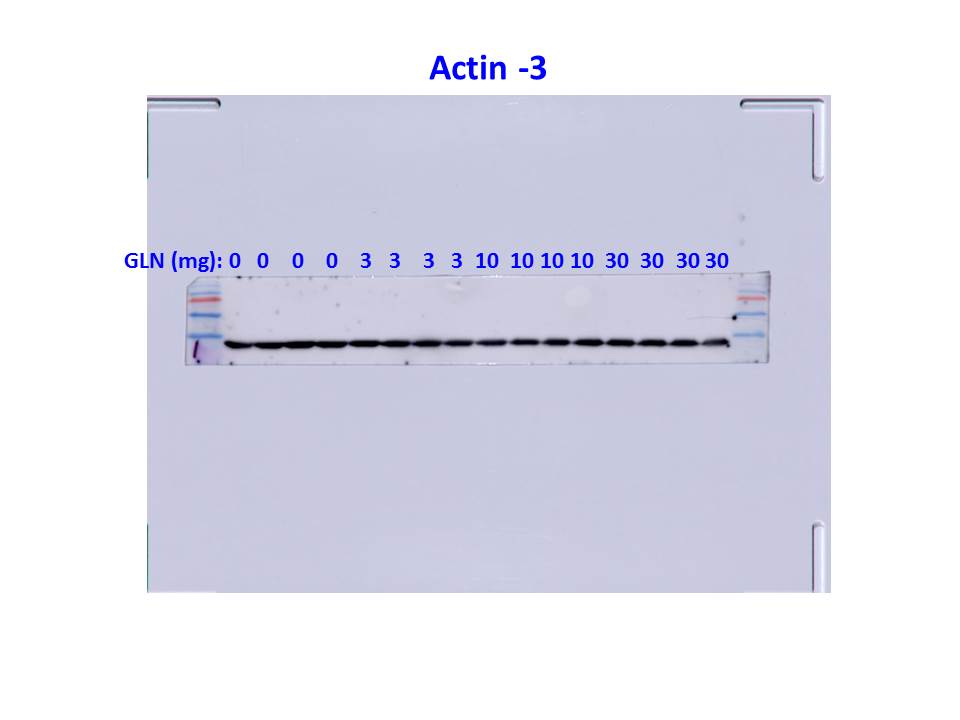

Supplement: Supplementary file 1 [file molecules-25-03667-s001.zip › molecules-891615 - proofread supplementary/Raw data of the Blots/Figure 2D/Hippocampus/23-Fig 2D-Description-hippocampus with 16 samples-Actin.jpg]

## Actin -3

GLN (mg): 0 0 0 0 3 3 3 3 10 10 10 10 30 30 30 30

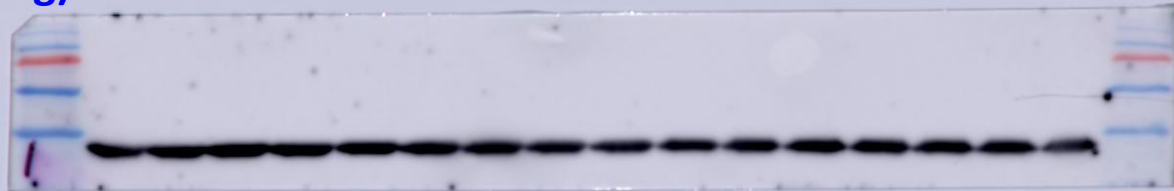

Supplement: Supplementary file 1 [file molecules-25-03667-s001.zip › molecules-891615 - proofread supplementary/Raw data of the Blots/Figure 2D/Hippocampus/Fig 2D-Description-hippocampus with 16 samples-actin.pdf]

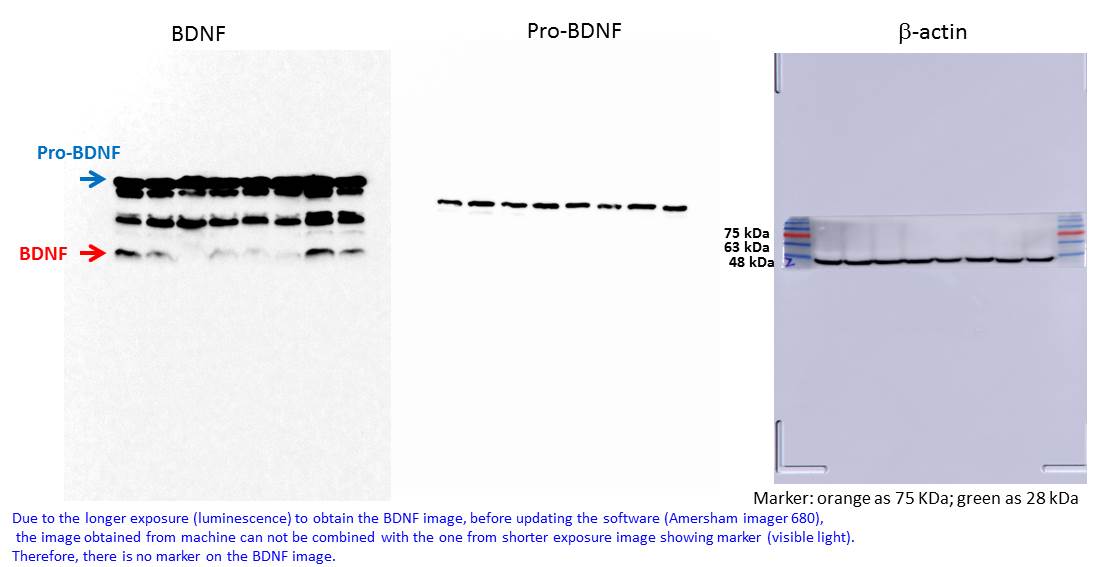

Supplement: Supplementary file 1 [file molecules-25-03667-s001.zip › molecules-891615 - proofread supplementary/Raw data of the Blots/Figure 2D/Striatum/24-Fig 2D-Striatum marker description.jpg]

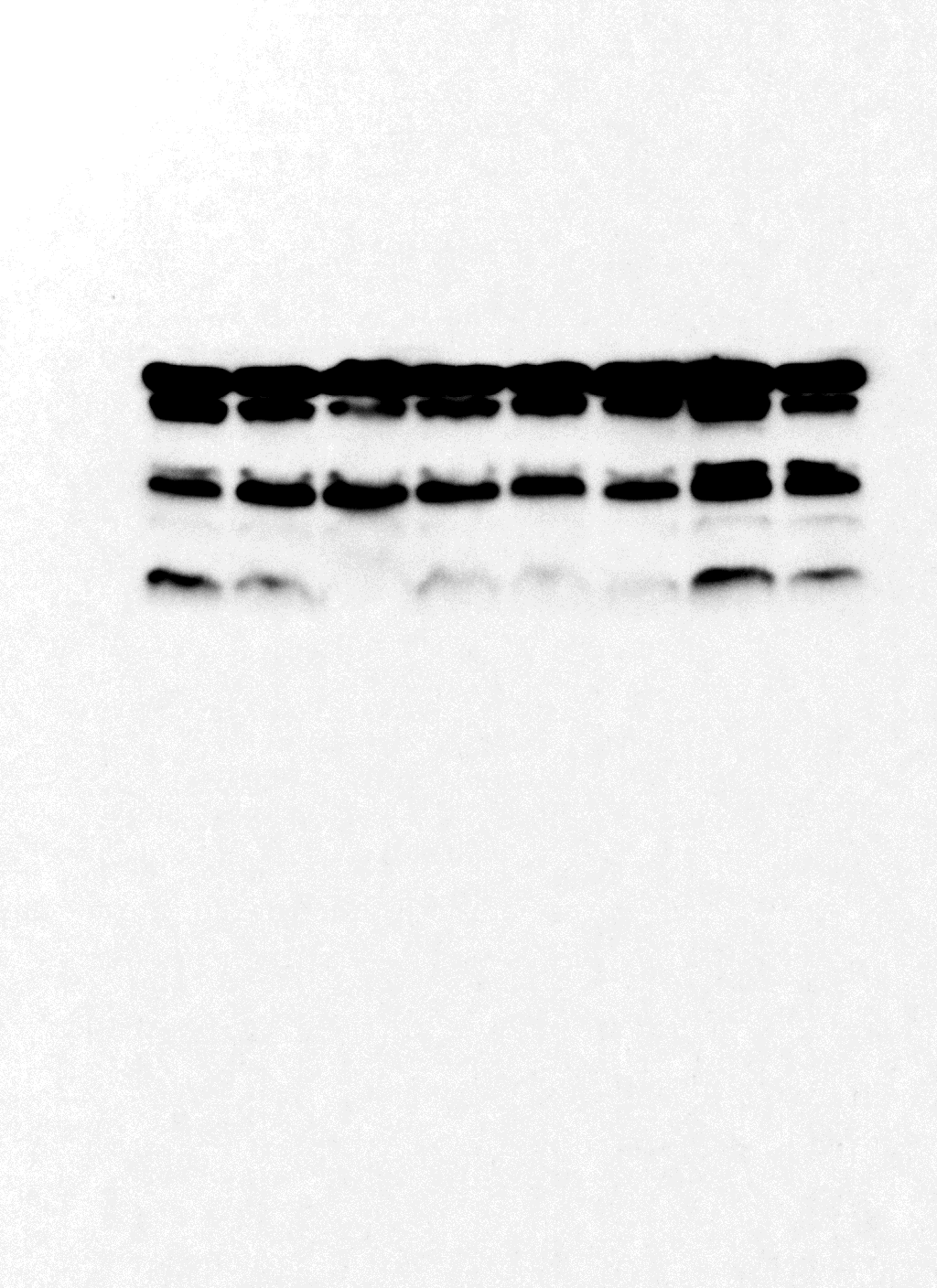

Supplement: Supplementary file 1 [file molecules-25-03667-s001.zip › molecules-891615 - proofread supplementary/Raw data of the Blots/Figure 2D/Striatum/25-Fig 2D-striatum_BDNF-1.bmp]

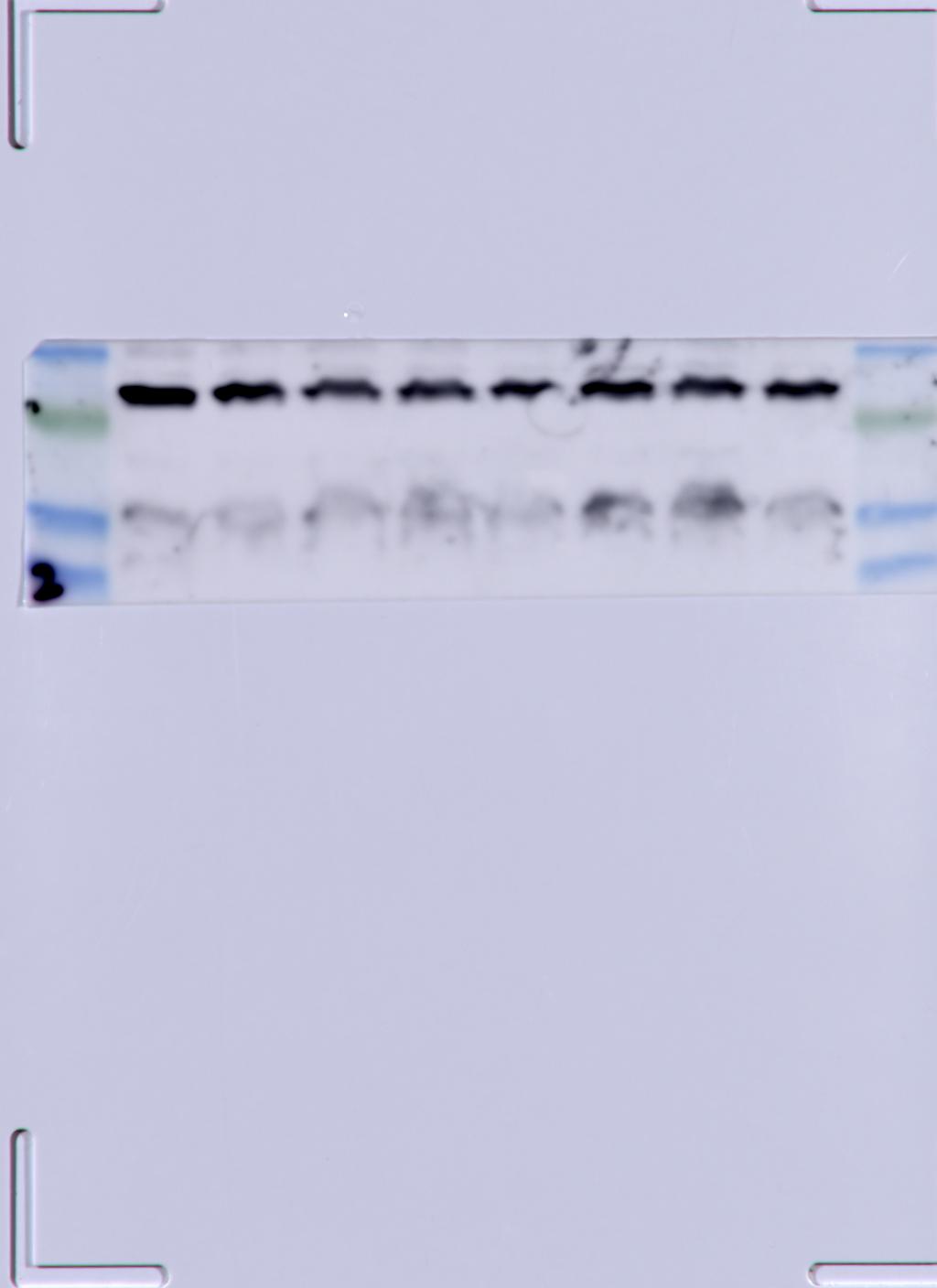

Supplement: Supplementary file 1 [file molecules-25-03667-s001.zip › molecules-891615 - proofread supplementary/Raw data of the Blots/Figure 2D/Striatum/26-Fig 2D-striatum_BDNF-2.jpg]

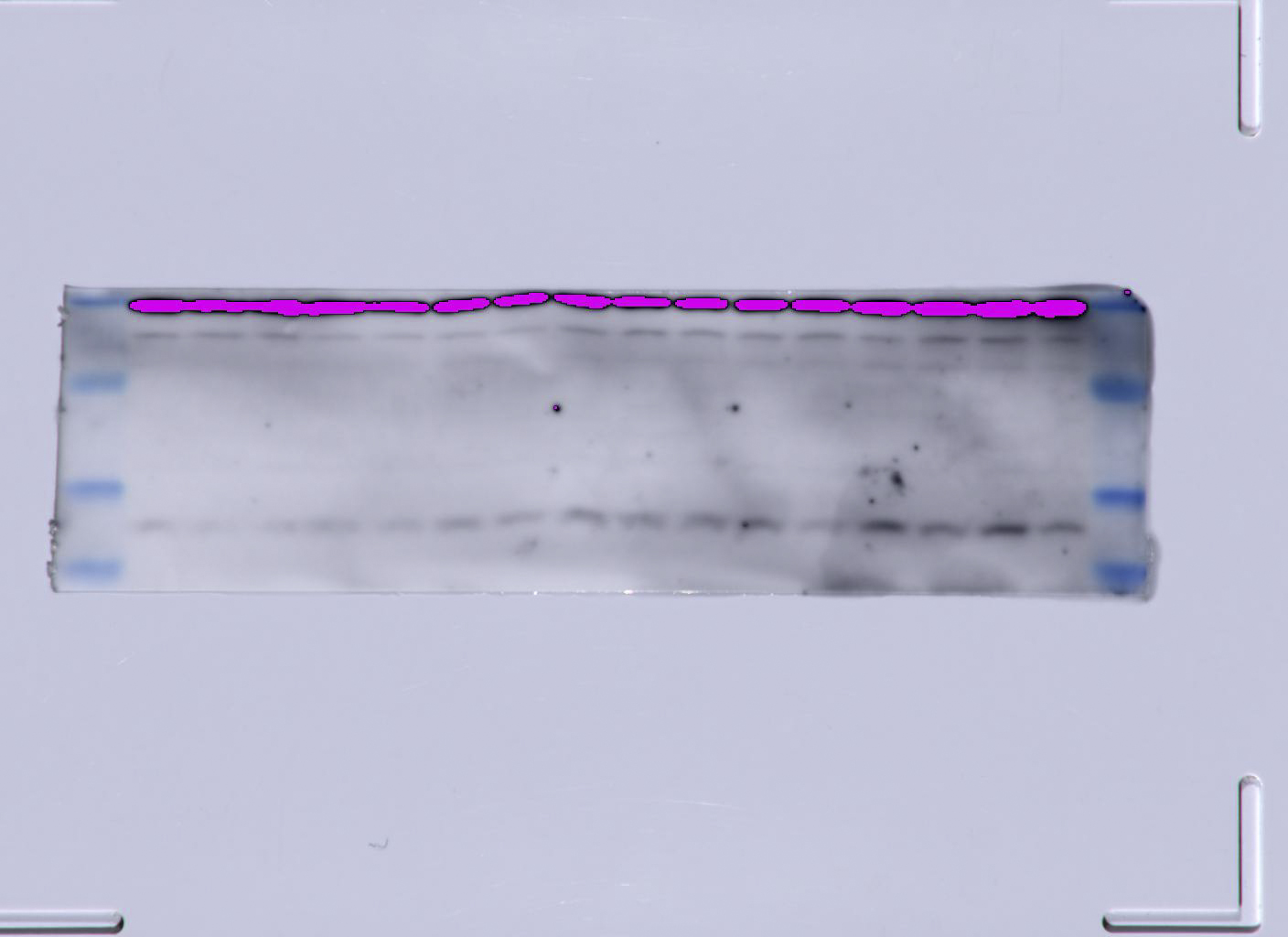

Supplement: Supplementary file 1 [file molecules-25-03667-s001.zip › molecules-891615 - proofread supplementary/Raw data of the Blots/Figure 2D/Striatum/27-Fig 2D-striatum_BDNF-3.jpg]

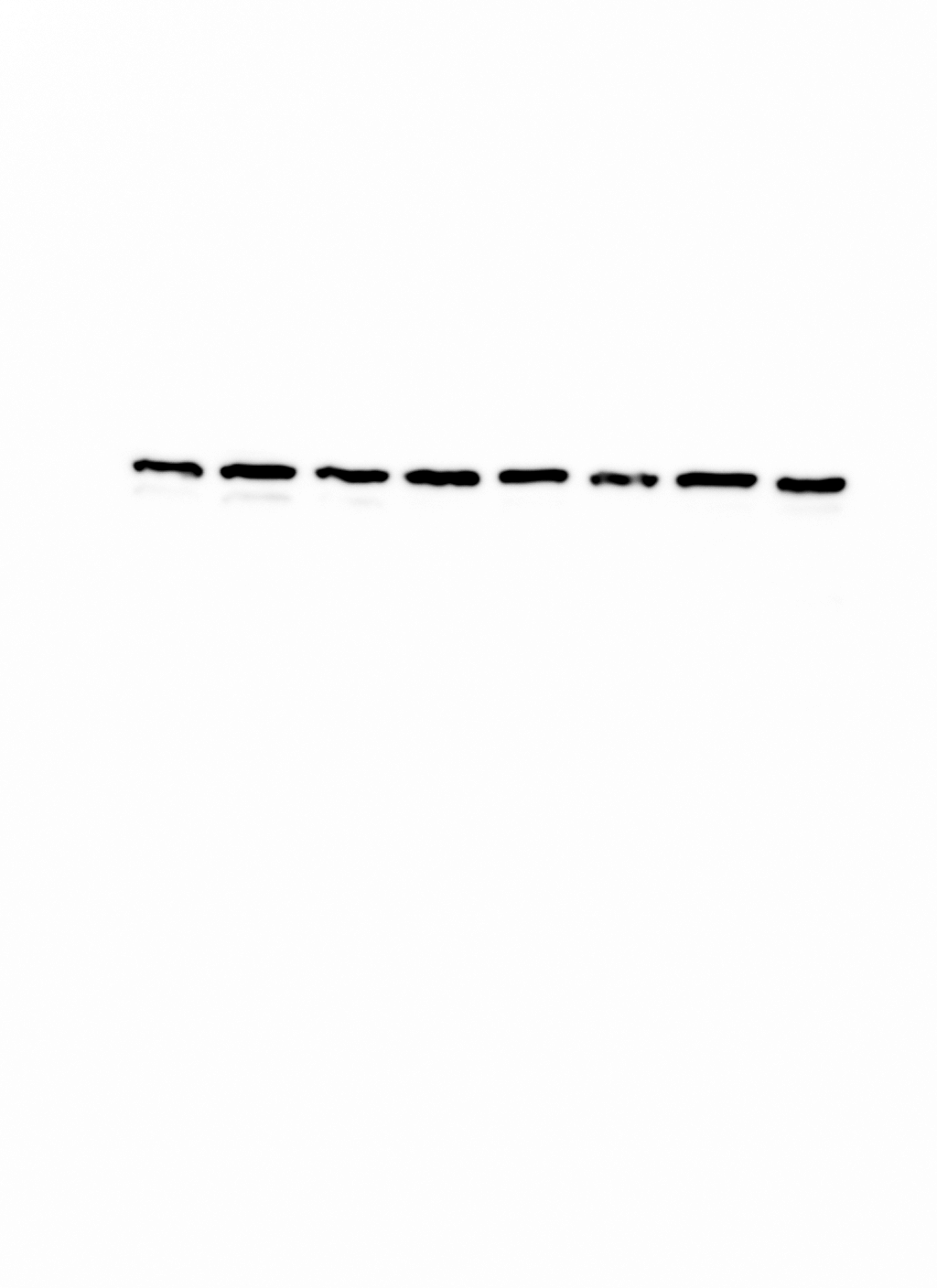

Supplement: Supplementary file 1 [file molecules-25-03667-s001.zip › molecules-891615 - proofread supplementary/Raw data of the Blots/Figure 2D/Striatum/28-Fig 2D-striatum_proBDNF-1.bmp]

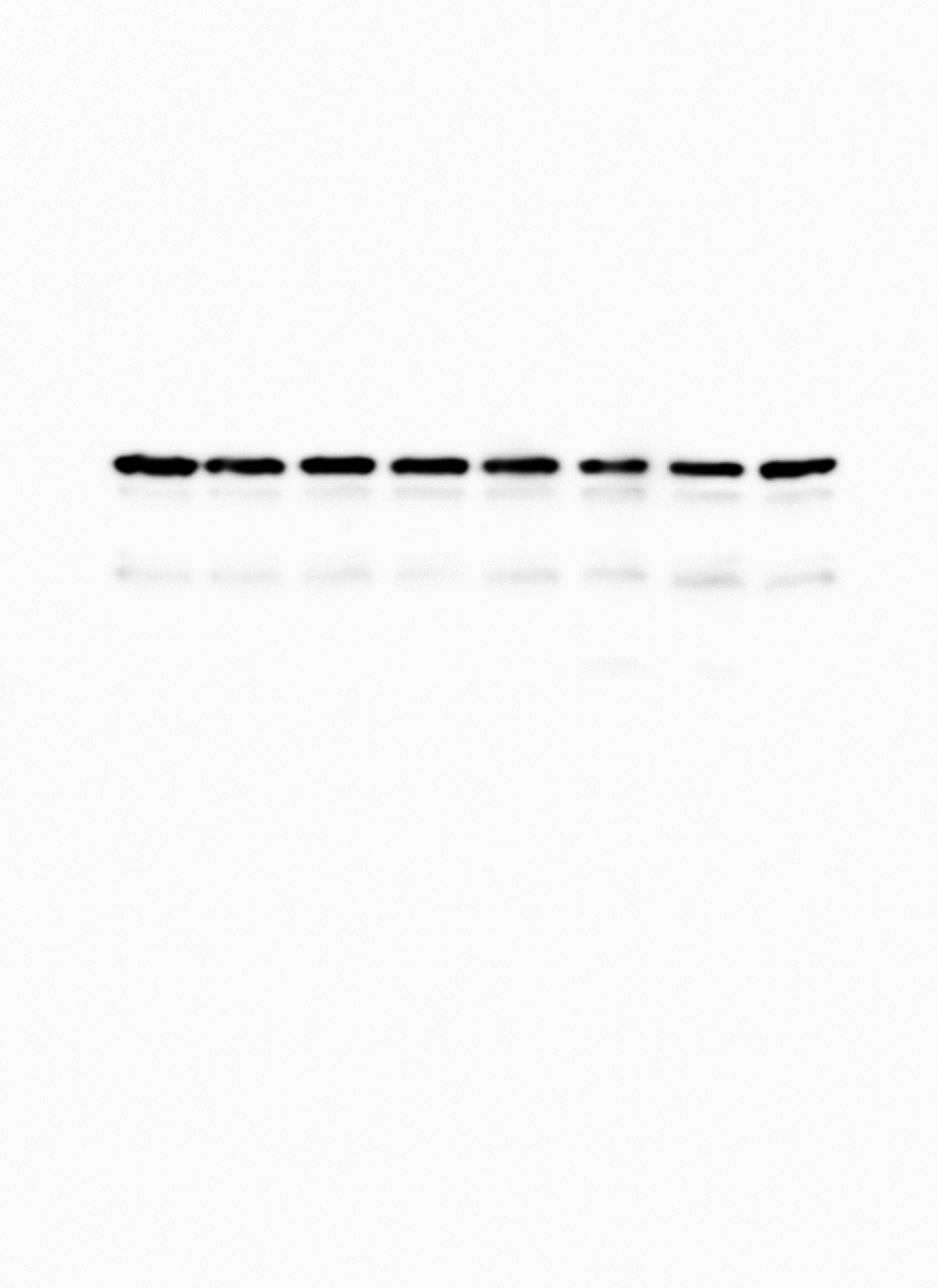

Supplement: Supplementary file 1 [file molecules-25-03667-s001.zip › molecules-891615 - proofread supplementary/Raw data of the Blots/Figure 2D/Striatum/29-Fig 2D-striatum_proBDNF-2.bmp]

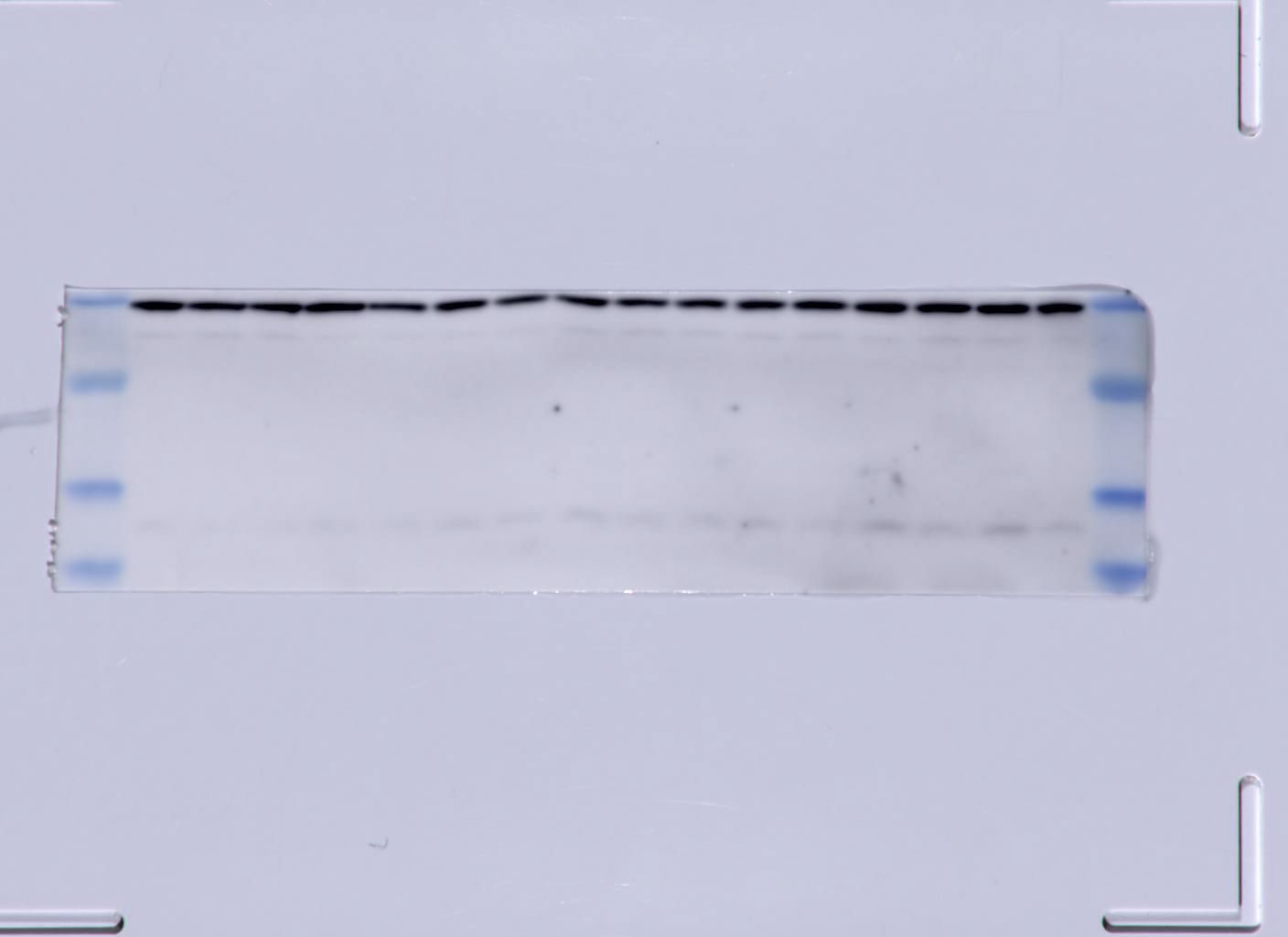

Supplement: Supplementary file 1 [file molecules-25-03667-s001.zip › molecules-891615 - proofread supplementary/Raw data of the Blots/Figure 2D/Striatum/30-Fig 2D-striatum_proBDNF-3.jpg]

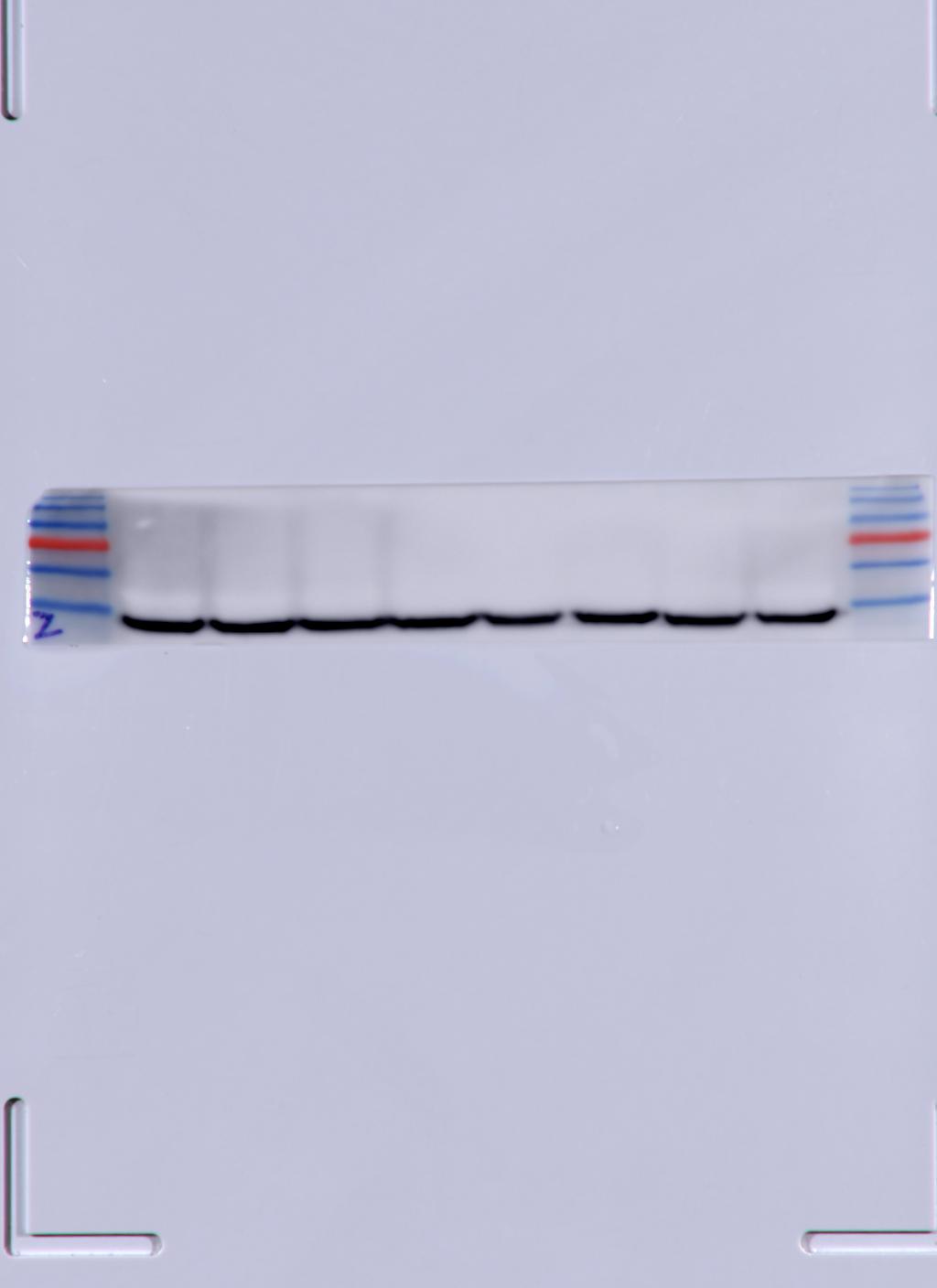

Supplement: Supplementary file 1 [file molecules-25-03667-s001.zip › molecules-891615 - proofread supplementary/Raw data of the Blots/Figure 2D/Striatum/31-Fig 2D-striatum_actin-1.jpg]

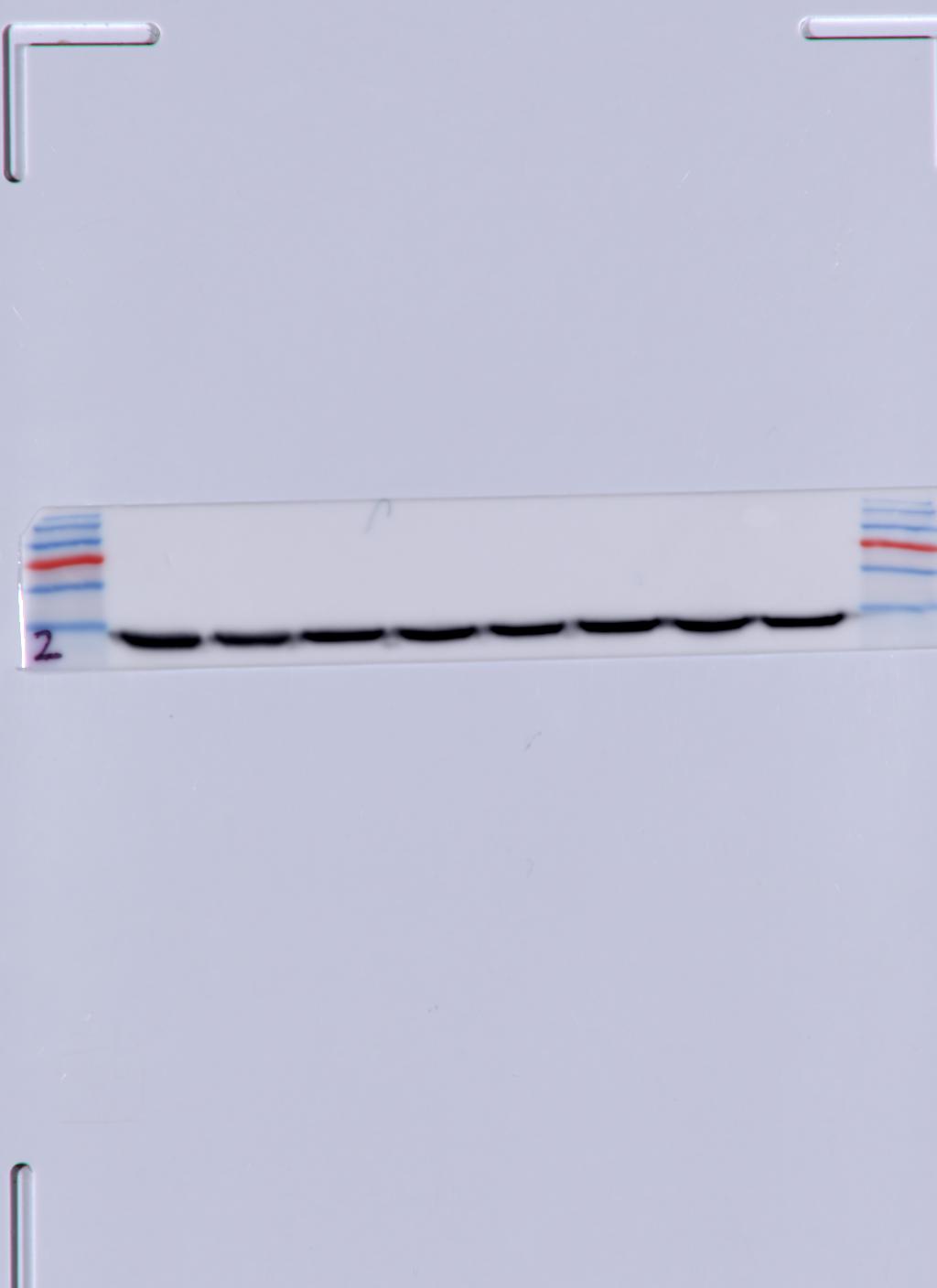

Supplement: Supplementary file 1 [file molecules-25-03667-s001.zip › molecules-891615 - proofread supplementary/Raw data of the Blots/Figure 2D/Striatum/32-Fig 2D-striatum_actin-2.jpg]

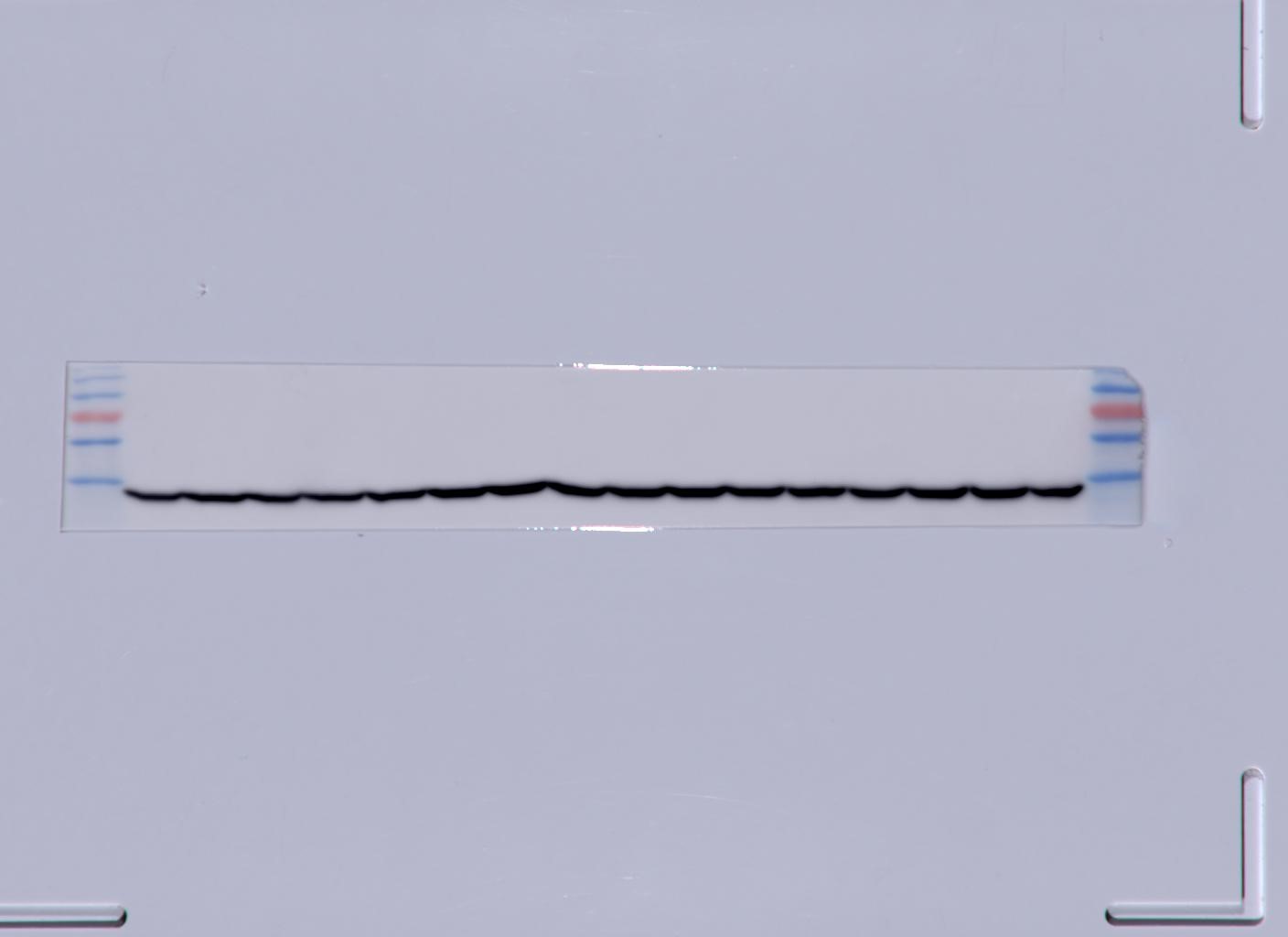

Supplement: Supplementary file 1 [file molecules-25-03667-s001.zip › molecules-891615 - proofread supplementary/Raw data of the Blots/Figure 2D/Striatum/33-Fig 2D-striatum_actin-3.jpg]

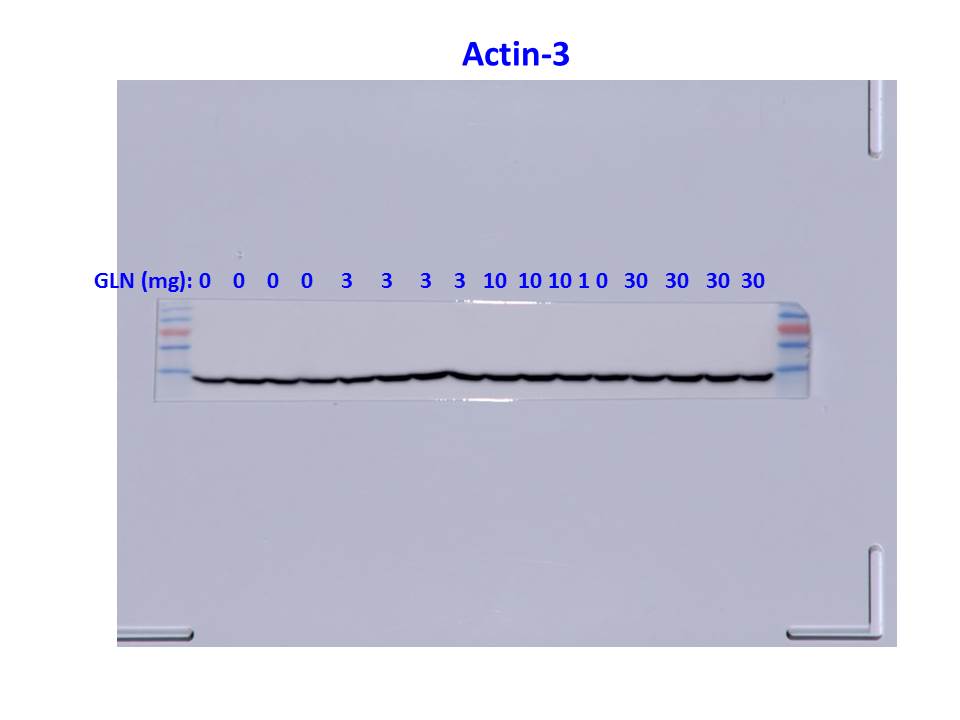

Supplement: Supplementary file 1 [file molecules-25-03667-s001.zip › molecules-891615 - proofread supplementary/Raw data of the Blots/Figure 2D/Striatum/34-Fig 2D-Description for 16 samples-actin-3.jpg]

# Actin-3

GLN (mg): 0 0 0 0 3 3 3 3 10 10 10 10 30 30 30 30

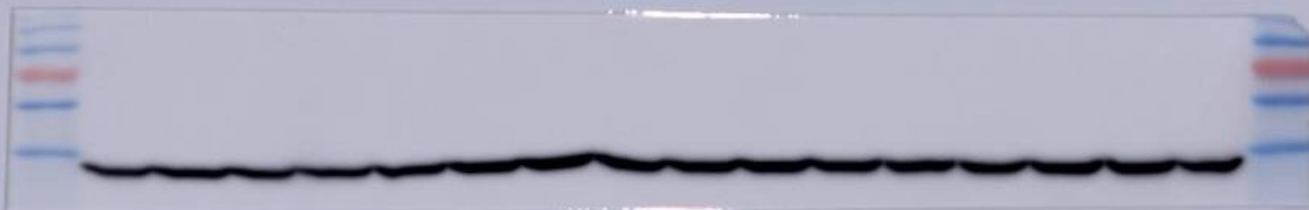

Supplement: Supplementary file 1 [file molecules-25-03667-s001.zip › molecules-891615 - proofread supplementary/Raw data of the Blots/Figure 2D/Striatum/Fig 2D-Description for 16 samples-actin-3.pdf]

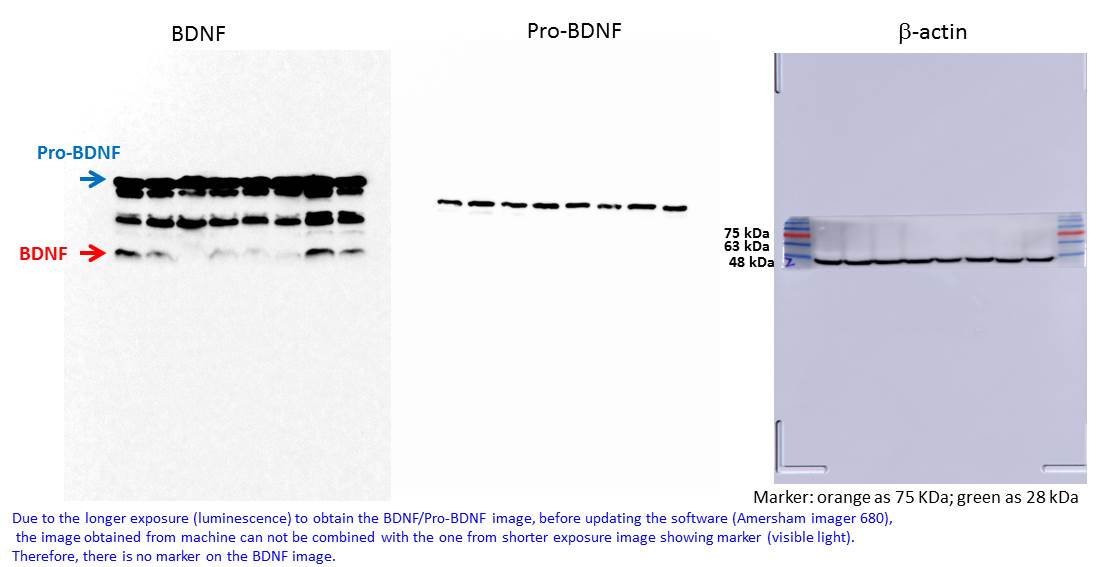

Supplement: Supplementary file 1 [file molecules-25-03667-s001.zip › molecules-891615 - proofread supplementary/Raw data of the Blots/Figure 2D/Striatum/Fig 2D-Striatum description.jpg]

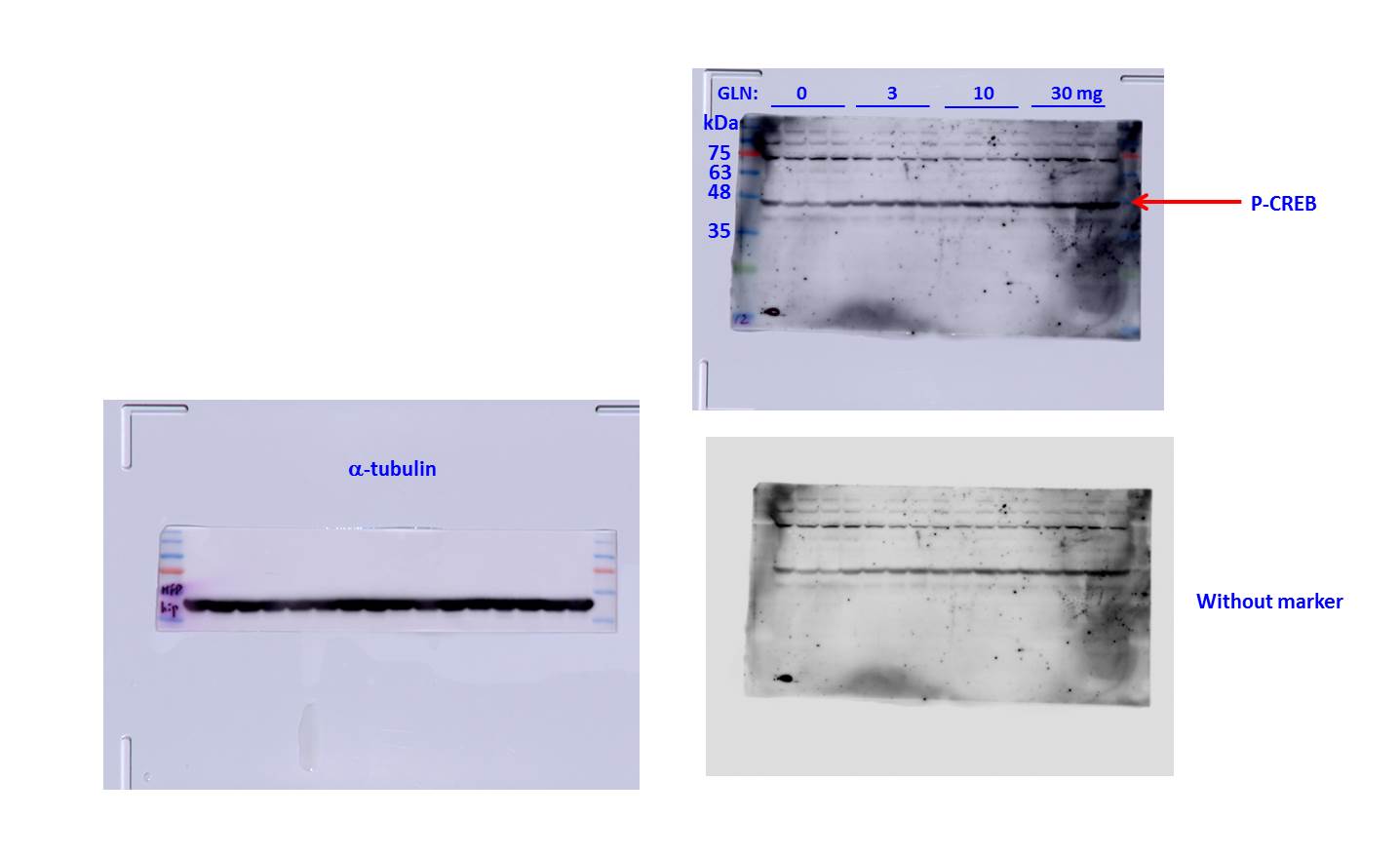

Supplement: Supplementary file 1 [file molecules-25-03667-s001.zip › molecules-891615 - proofread supplementary/Raw data of the Blots/Figure 3C/46-Fig 3C-Description for Figure 3C.jpg]

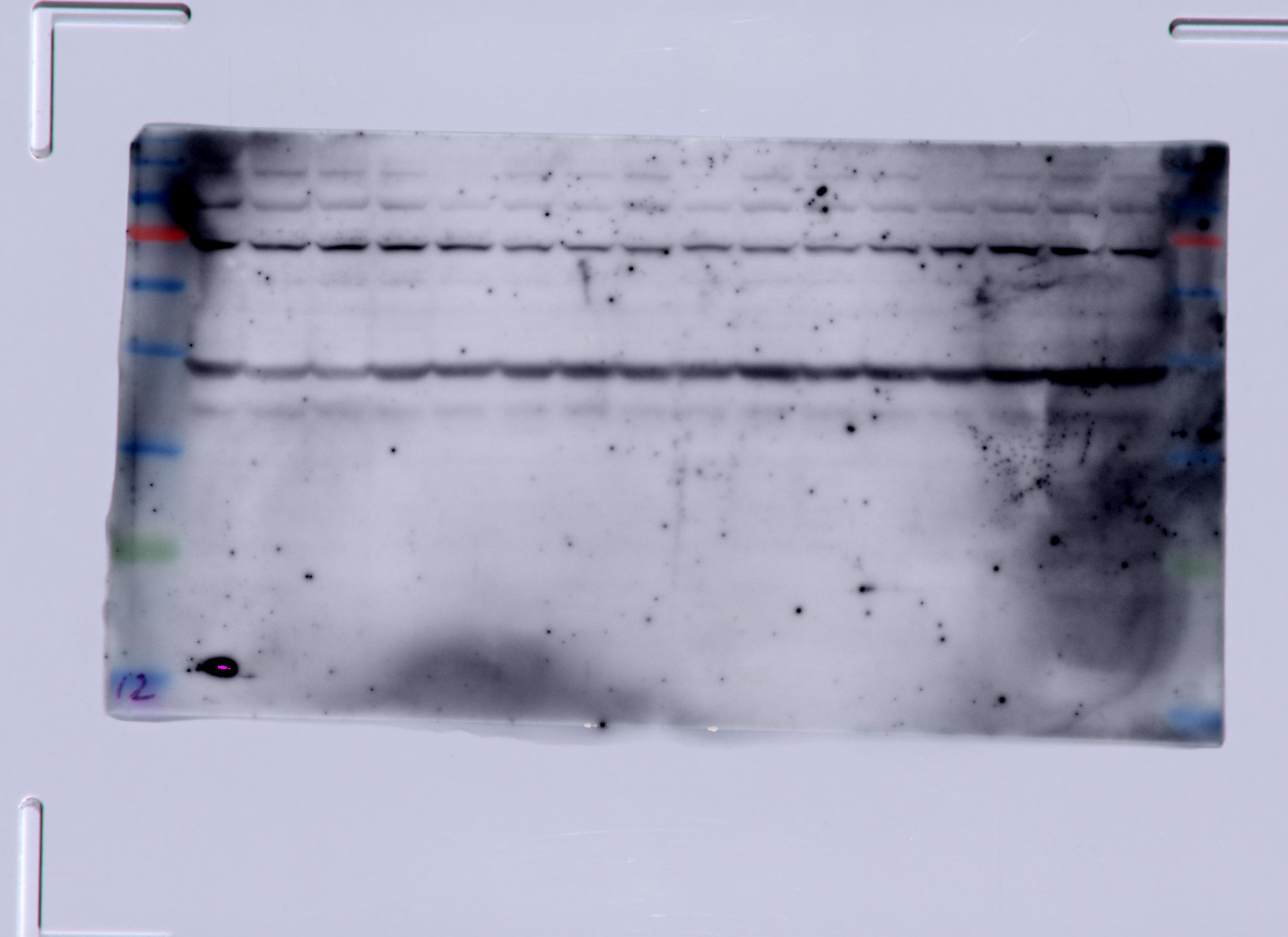

Supplement: Supplementary file 1 [file molecules-25-03667-s001.zip › molecules-891615 - proofread supplementary/Raw data of the Blots/Figure 3C/47-Fig 3C-phospho-CREB+Marker.jpg]

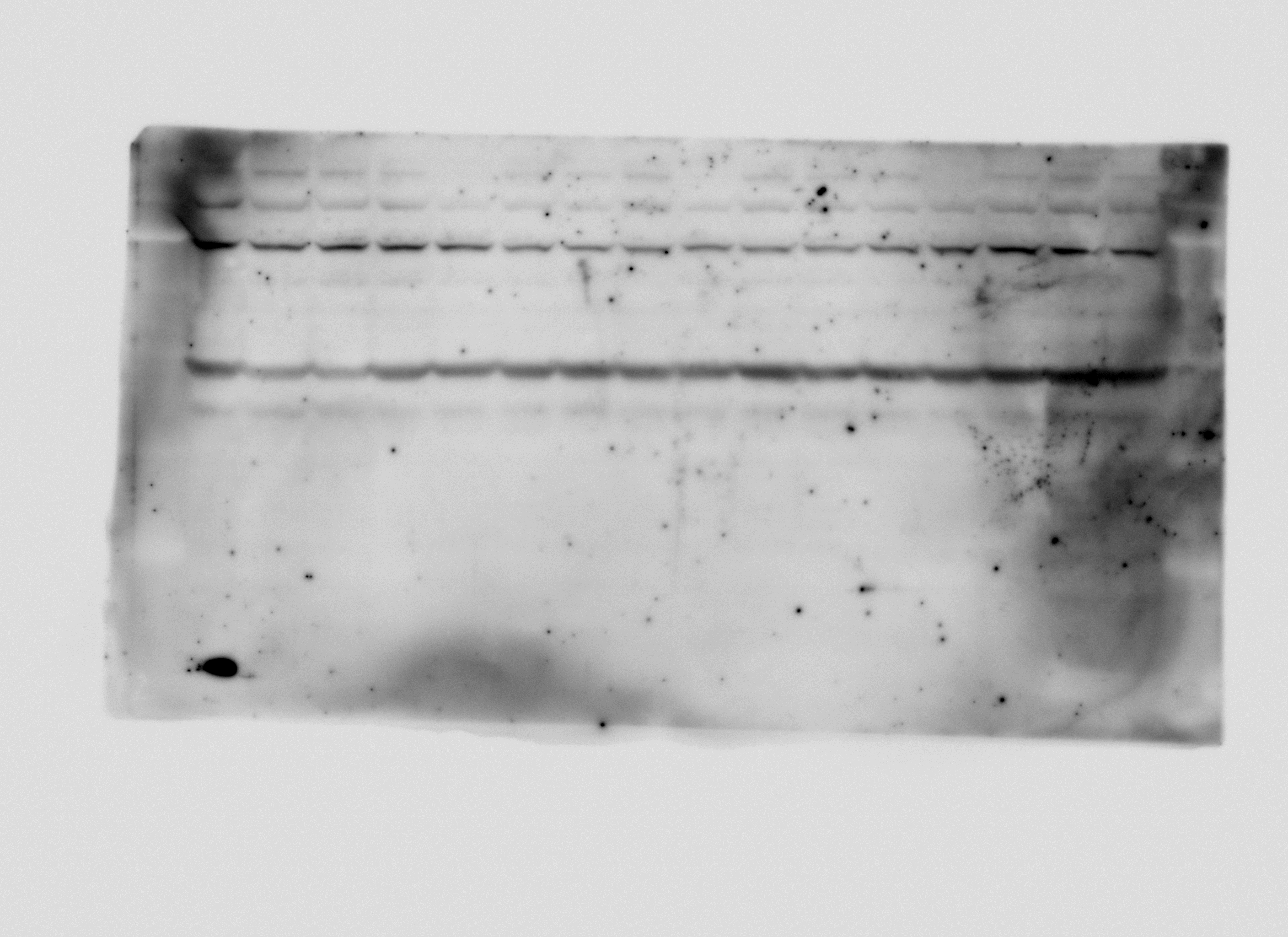

Supplement: Supplementary file 1 [file molecules-25-03667-s001.zip › molecules-891615 - proofread supplementary/Raw data of the Blots/Figure 3C/48-Fig 3C-phospho-CREB.bmp]

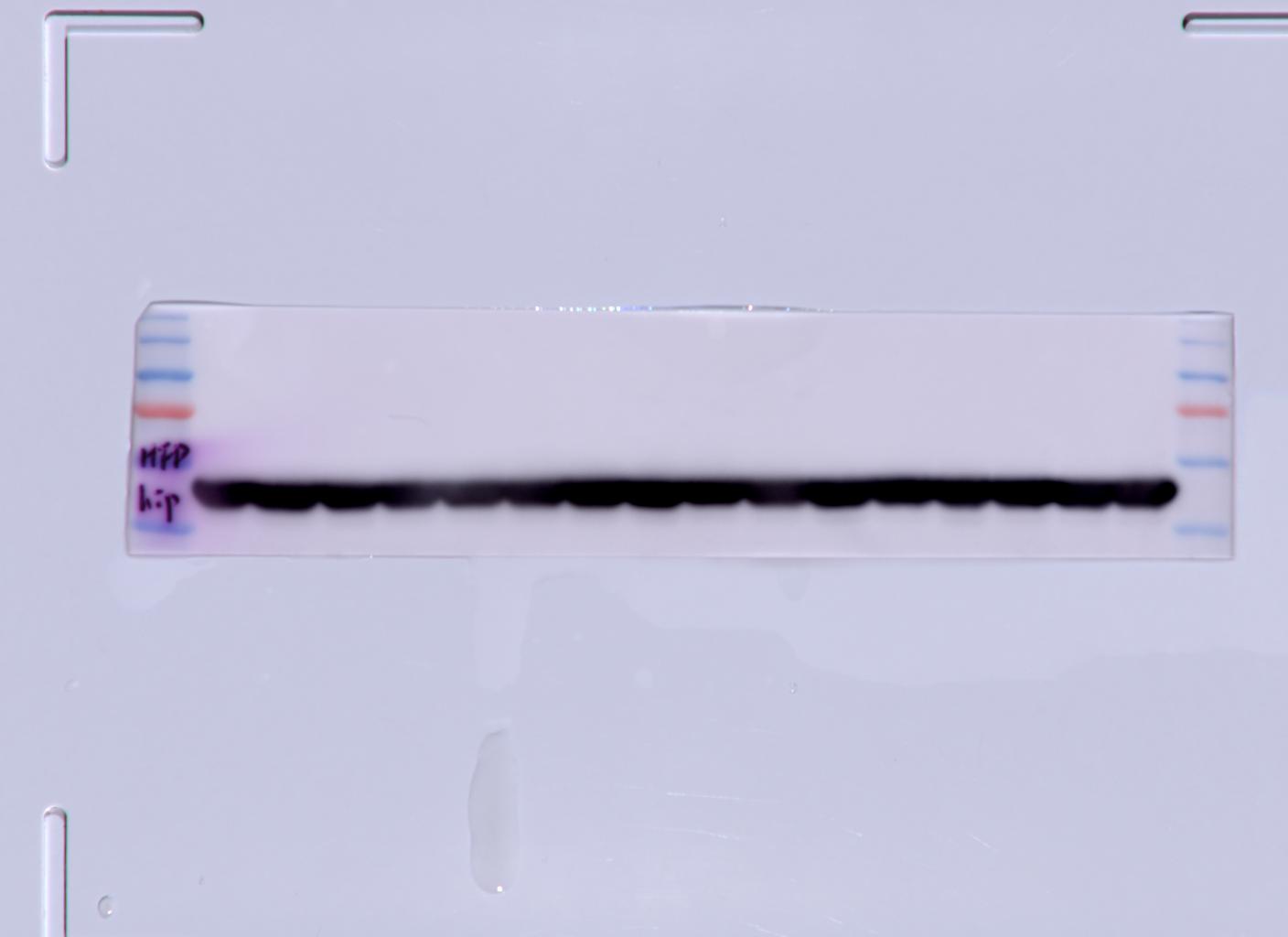

Supplement: Supplementary file 1 [file molecules-25-03667-s001.zip › molecules-891615 - proofread supplementary/Raw data of the Blots/Figure 3C/49-Fig 3C-a-tubulin.jpg]

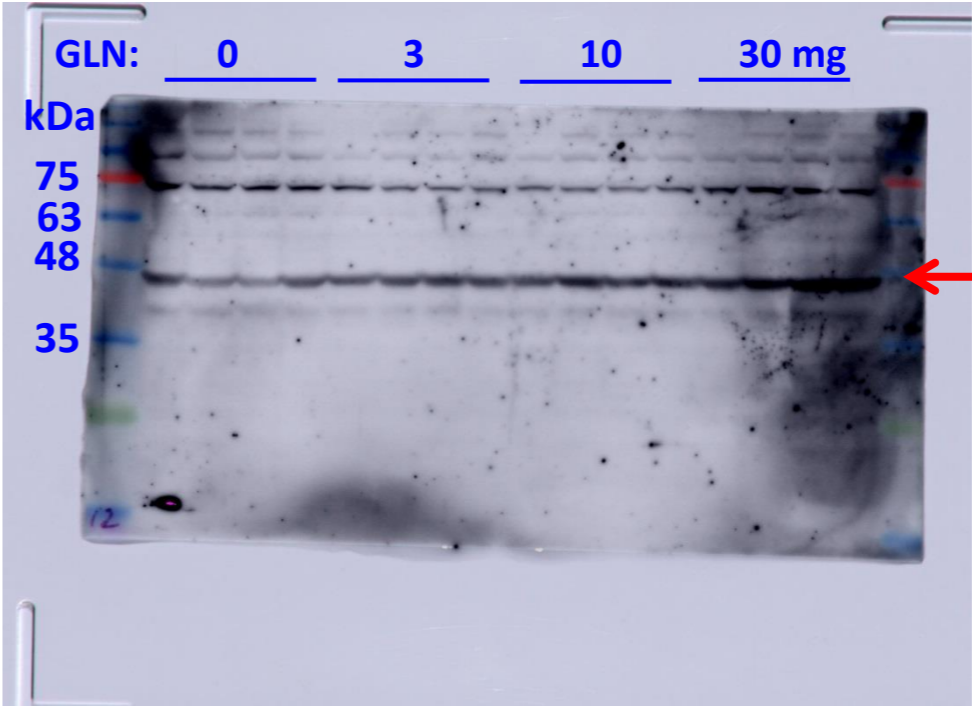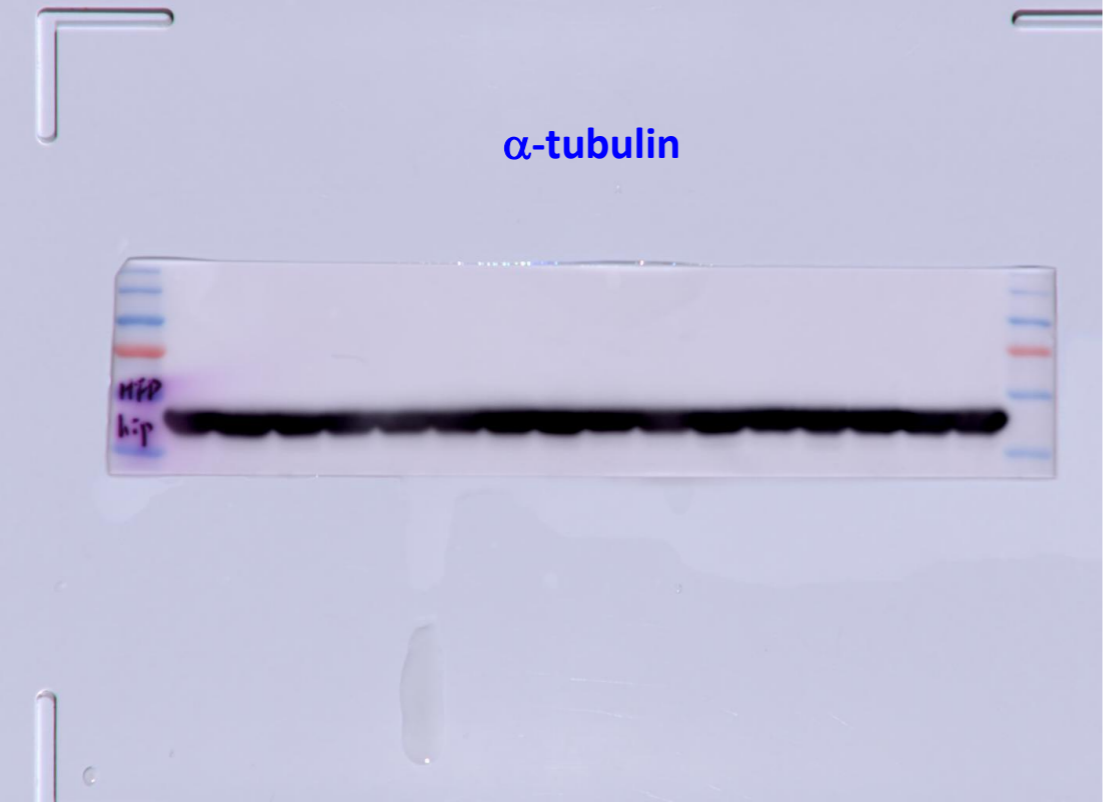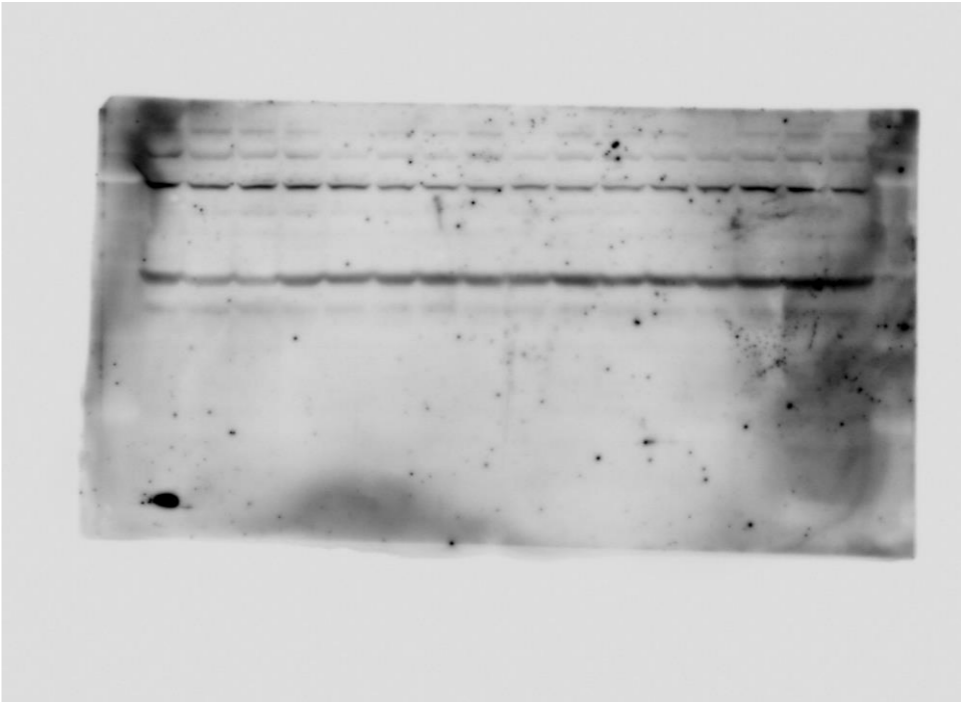

Supplement: Supplementary file 1 [file molecules-25-03667-s001.zip › molecules-891615 - proofread supplementary/Raw data of the Blots/Figure 3C/Fig 3C-Description for Figure 3C.pdf]

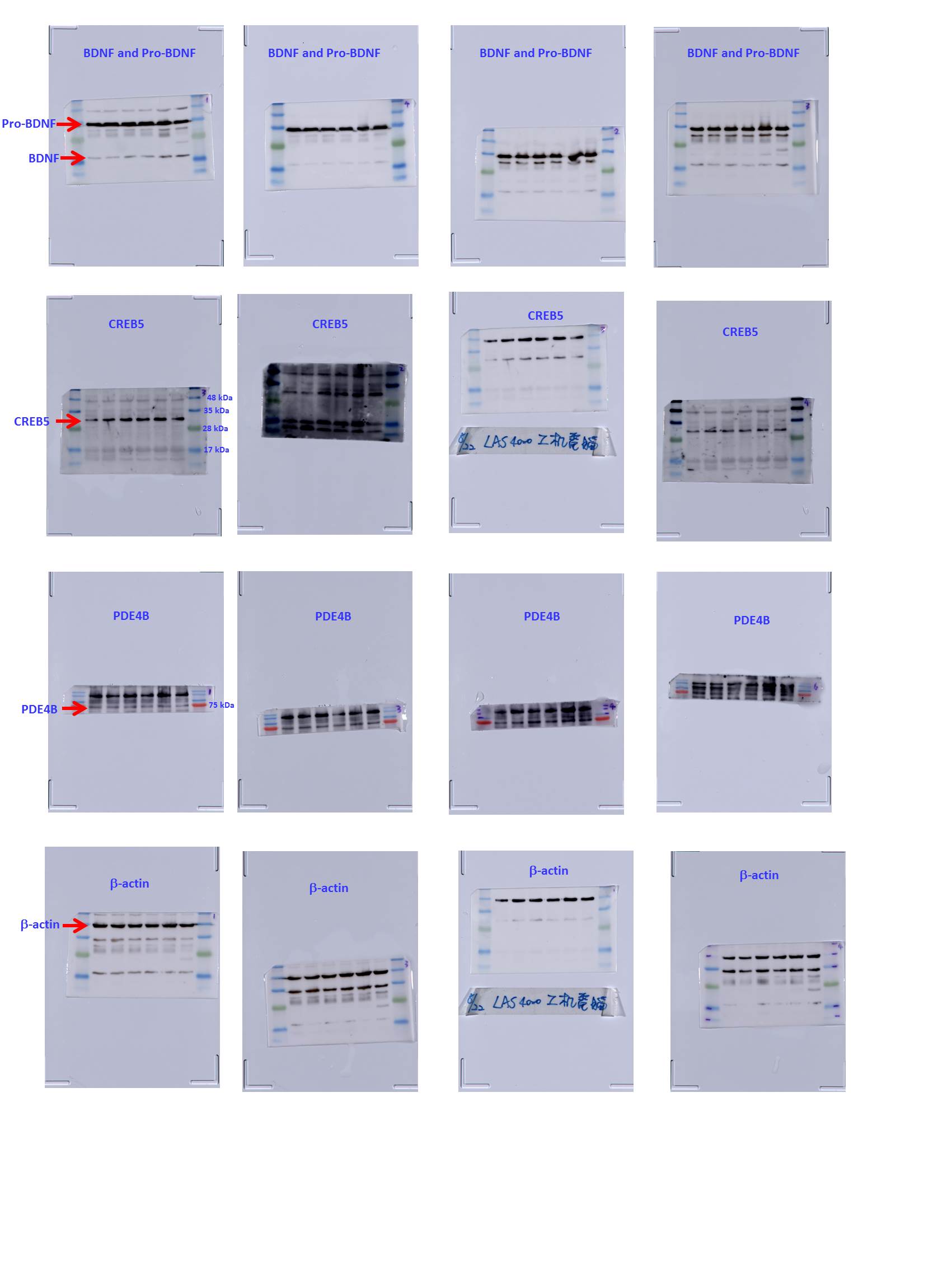

Supplement: Supplementary file 1 [file molecules-25-03667-s001.zip › molecules-891615 - proofread supplementary/Raw data of the Blots/Figure 3E/50-Fig 3E-Description for Figue 3E.jpg]

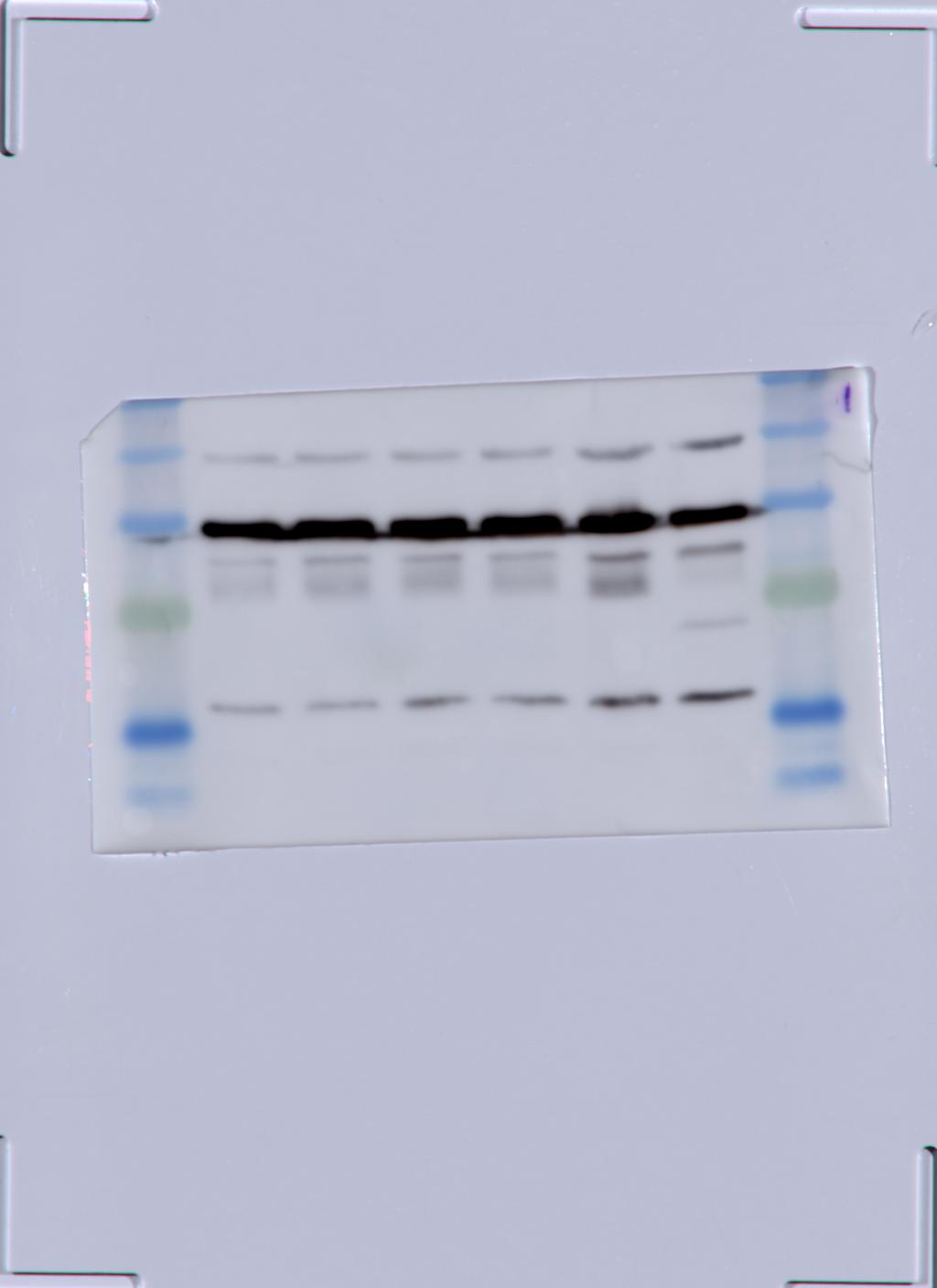

Supplement: Supplementary file 1 [file molecules-25-03667-s001.zip › molecules-891615 - proofread supplementary/Raw data of the Blots/Figure 3E/51-Fig 3E-BDNF and Pro-BDNF-1.jpg]

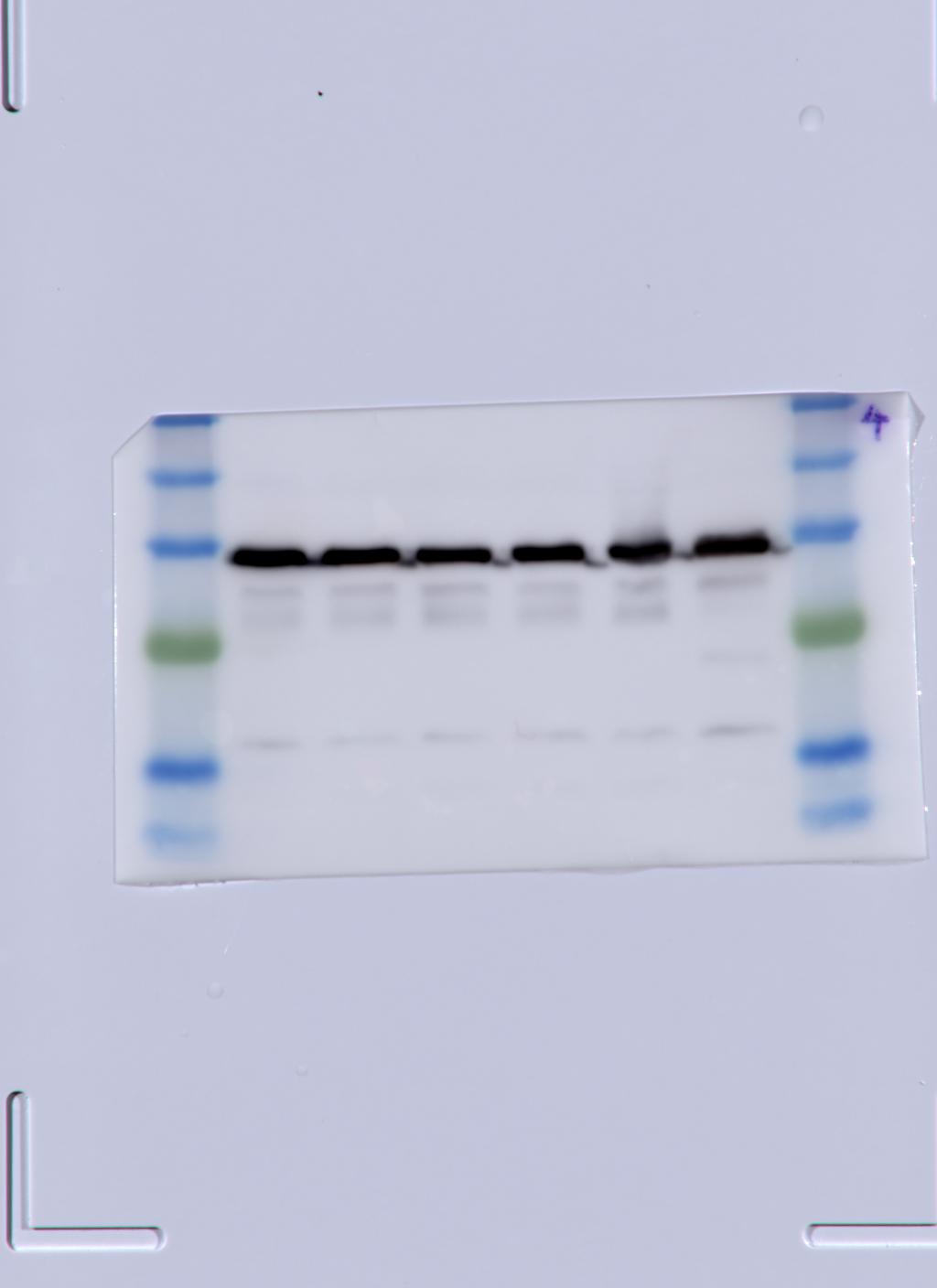

Supplement: Supplementary file 1 [file molecules-25-03667-s001.zip › molecules-891615 - proofread supplementary/Raw data of the Blots/Figure 3E/52-Fig 3E-BDNF and Pro-BDNF-2.jpg]

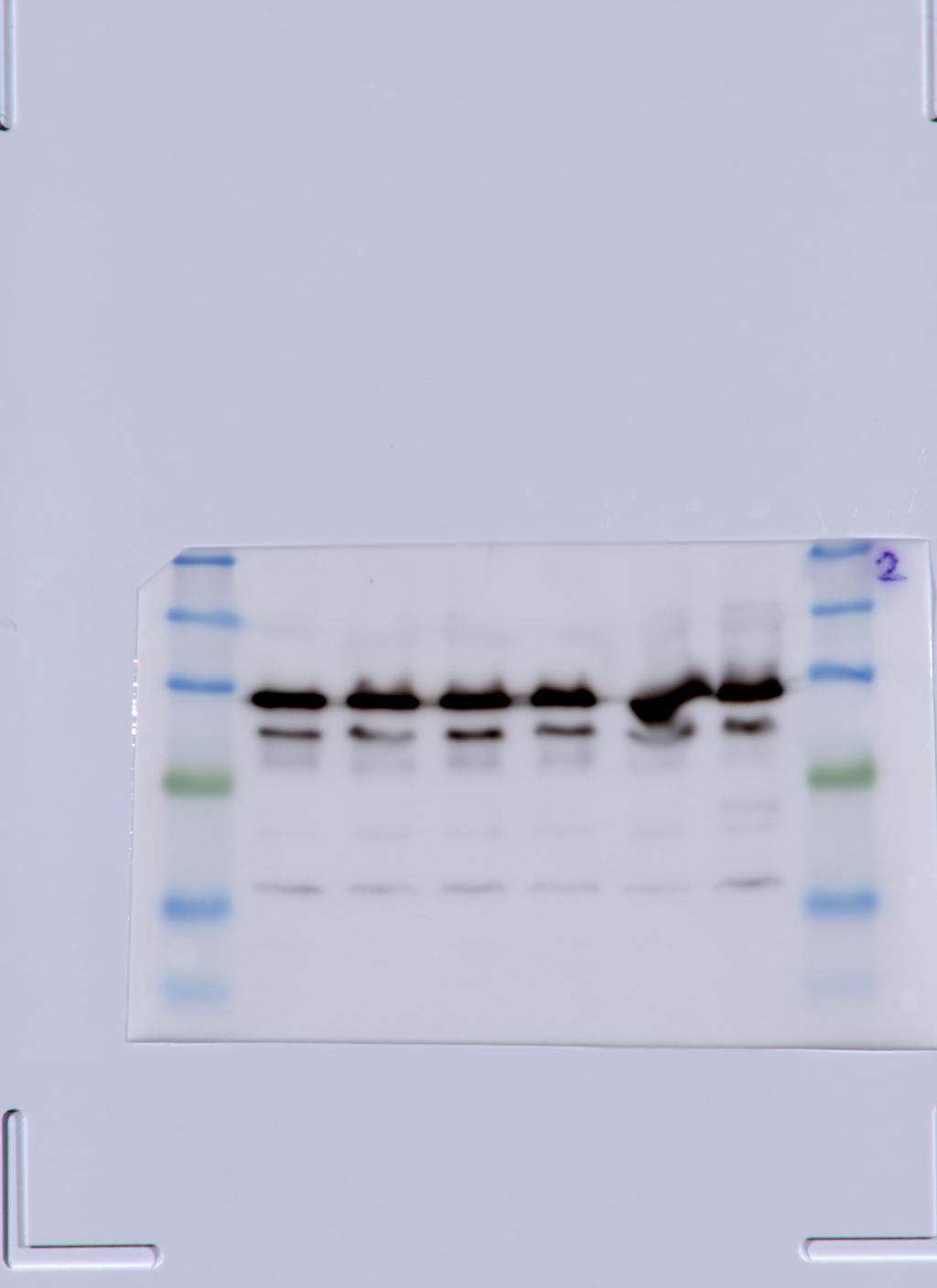

Supplement: Supplementary file 1 [file molecules-25-03667-s001.zip › molecules-891615 - proofread supplementary/Raw data of the Blots/Figure 3E/53-Fig 3E-BDNF and Pro-BDNF-3.jpg]

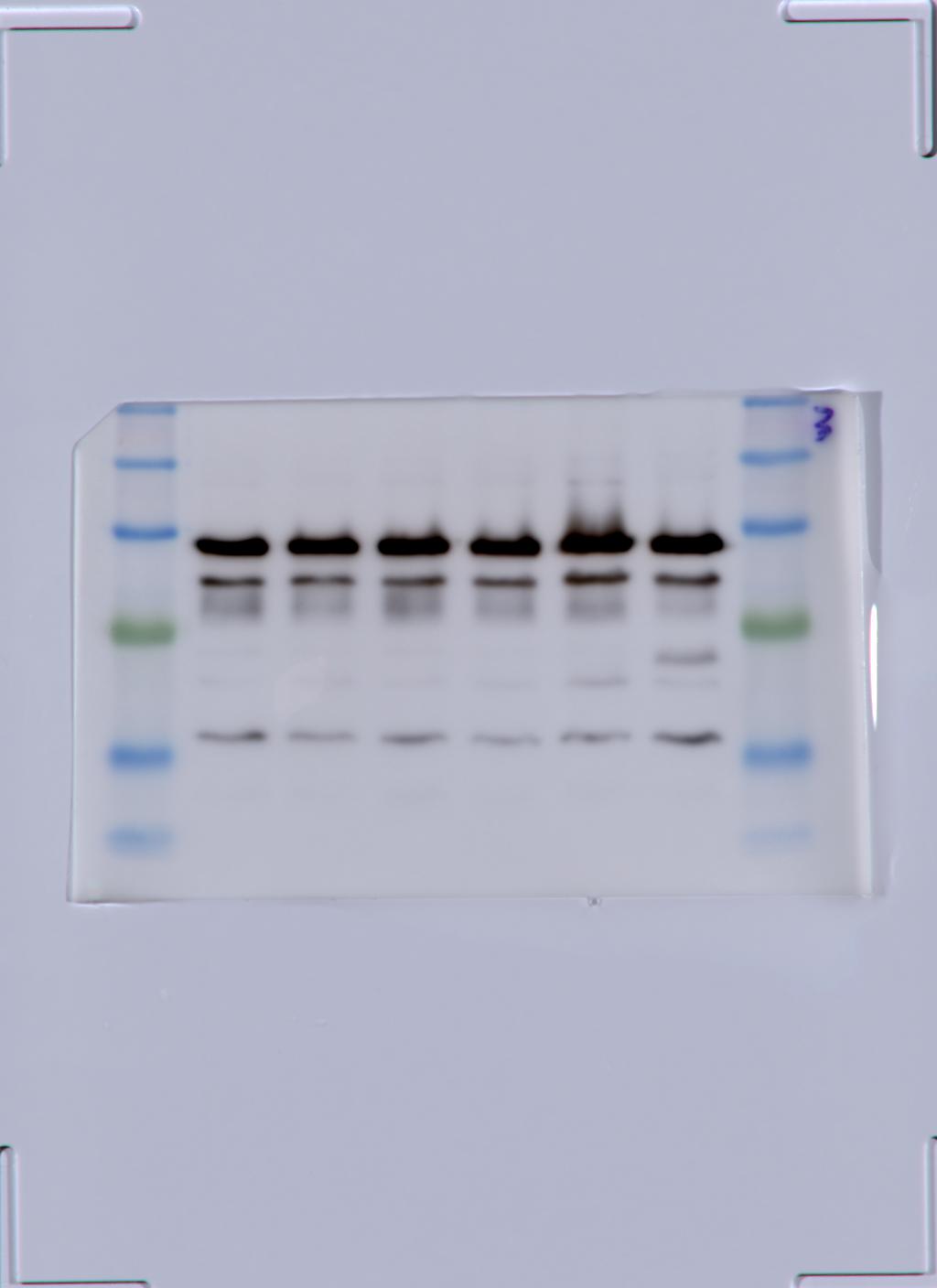

Supplement: Supplementary file 1 [file molecules-25-03667-s001.zip › molecules-891615 - proofread supplementary/Raw data of the Blots/Figure 3E/54-Fig 3E-BDNF and Pro-BDNF-4.jpg]

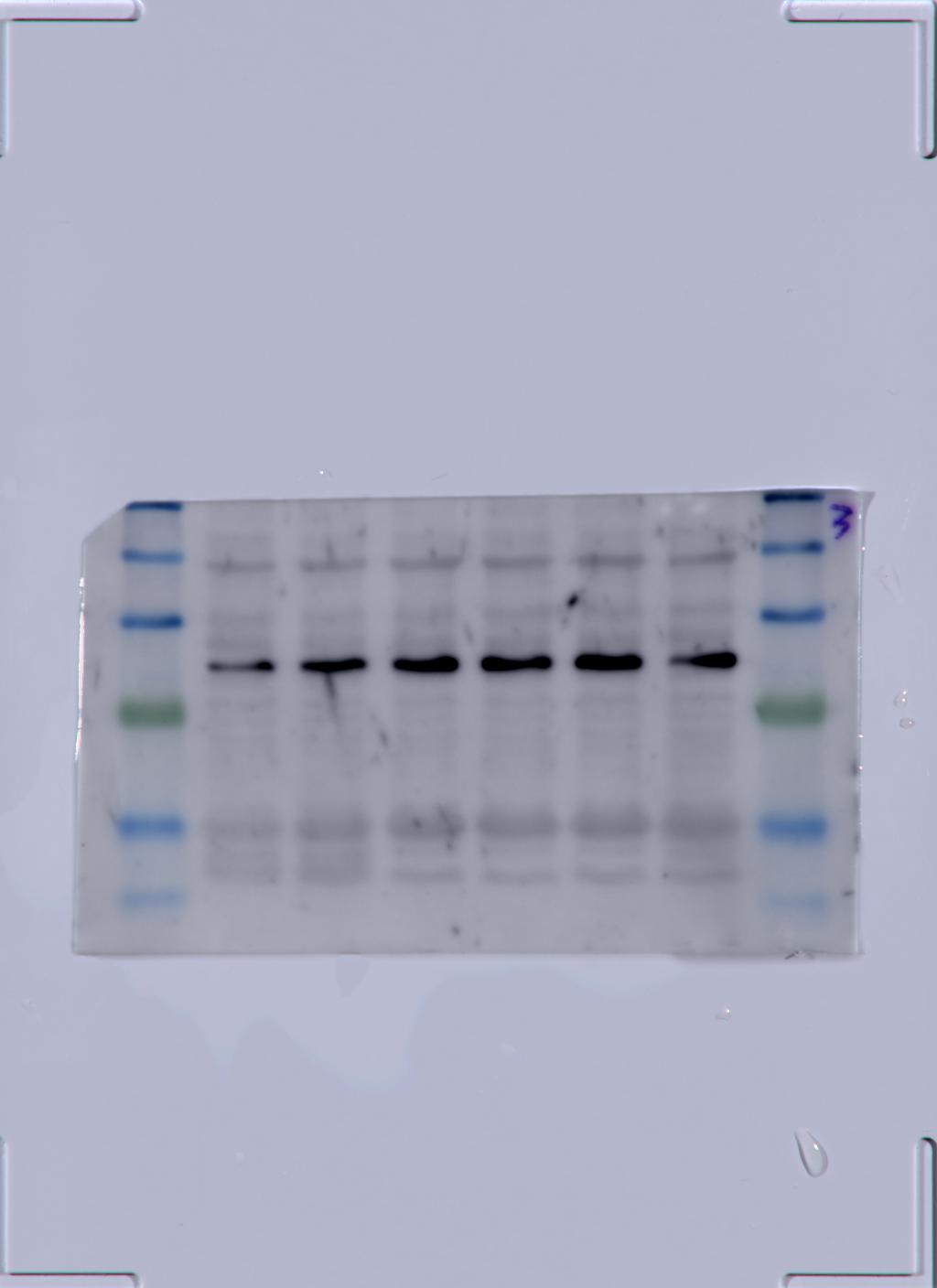

Supplement: Supplementary file 1 [file molecules-25-03667-s001.zip › molecules-891615 - proofread supplementary/Raw data of the Blots/Figure 3E/55-Fig 3E-CREB5-1.jpg]

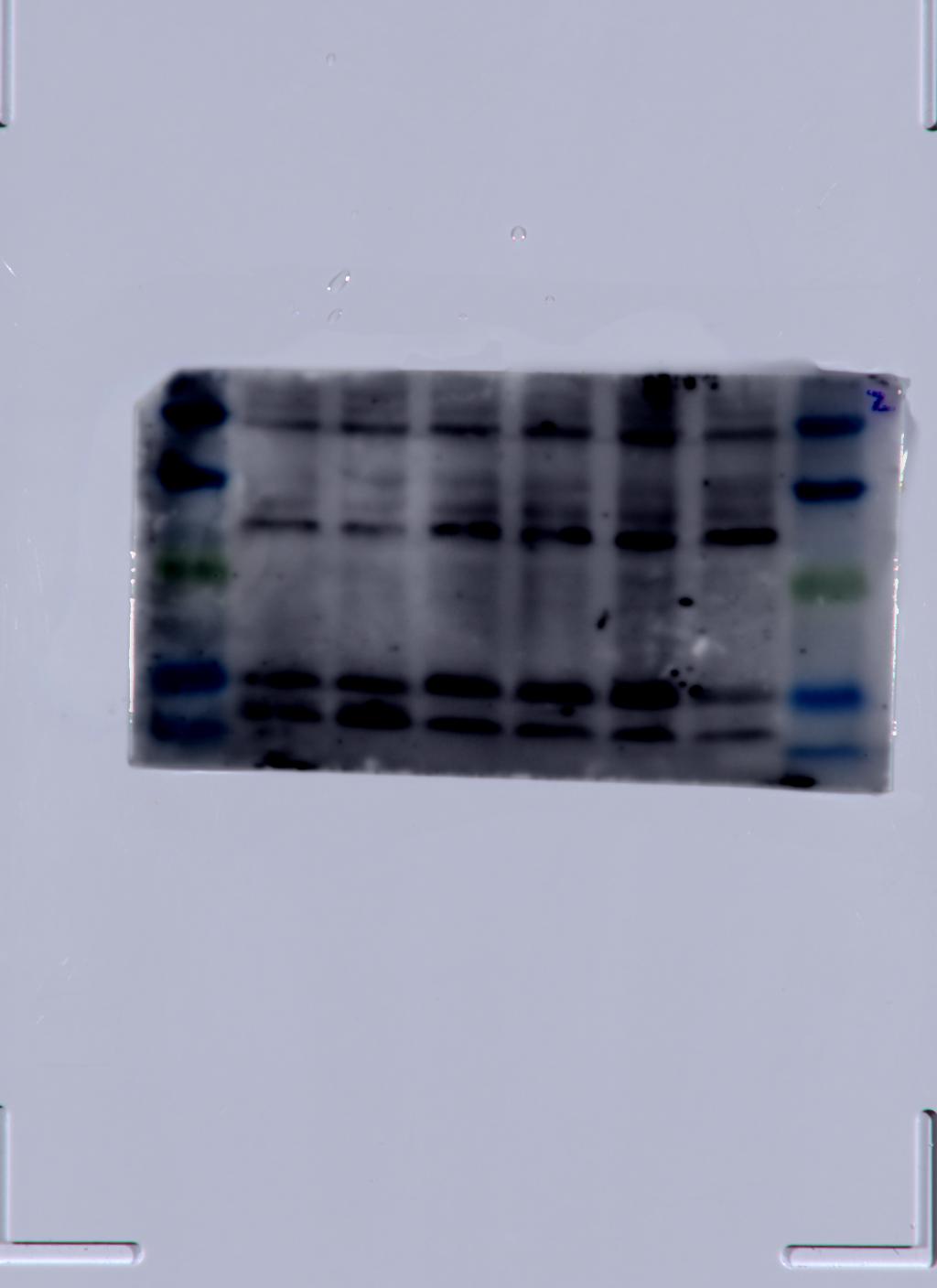

Supplement: Supplementary file 1 [file molecules-25-03667-s001.zip › molecules-891615 - proofread supplementary/Raw data of the Blots/Figure 3E/56-Fig 3E-CREB5-2.jpg]

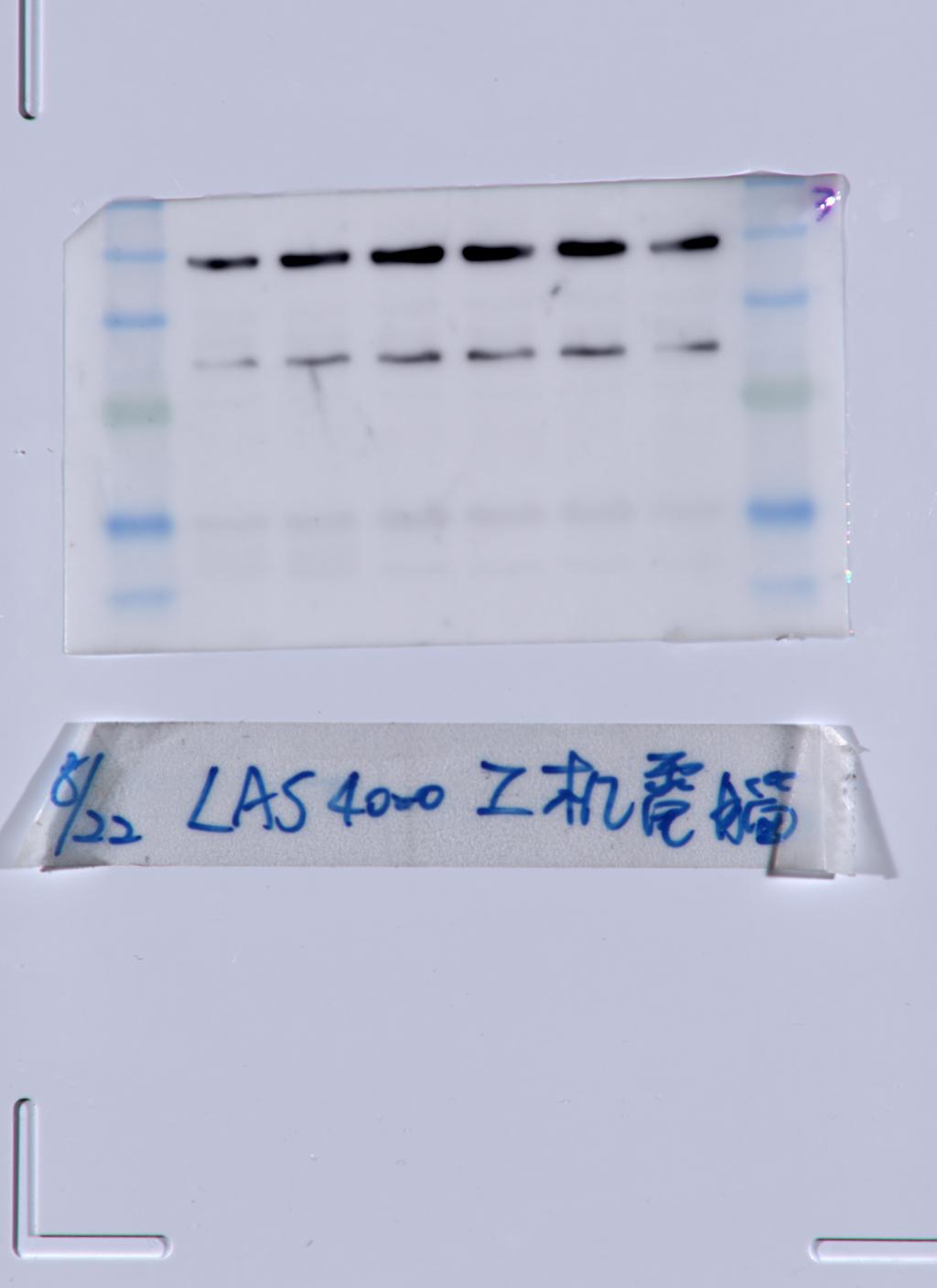

Supplement: Supplementary file 1 [file molecules-25-03667-s001.zip › molecules-891615 - proofread supplementary/Raw data of the Blots/Figure 3E/57-Fig 3E-CREB5-3.jpg]

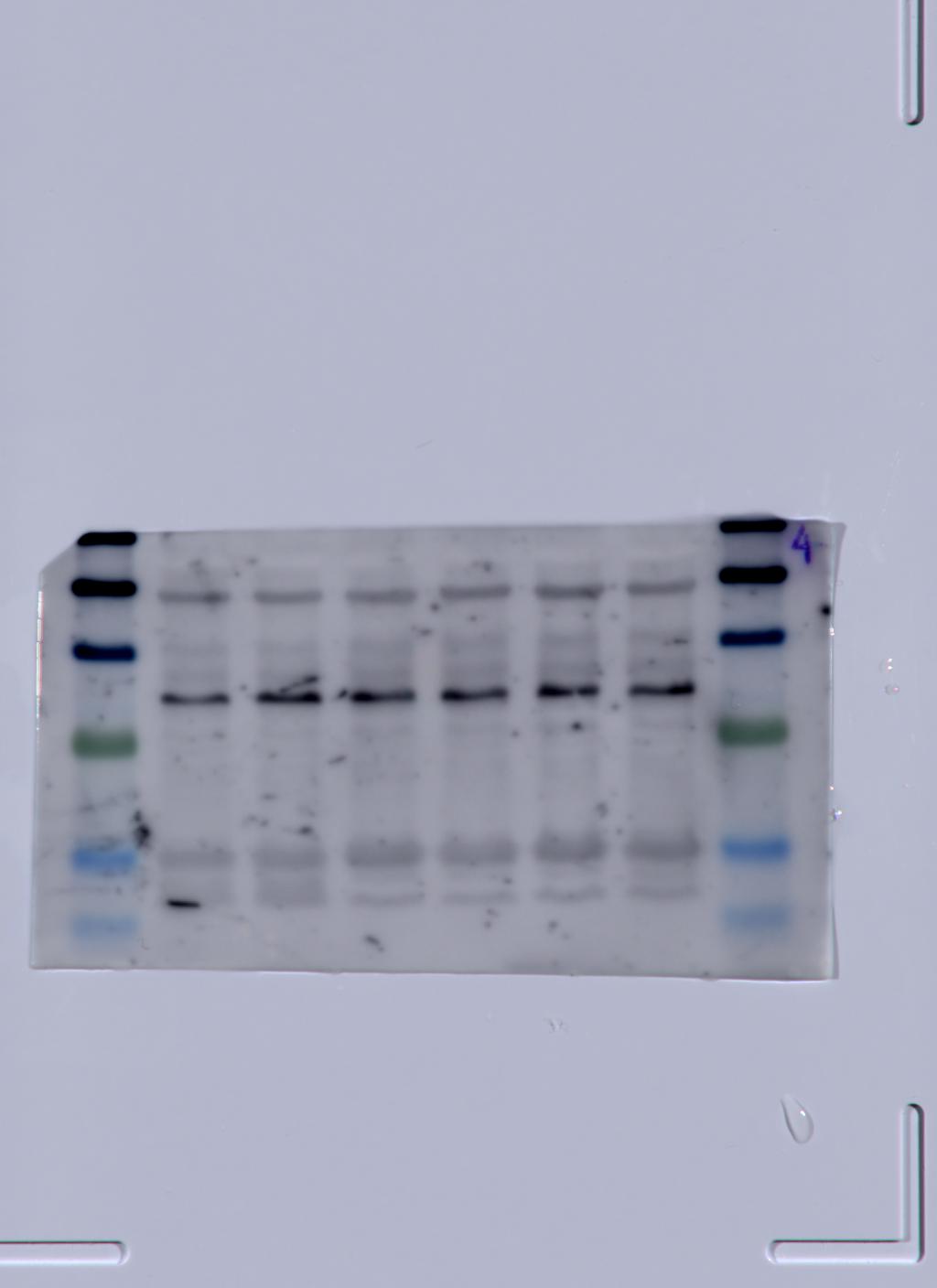

Supplement: Supplementary file 1 [file molecules-25-03667-s001.zip › molecules-891615 - proofread supplementary/Raw data of the Blots/Figure 3E/58-Fig 3E-CREB5-4.jpg]

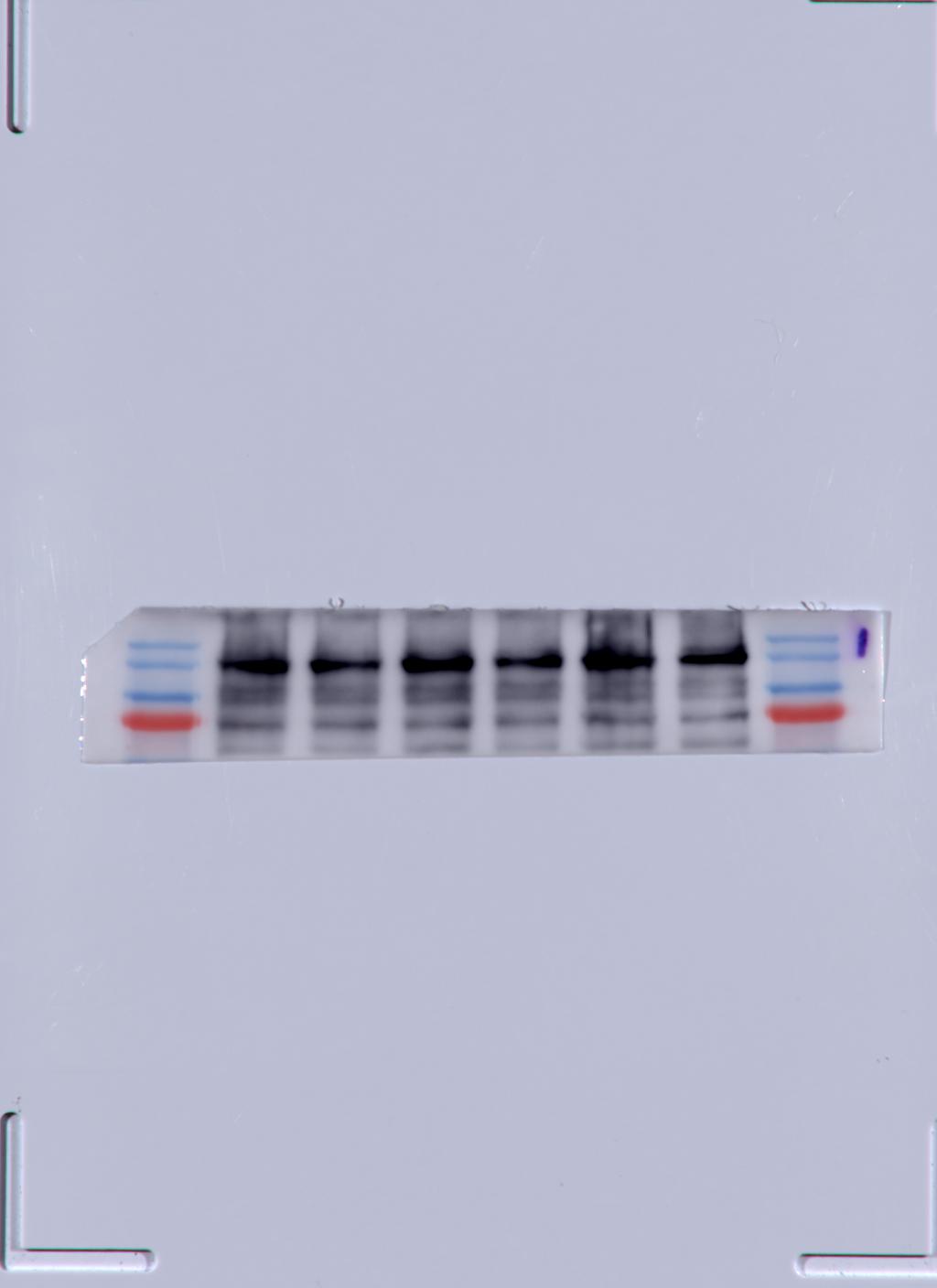

Supplement: Supplementary file 1 [file molecules-25-03667-s001.zip › molecules-891615 - proofread supplementary/Raw data of the Blots/Figure 3E/59-Fig 3E-PDE4B-1.jpg]

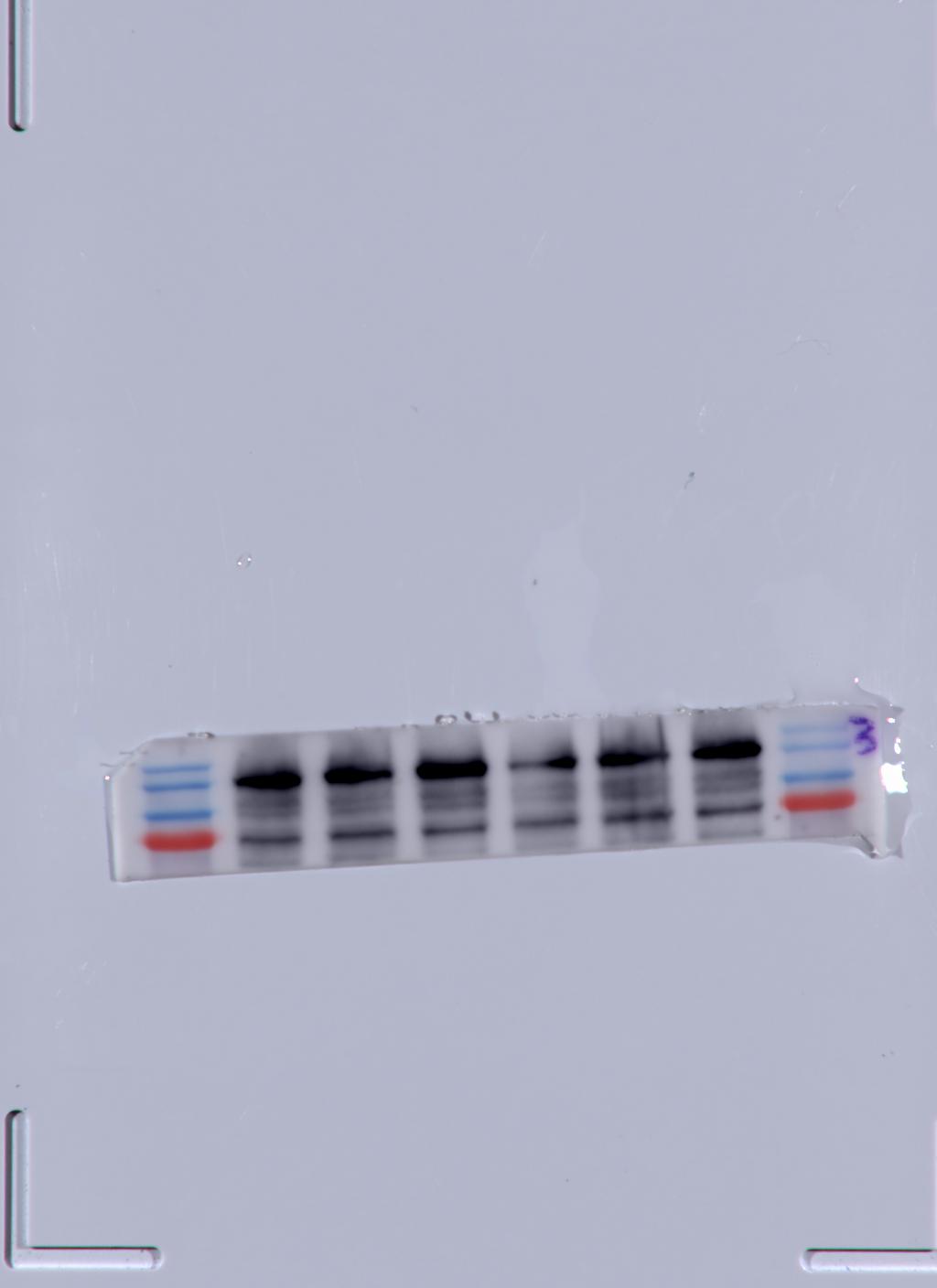

Supplement: Supplementary file 1 [file molecules-25-03667-s001.zip › molecules-891615 - proofread supplementary/Raw data of the Blots/Figure 3E/60-Fig 3E-PDE4B-2.jpg]

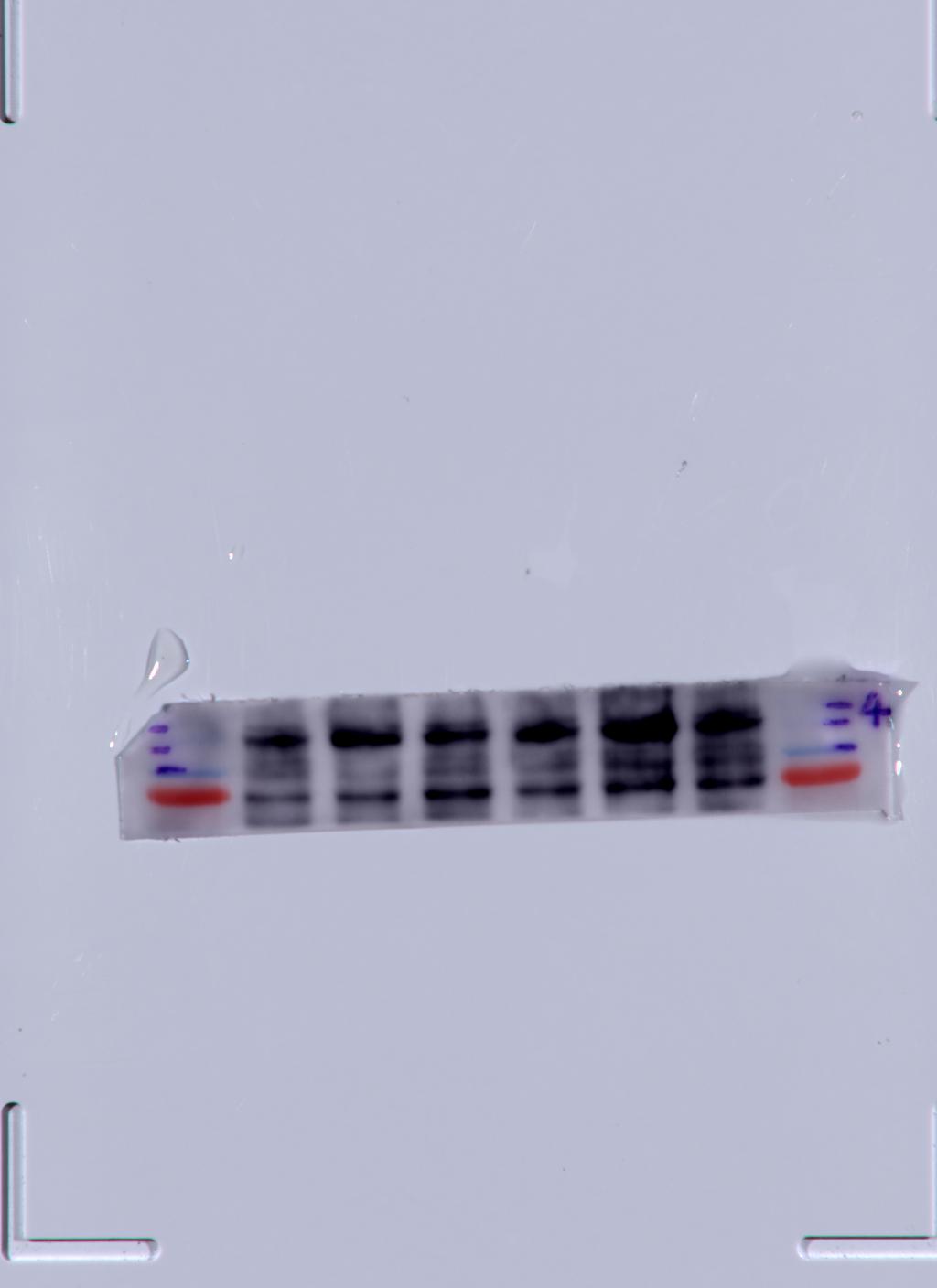

Supplement: Supplementary file 1 [file molecules-25-03667-s001.zip › molecules-891615 - proofread supplementary/Raw data of the Blots/Figure 3E/61-Fig 3E-PDE4B-3.jpg]

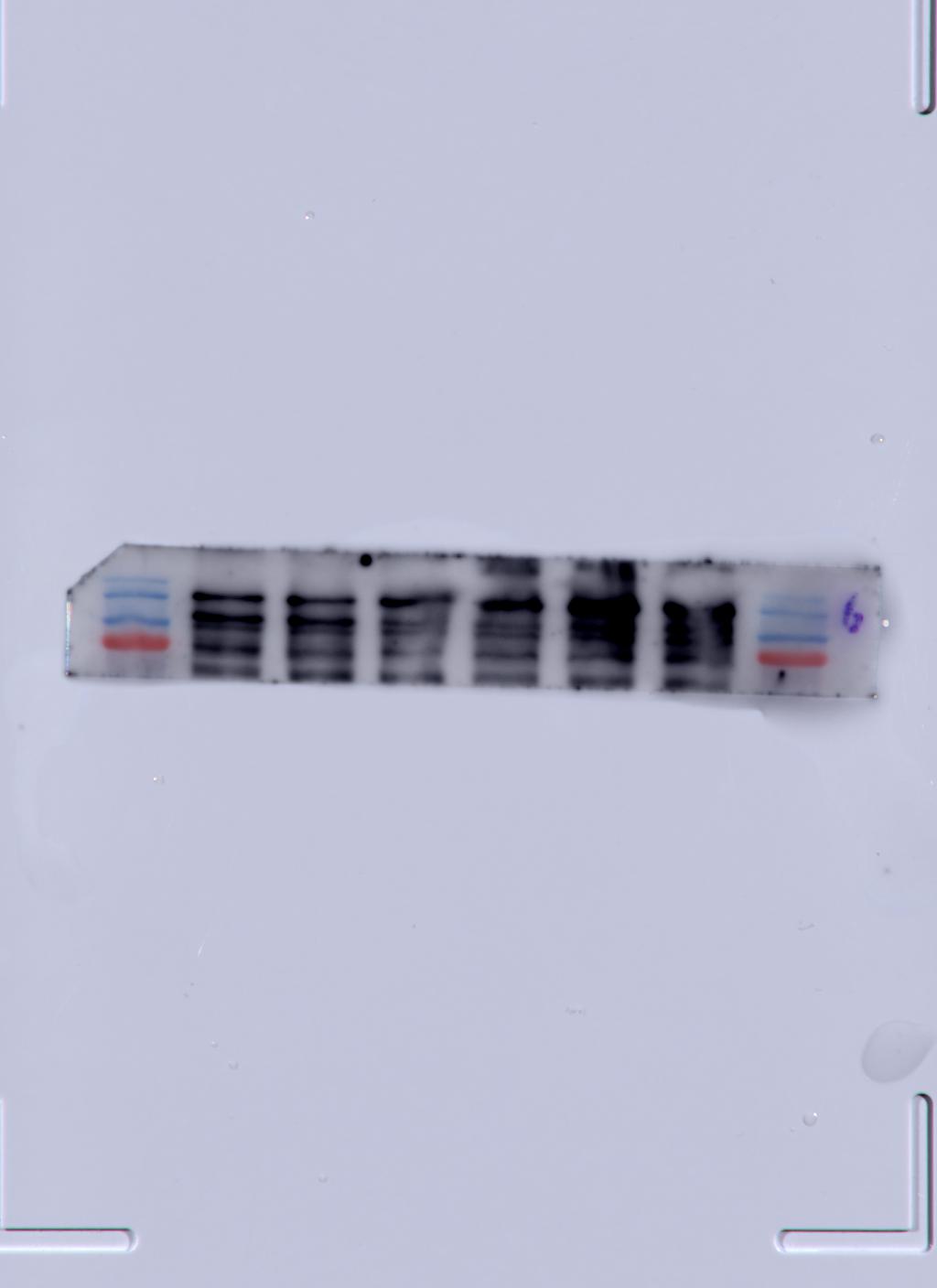

Supplement: Supplementary file 1 [file molecules-25-03667-s001.zip › molecules-891615 - proofread supplementary/Raw data of the Blots/Figure 3E/62-Fig 3E-PDE4B-4.jpg]

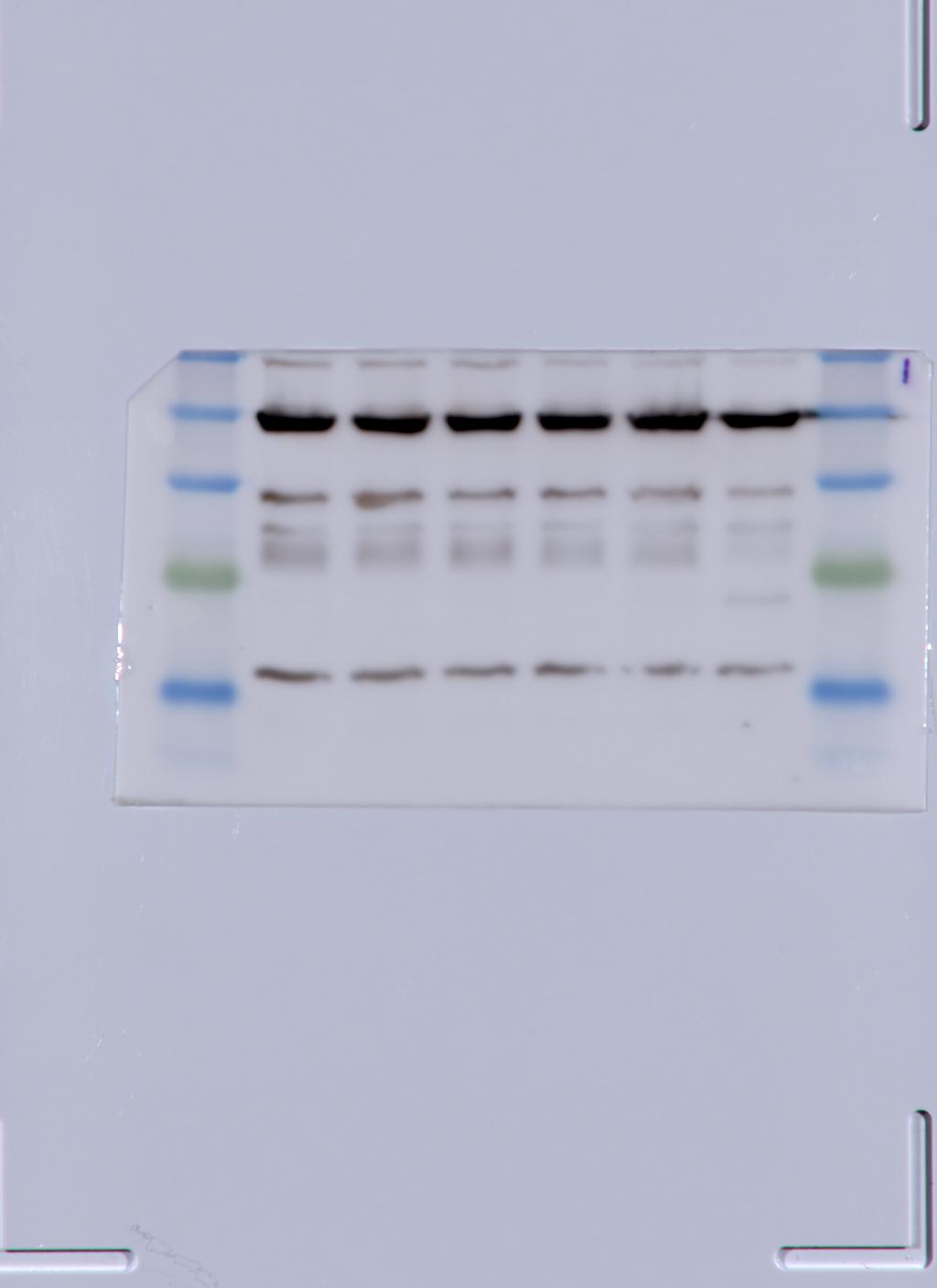

Supplement: Supplementary file 1 [file molecules-25-03667-s001.zip › molecules-891615 - proofread supplementary/Raw data of the Blots/Figure 3E/63-Fig 3E-b-Actin-1.jpg]

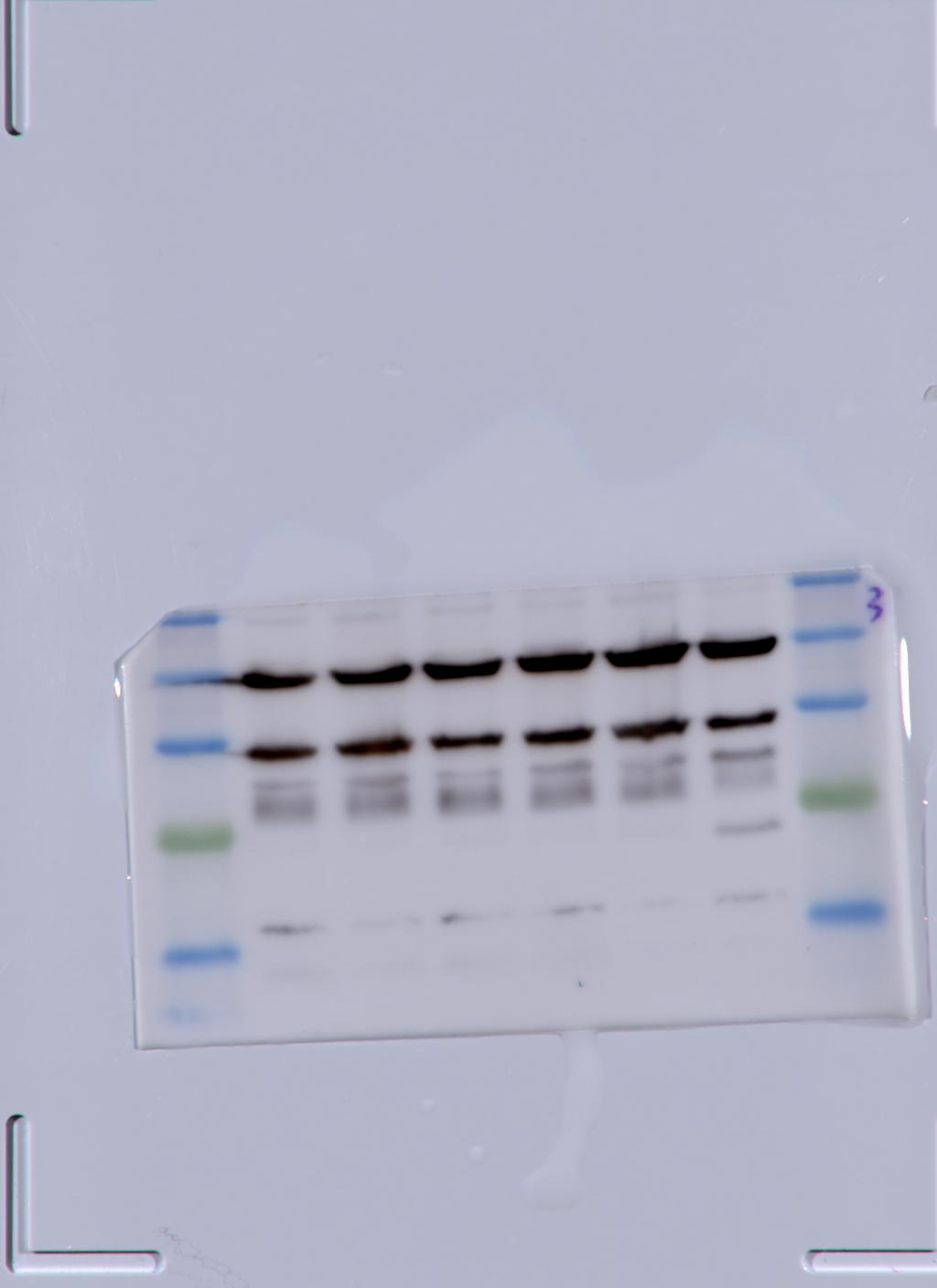

Supplement: Supplementary file 1 [file molecules-25-03667-s001.zip › molecules-891615 - proofread supplementary/Raw data of the Blots/Figure 3E/64-Fig 3E-b-Actin-2.jpg]

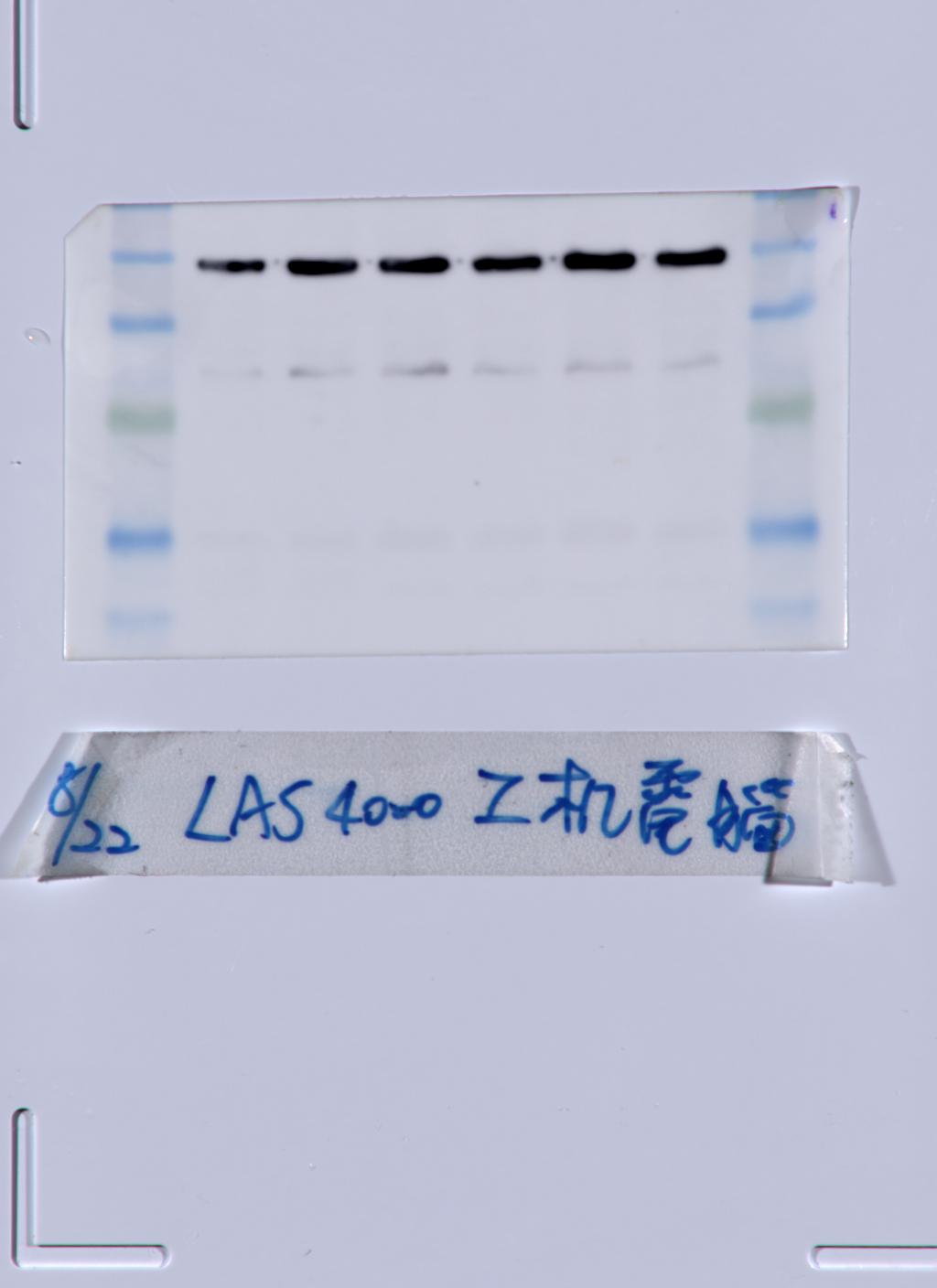

Supplement: Supplementary file 1 [file molecules-25-03667-s001.zip › molecules-891615 - proofread supplementary/Raw data of the Blots/Figure 3E/65-Fig 3E-b-Actin-3.jpg]

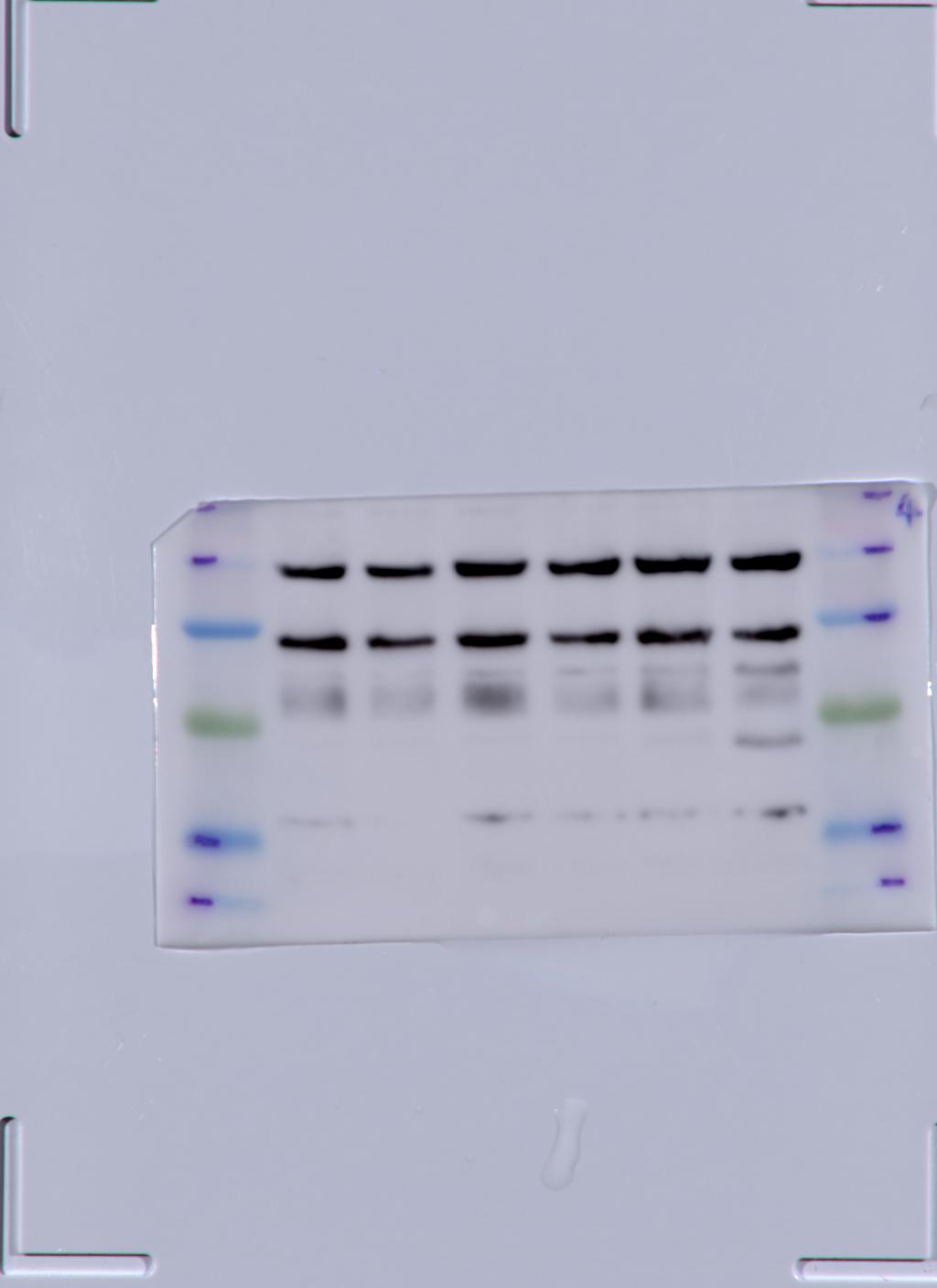

Supplement: Supplementary file 1 [file molecules-25-03667-s001.zip › molecules-891615 - proofread supplementary/Raw data of the Blots/Figure 3E/66-Fig 3E-b-Actin-4.jpg]

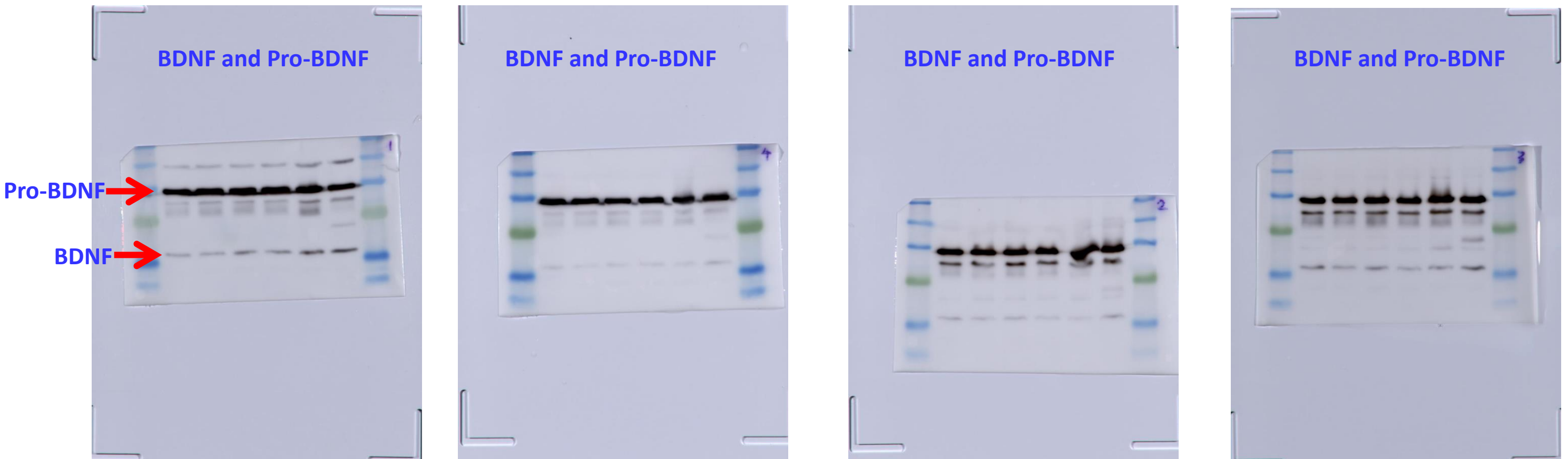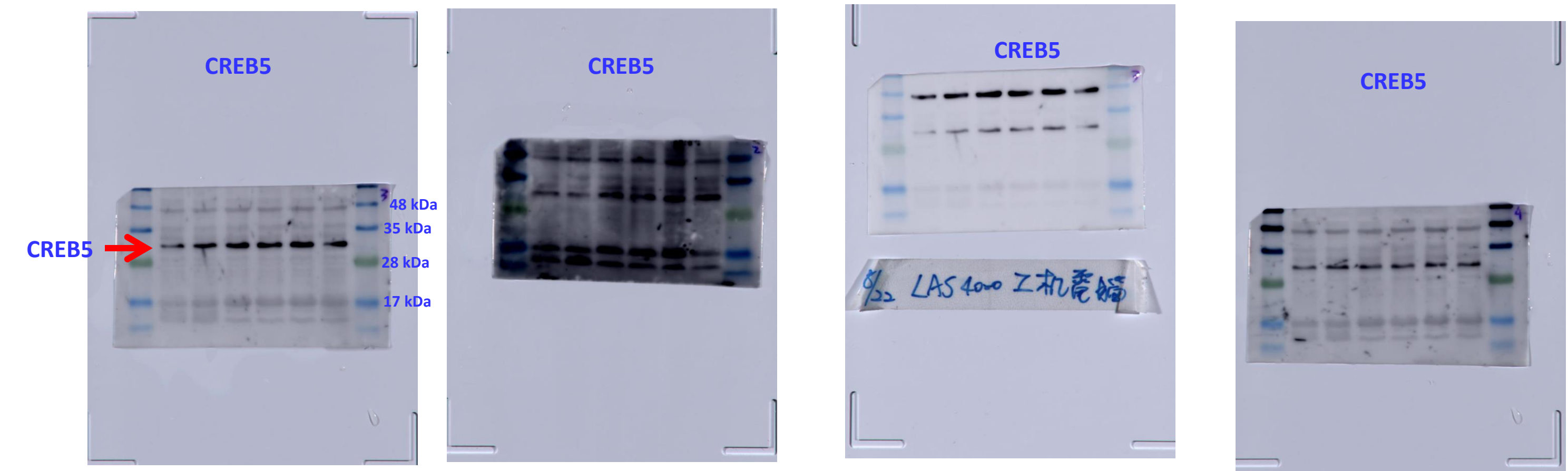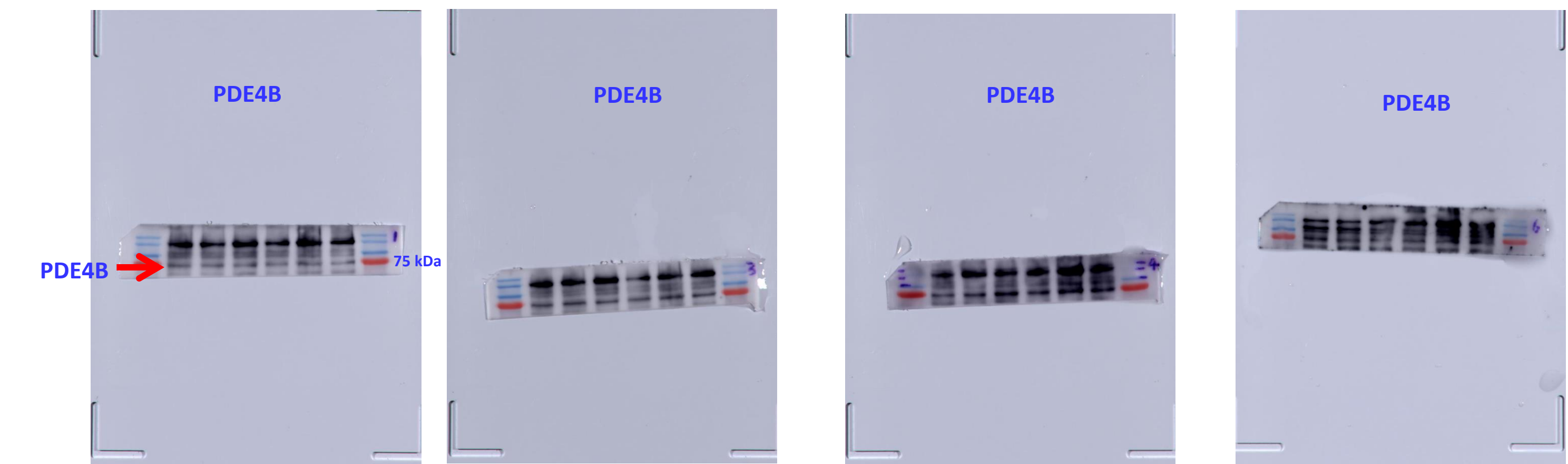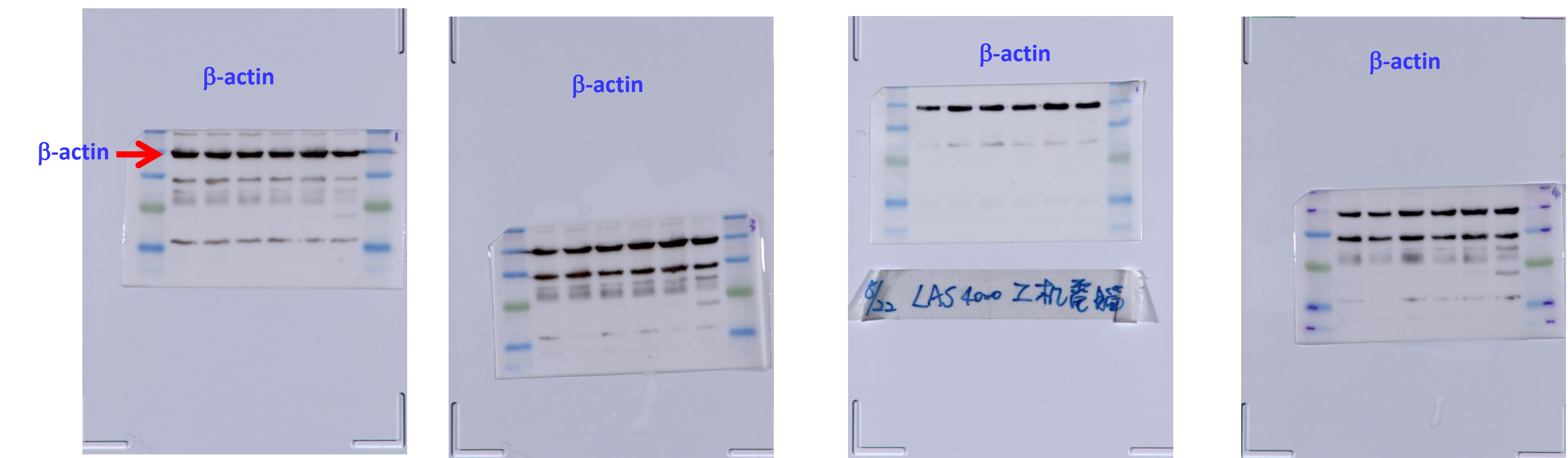

Supplement: Supplementary file 1 [file molecules-25-03667-s001.zip › molecules-891615 - proofread supplementary/Raw data of the Blots/Figure 3E/Fig 3E-Description for Figue 3E.pdf]

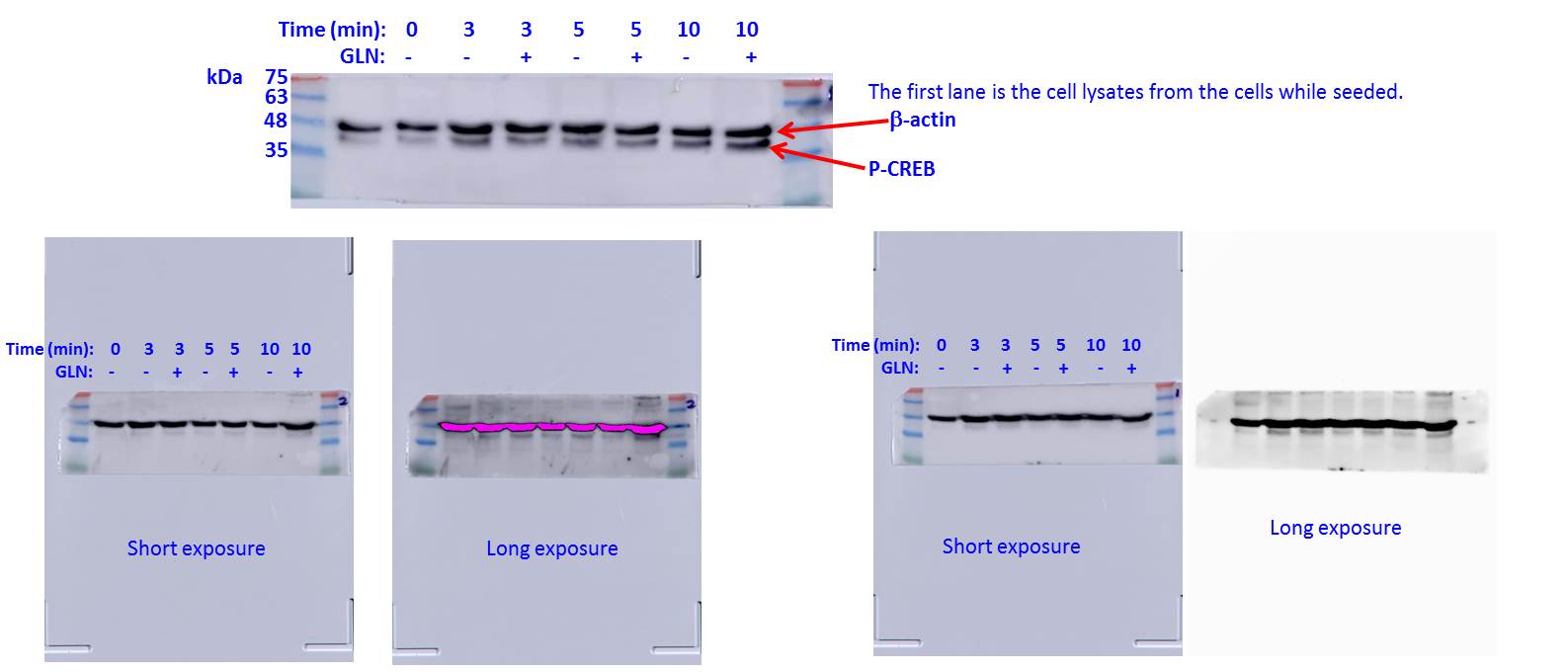

Supplement: Supplementary file 1 [file molecules-25-03667-s001.zip › molecules-891615 - proofread supplementary/Raw data of the Blots/Figure 4A/67-Fig 4A-Description for Figure 4A.jpg]

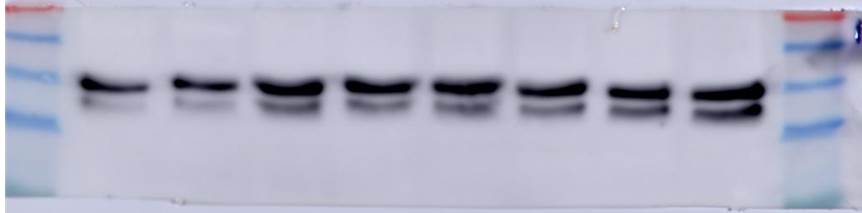

Supplement: Supplementary file 1 [file molecules-25-03667-s001.zip › molecules-891615 - proofread supplementary/Raw data of the Blots/Figure 4A/68-Fig 4A-Phospho-CREB and actin-1.jpg]

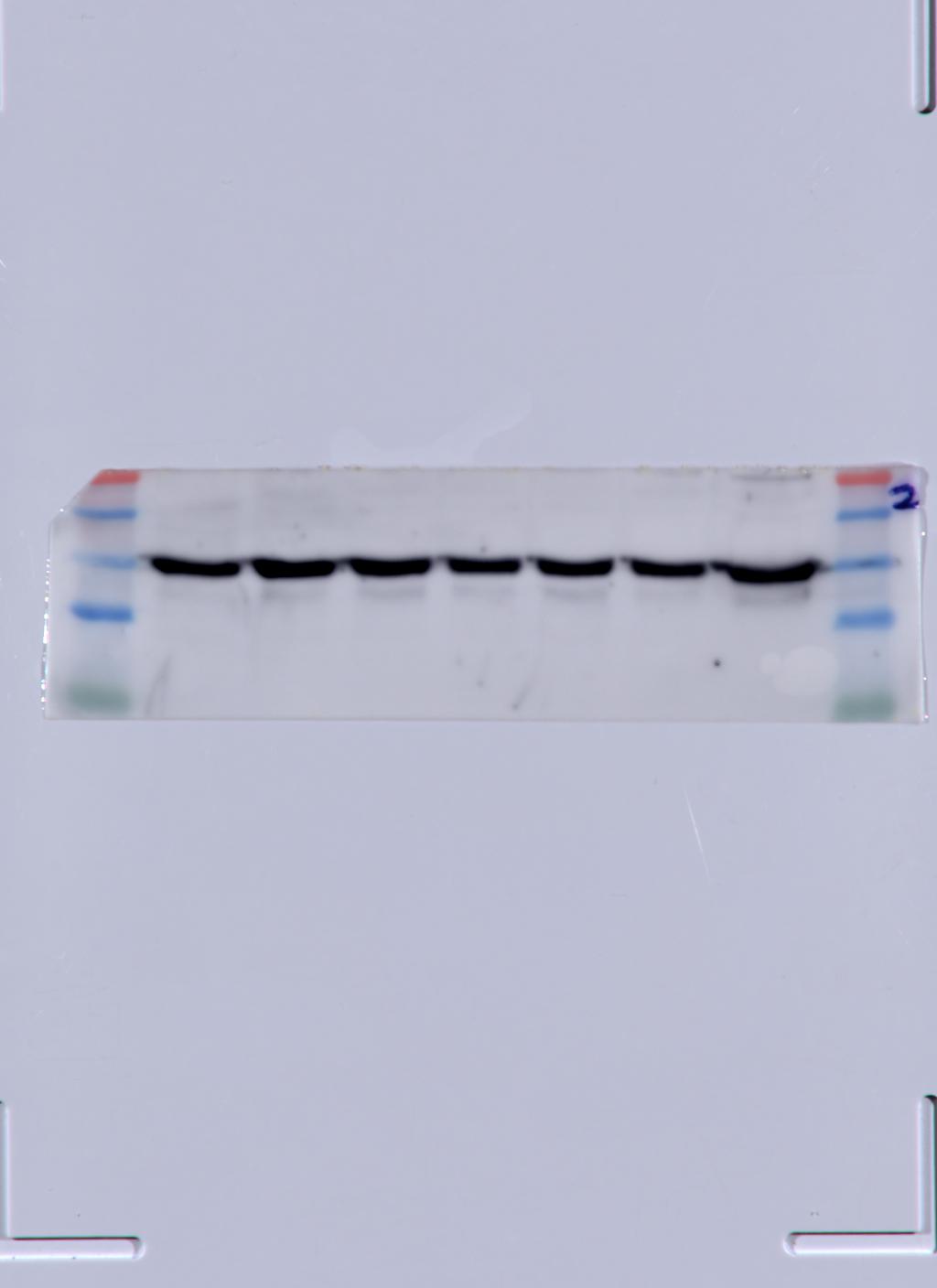

Supplement: Supplementary file 1 [file molecules-25-03667-s001.zip › molecules-891615 - proofread supplementary/Raw data of the Blots/Figure 4A/69-Fig 4A-phospho-CREB and actin-2-short.jpg]

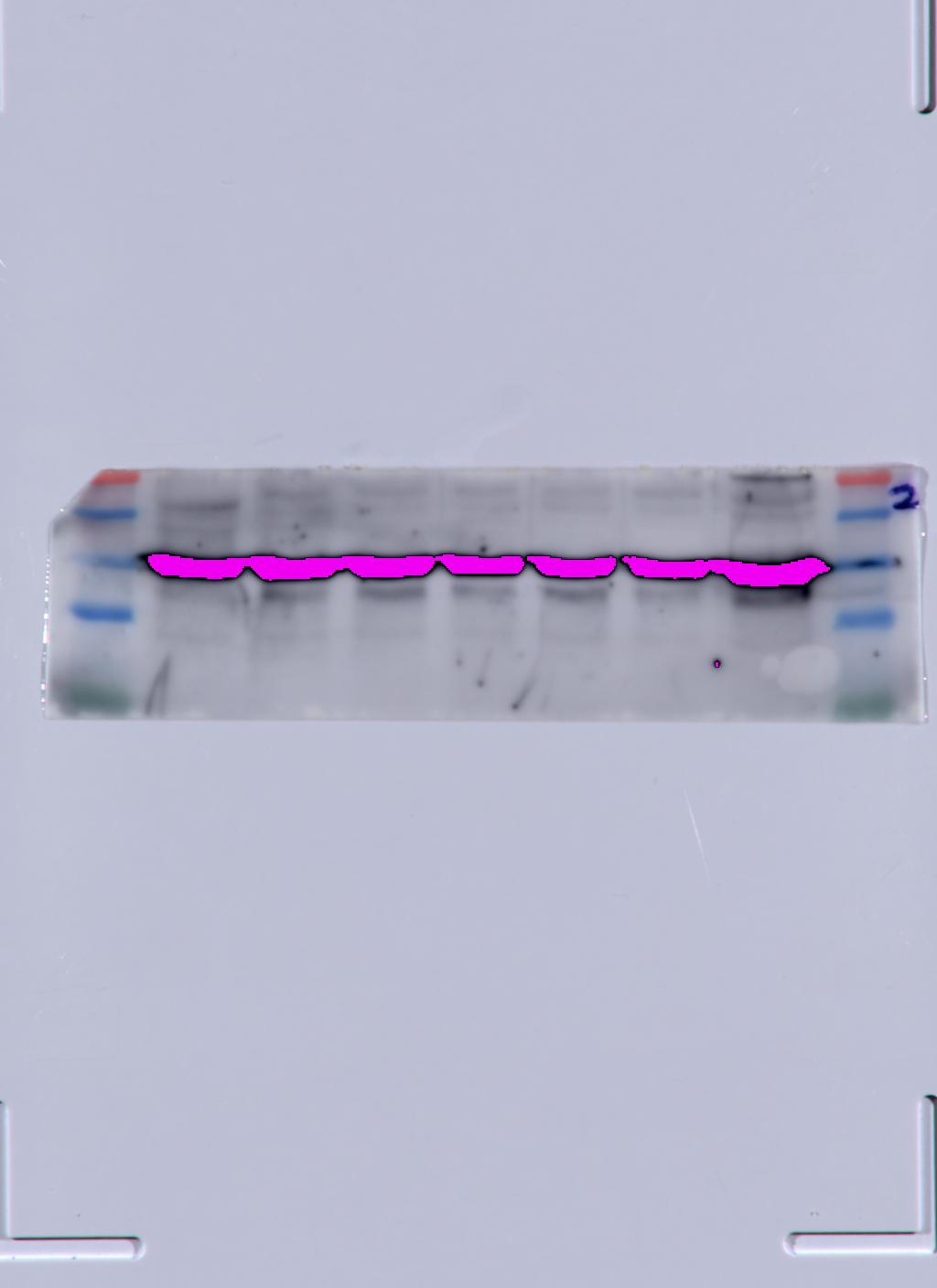

Supplement: Supplementary file 1 [file molecules-25-03667-s001.zip › molecules-891615 - proofread supplementary/Raw data of the Blots/Figure 4A/70-Fig 4A-phospho-CREB and actin-2-long.jpg]

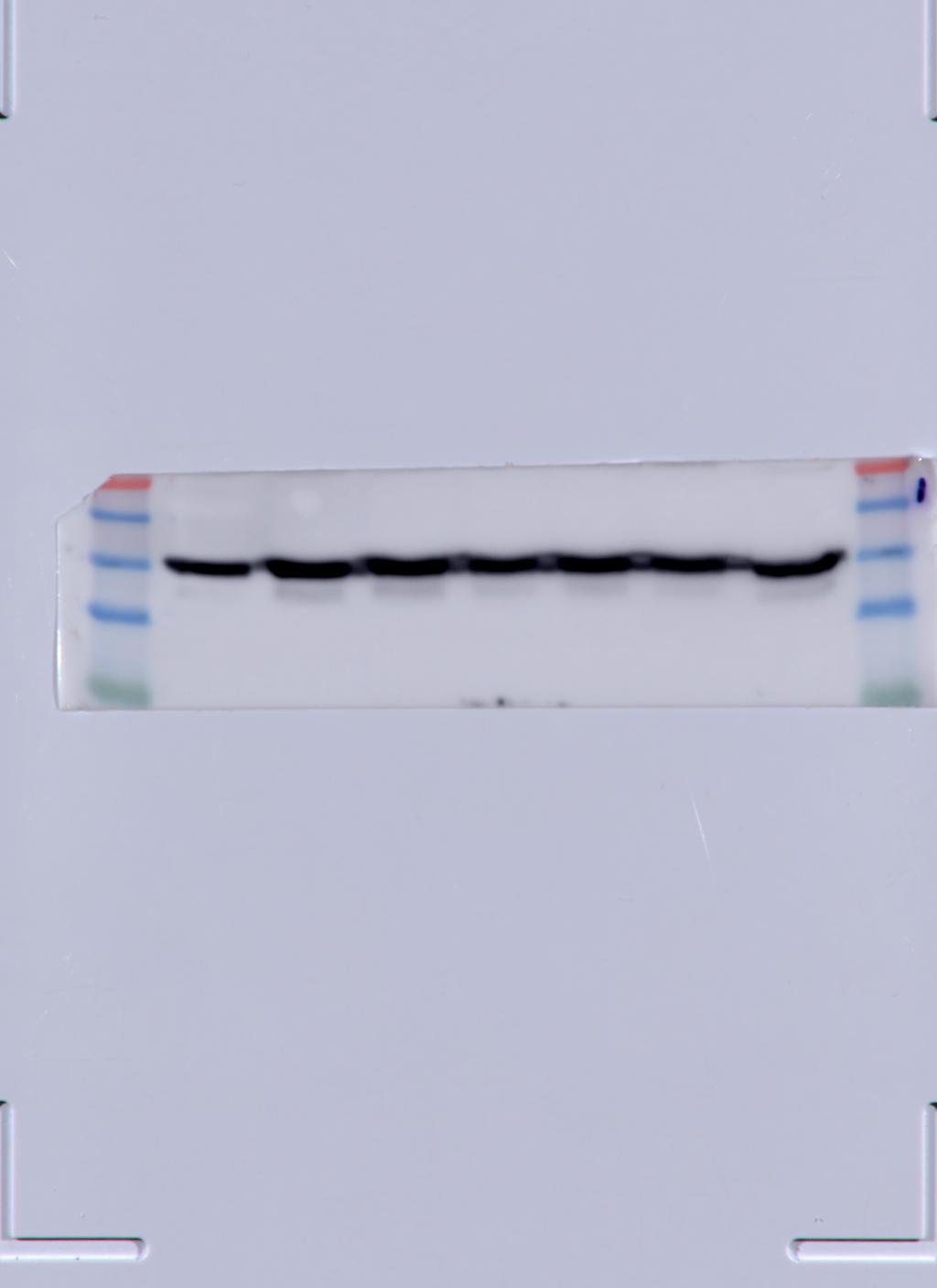

Supplement: Supplementary file 1 [file molecules-25-03667-s001.zip › molecules-891615 - proofread supplementary/Raw data of the Blots/Figure 4A/71-Fig 4A-phospho-CREB and actin-3-short.jpg]

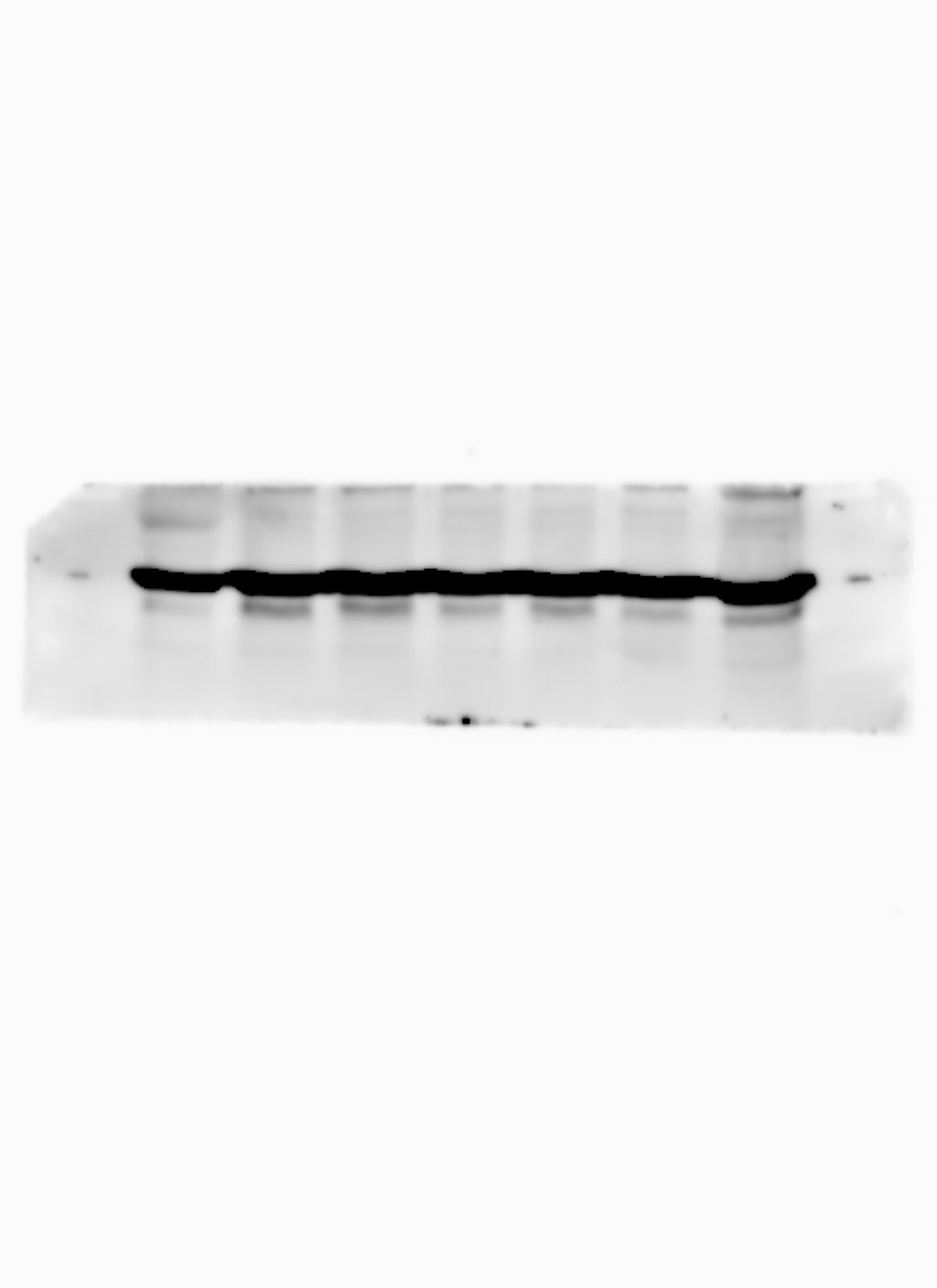

Supplement: Supplementary file 1 [file molecules-25-03667-s001.zip › molecules-891615 - proofread supplementary/Raw data of the Blots/Figure 4A/72-Fig 4A-phospho-CREB and actin-3-long.tif]

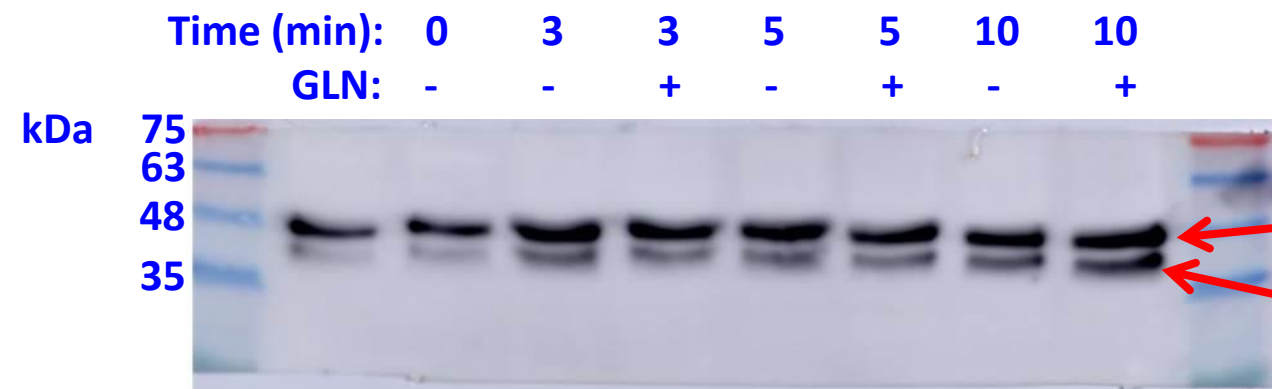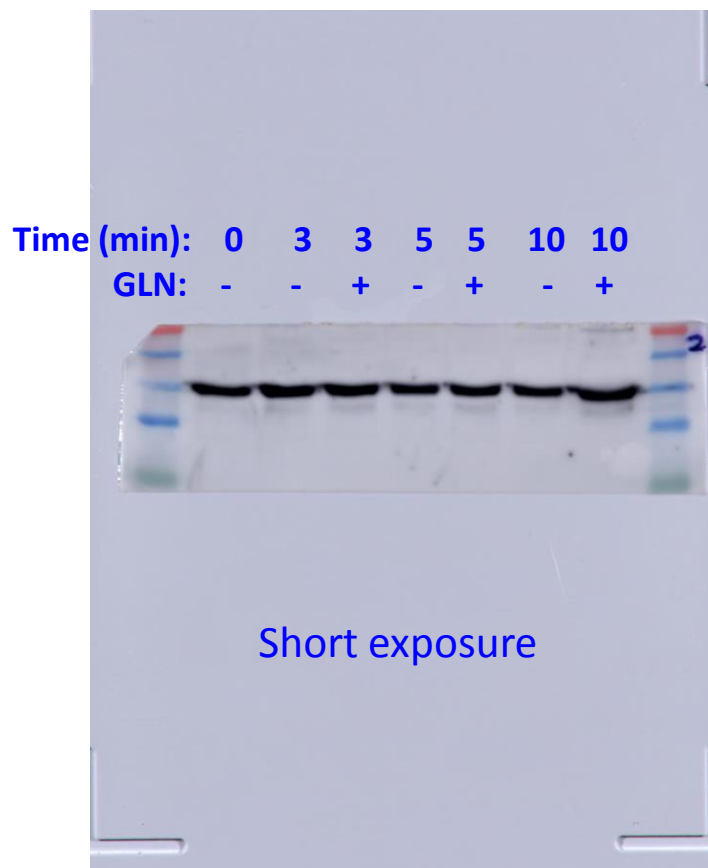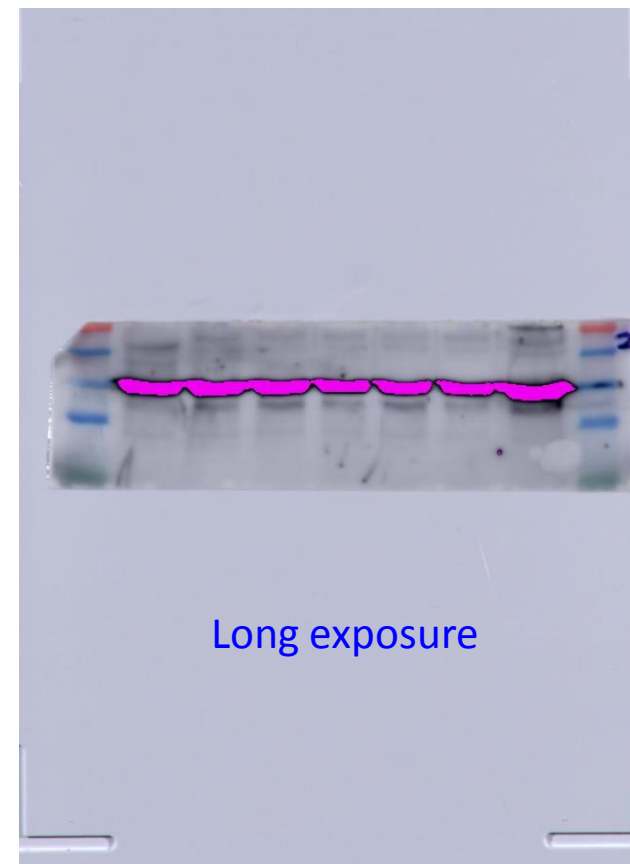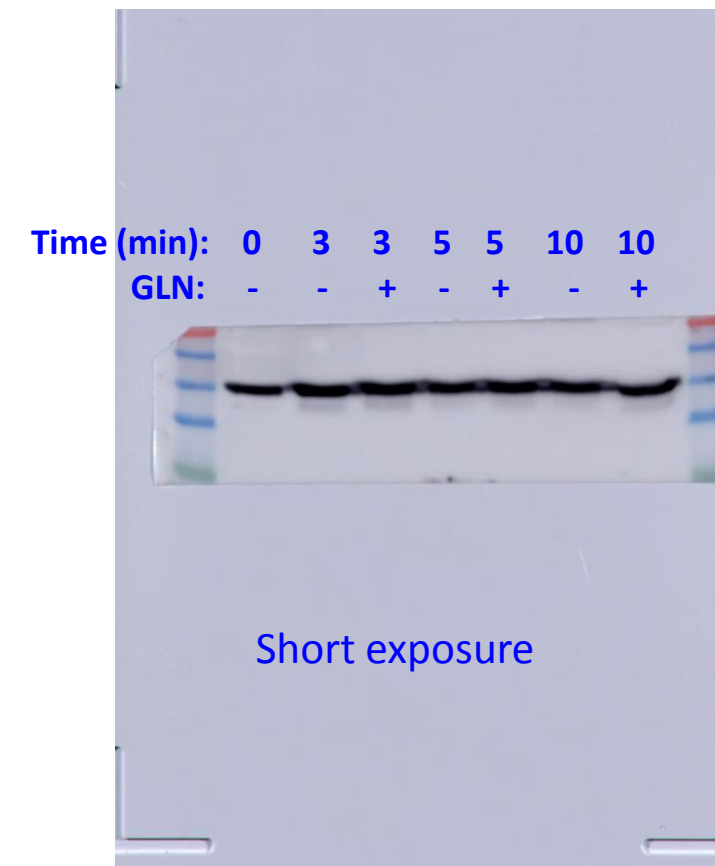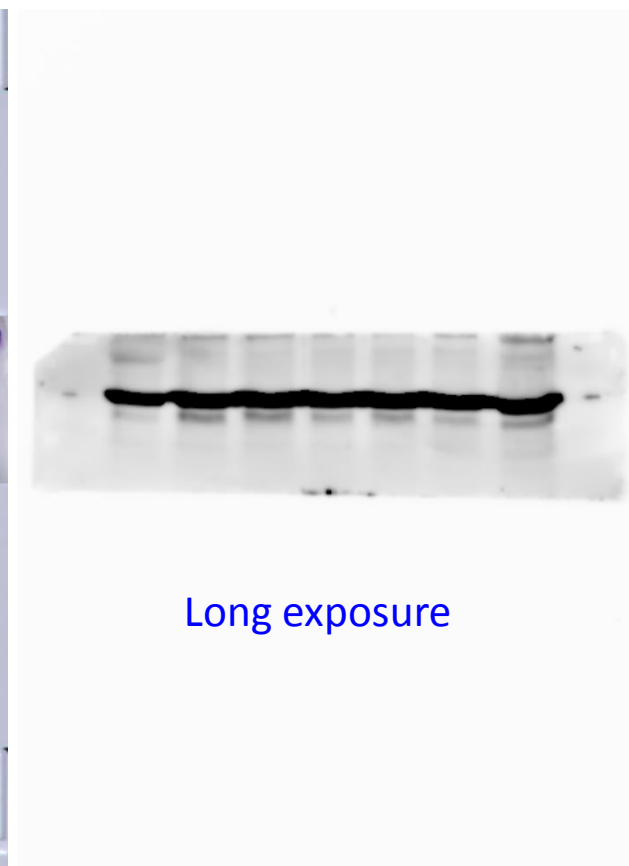

Supplement: Supplementary file 1 [file molecules-25-03667-s001.zip › molecules-891615 - proofread supplementary/Raw data of the Blots/Figure 4A/Fig 4A-Description for Figure 4A.pdf]

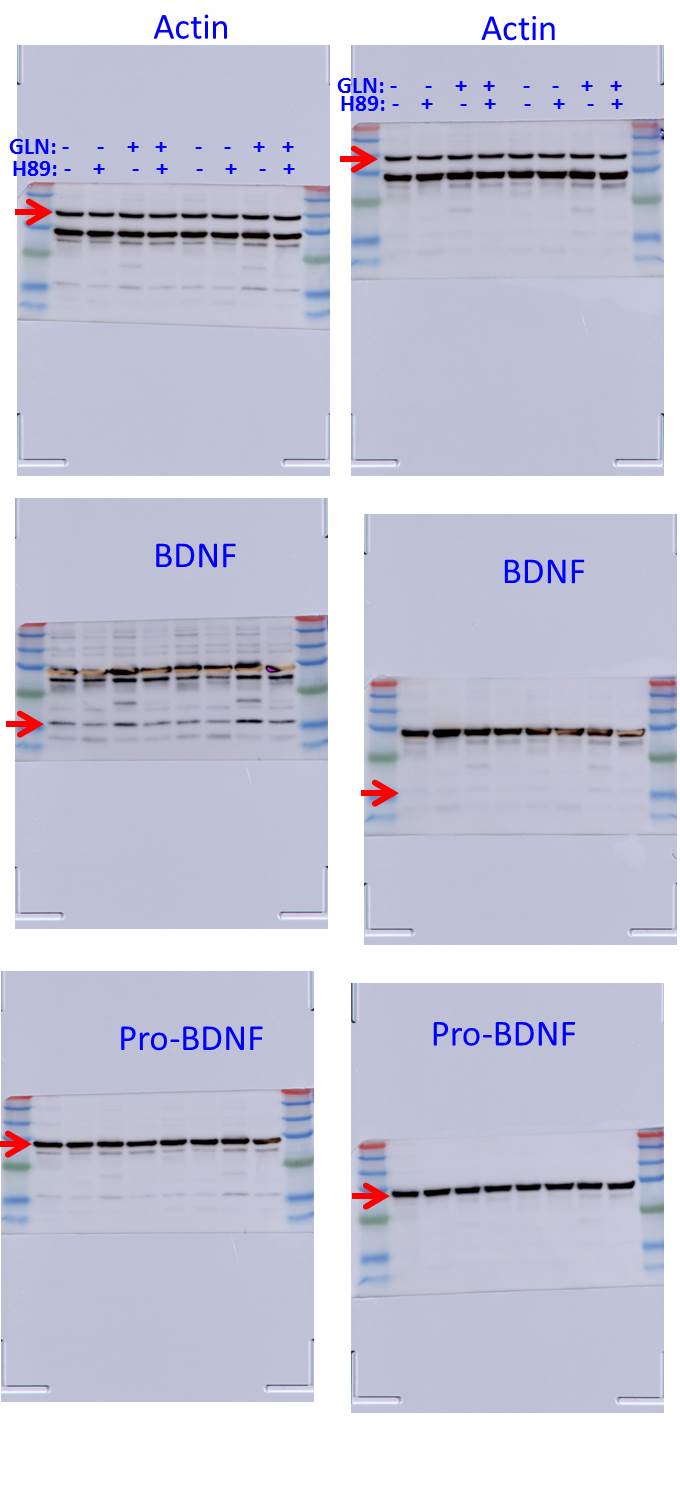

Supplement: Supplementary file 1 [file molecules-25-03667-s001.zip › molecules-891615 - proofread supplementary/Raw data of the Blots/Figure 4C/73-Fig 4C-Description for Figure 4C.jpg]

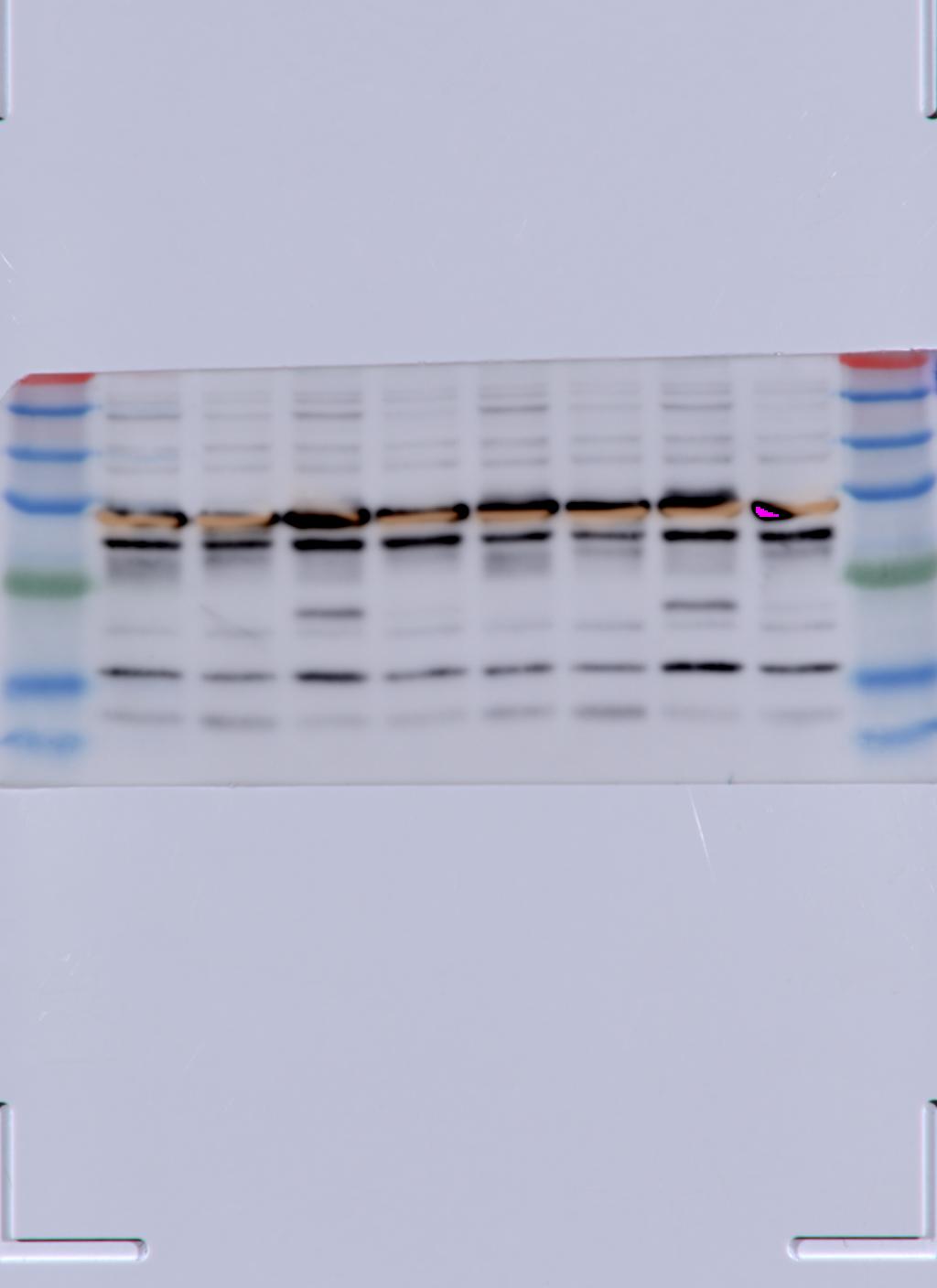

Supplement: Supplementary file 1 [file molecules-25-03667-s001.zip › molecules-891615 - proofread supplementary/Raw data of the Blots/Figure 4C/74-Fig 4C-BDNF-1 and 2.jpg]

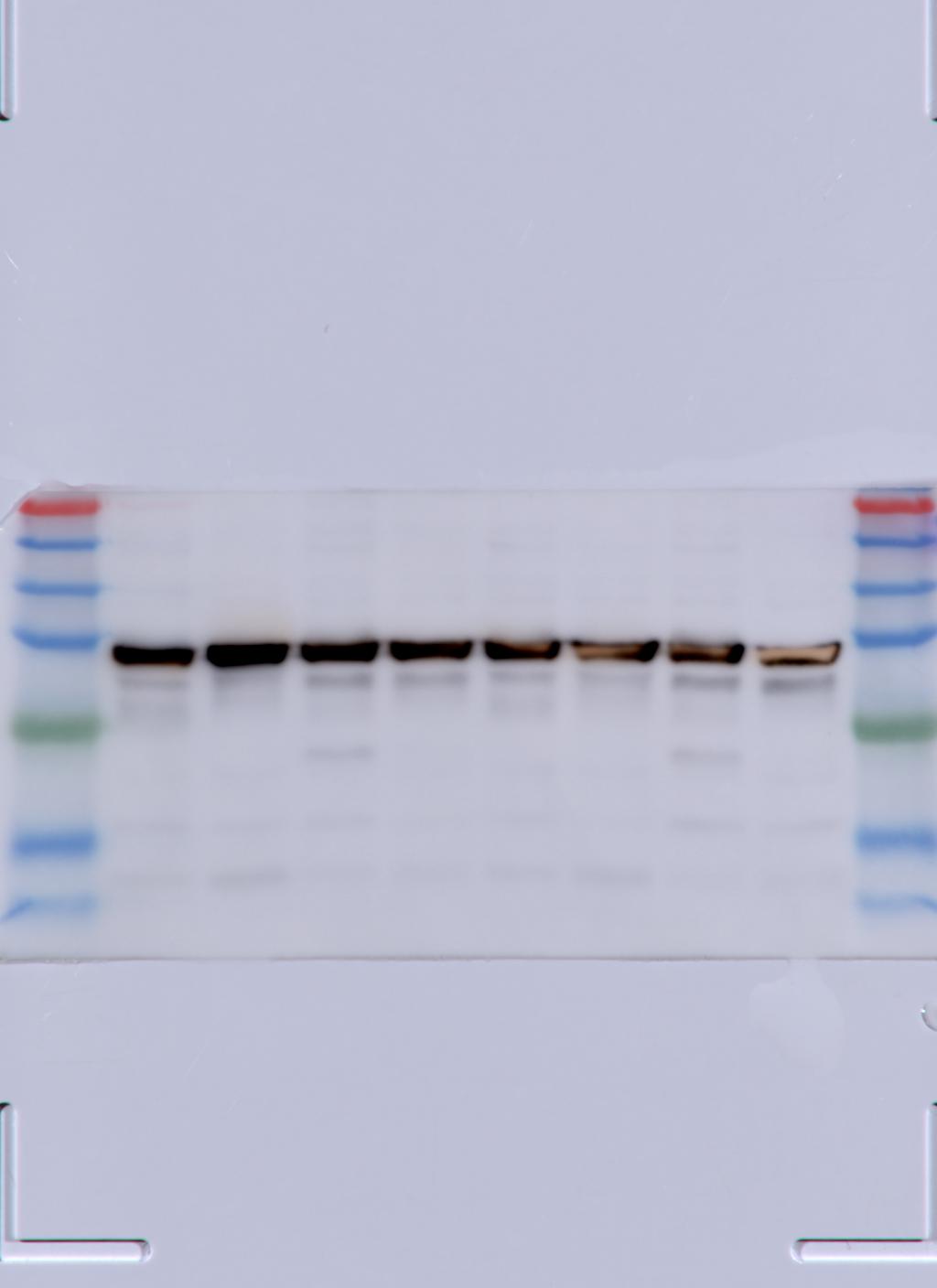

Supplement: Supplementary file 1 [file molecules-25-03667-s001.zip › molecules-891615 - proofread supplementary/Raw data of the Blots/Figure 4C/75-Fig 4C-BDNF-3 and 4.jpg]

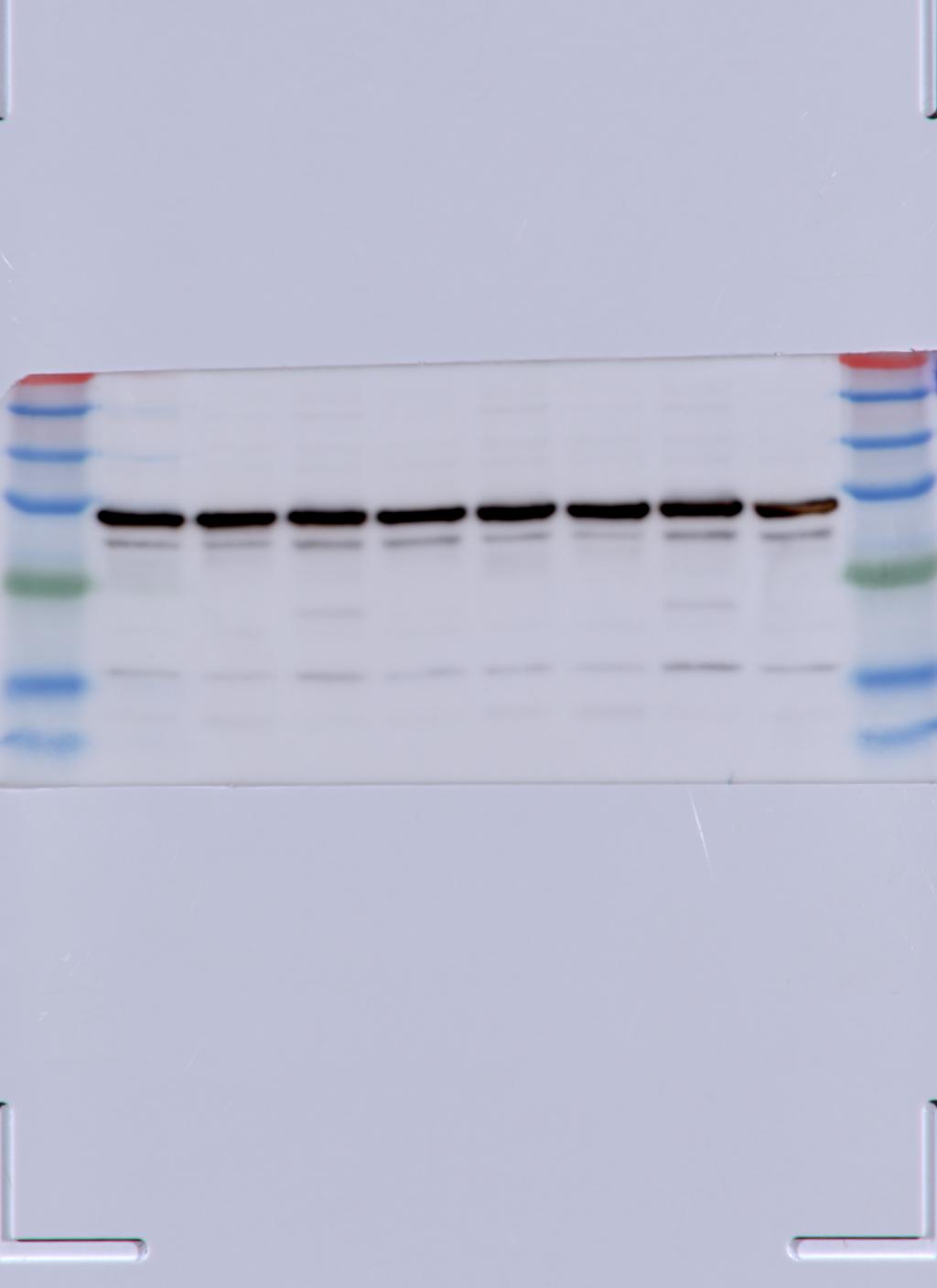

Supplement: Supplementary file 1 [file molecules-25-03667-s001.zip › molecules-891615 - proofread supplementary/Raw data of the Blots/Figure 4C/76-Fig 4C-Pro-BDNF-1 and 2.jpg]

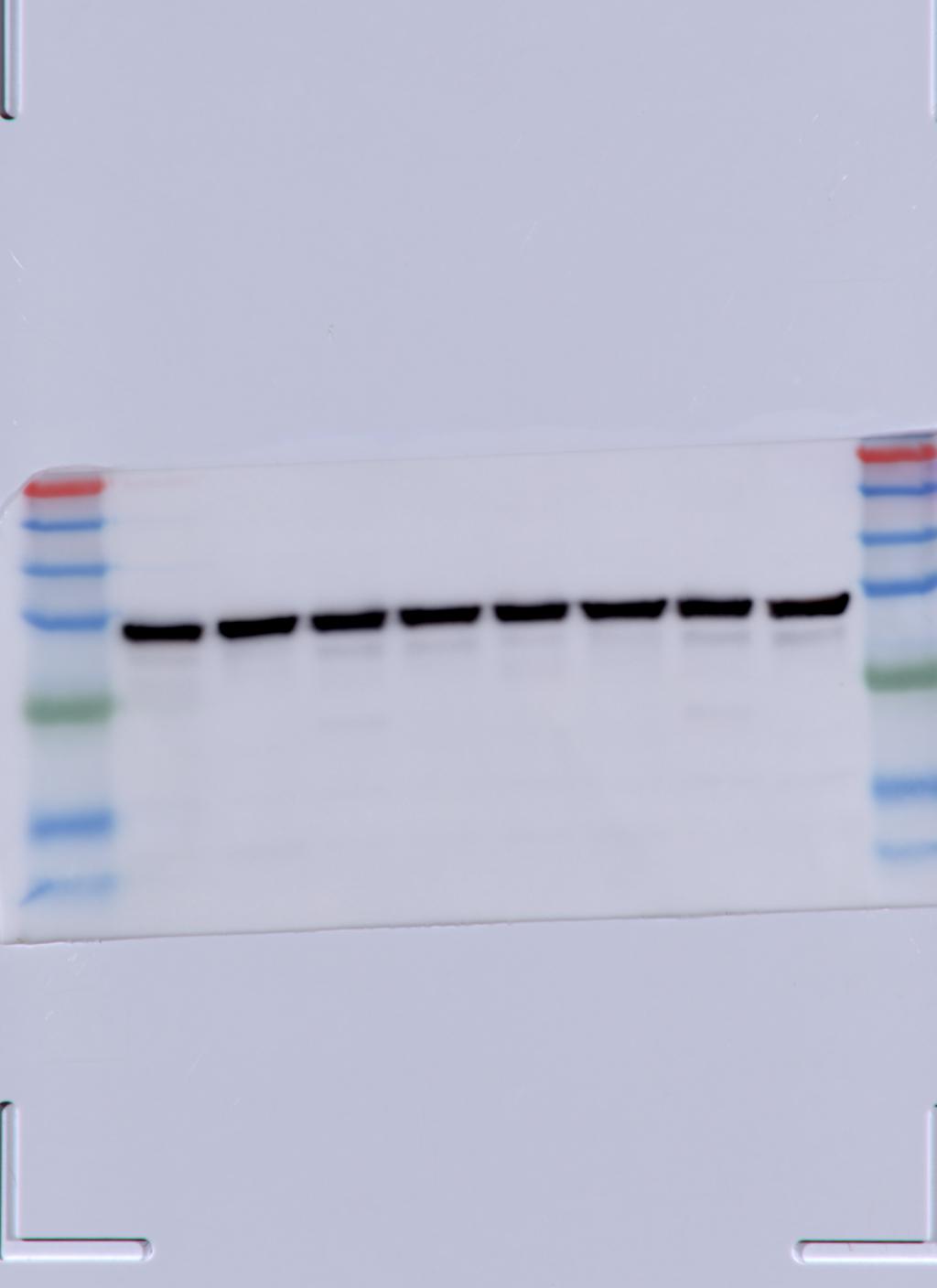

Supplement: Supplementary file 1 [file molecules-25-03667-s001.zip › molecules-891615 - proofread supplementary/Raw data of the Blots/Figure 4C/77-Fig 4C-Pro-BDNF-3 and 4.jpg]

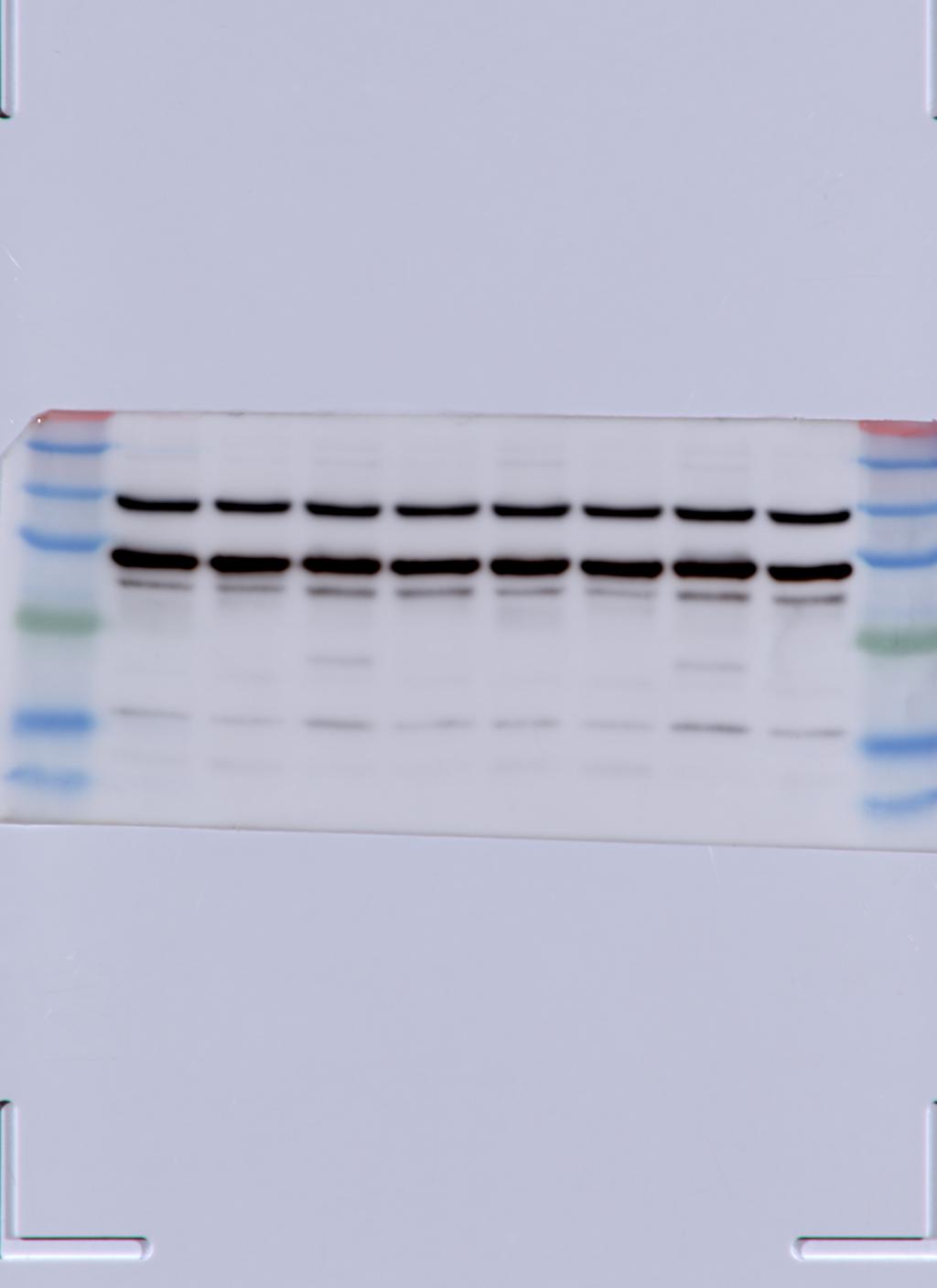

Supplement: Supplementary file 1 [file molecules-25-03667-s001.zip › molecules-891615 - proofread supplementary/Raw data of the Blots/Figure 4C/78-Fig 4C-Actin-1 and 2.jpg]

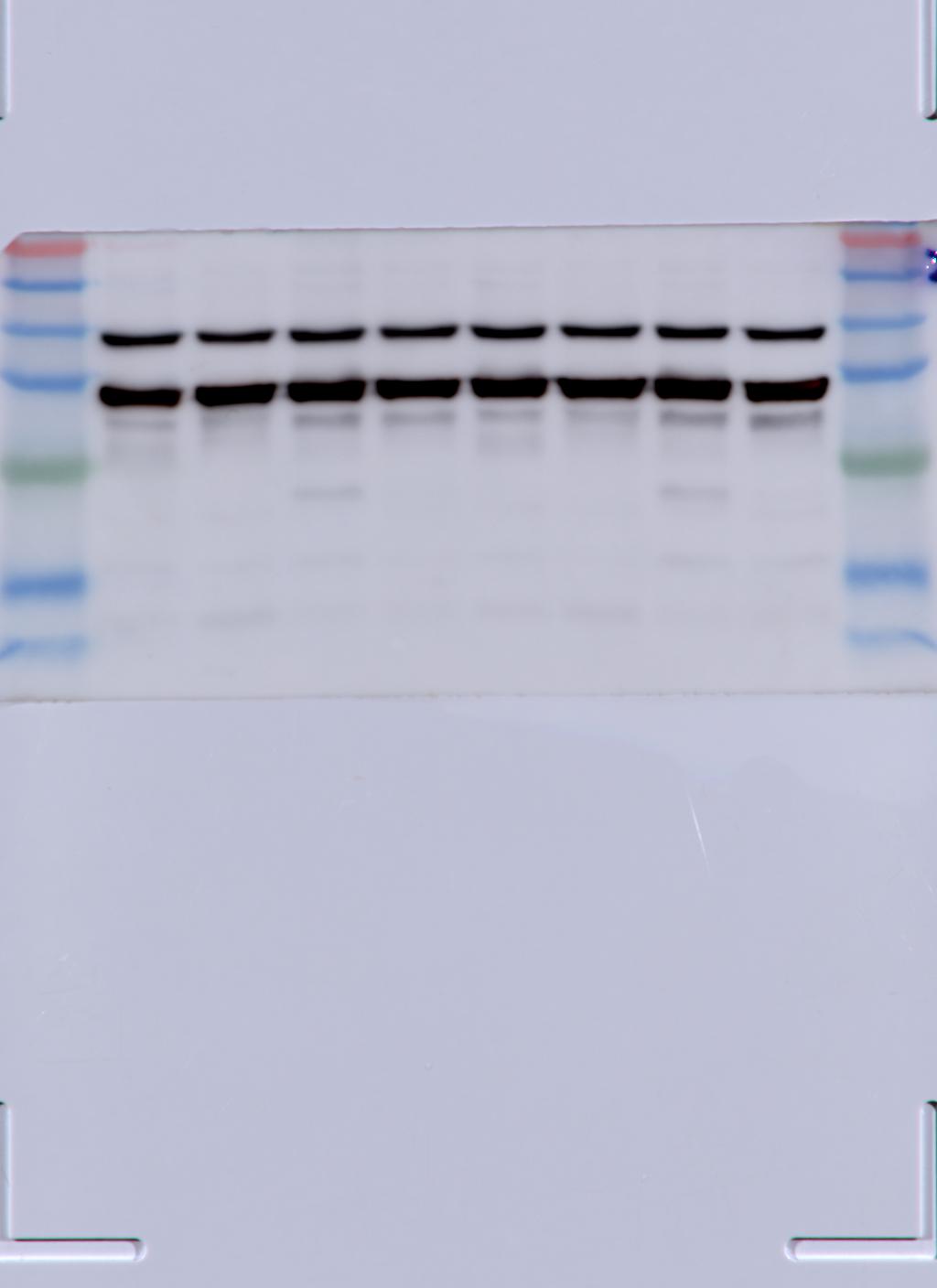

Supplement: Supplementary file 1 [file molecules-25-03667-s001.zip › molecules-891615 - proofread supplementary/Raw data of the Blots/Figure 4C/79-Fig 4C-Actin-3 and 4.jpg]

Actin

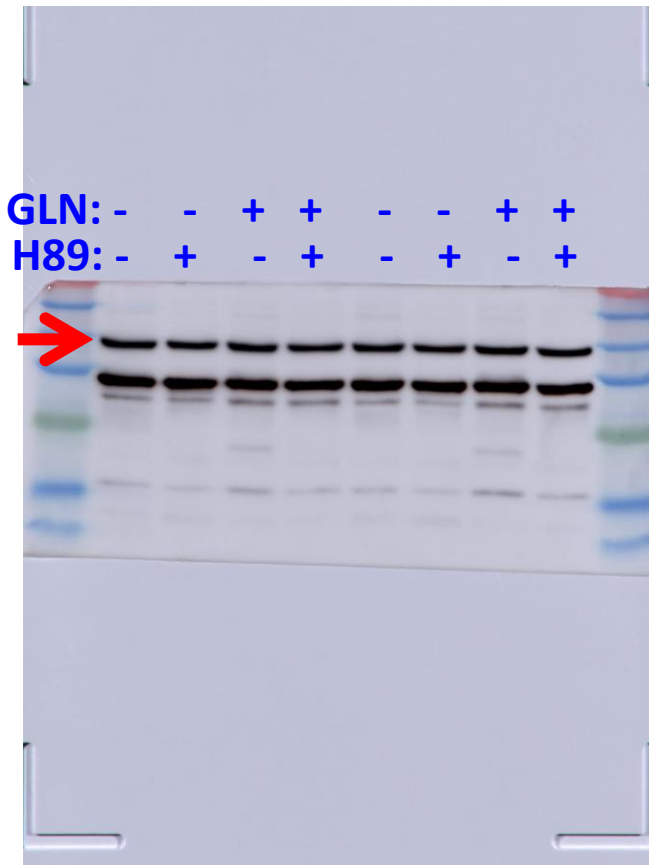

Actin

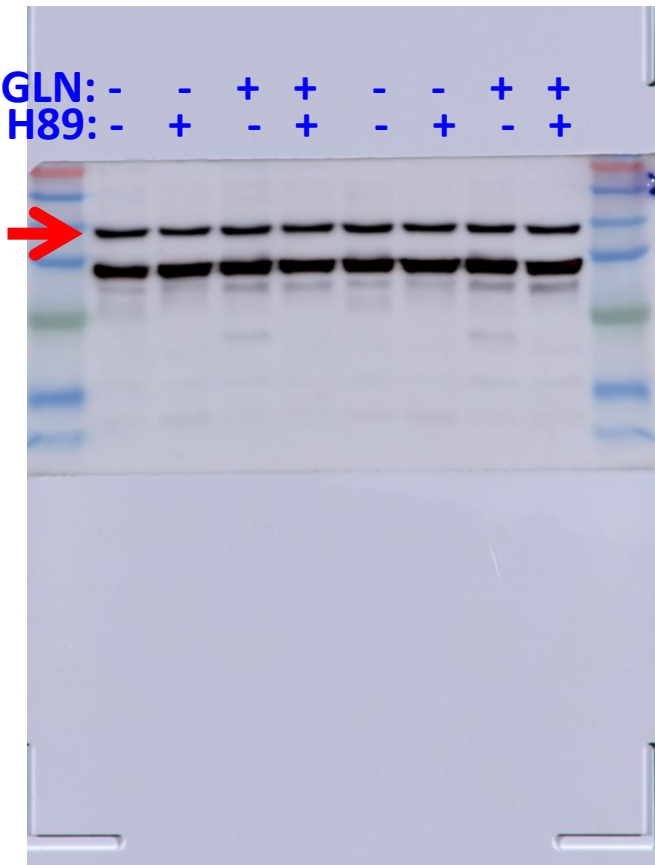

BDNF

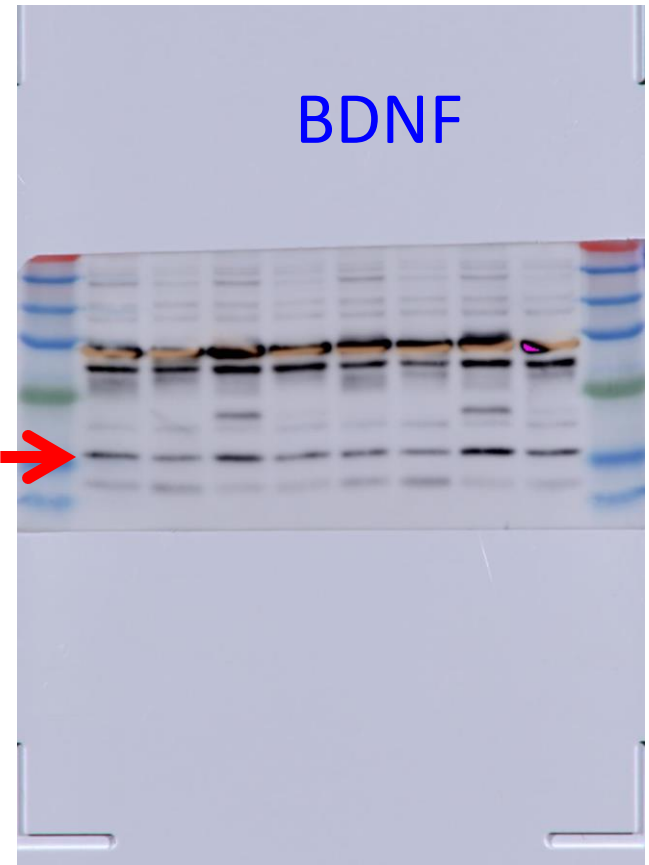

BDNF

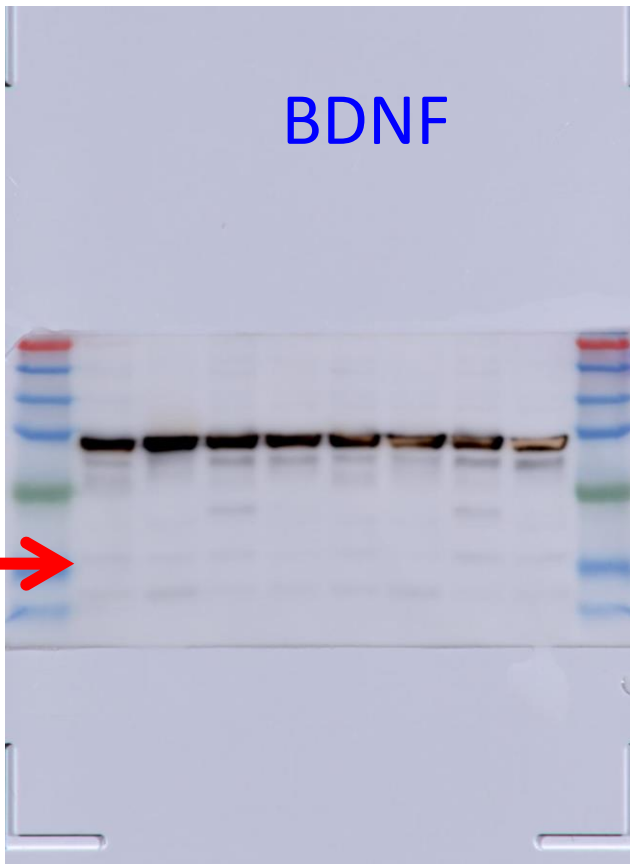

Pro-BDNF

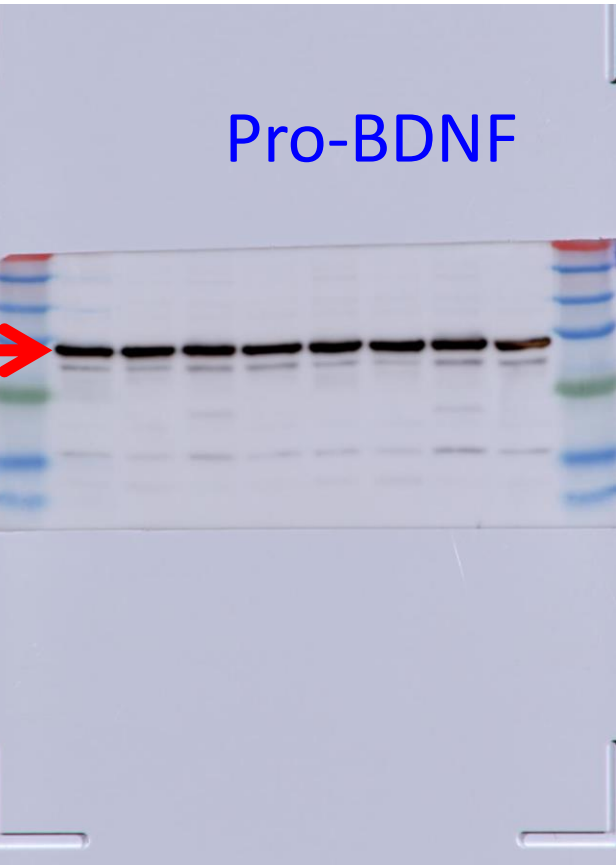

Pro-BDNF

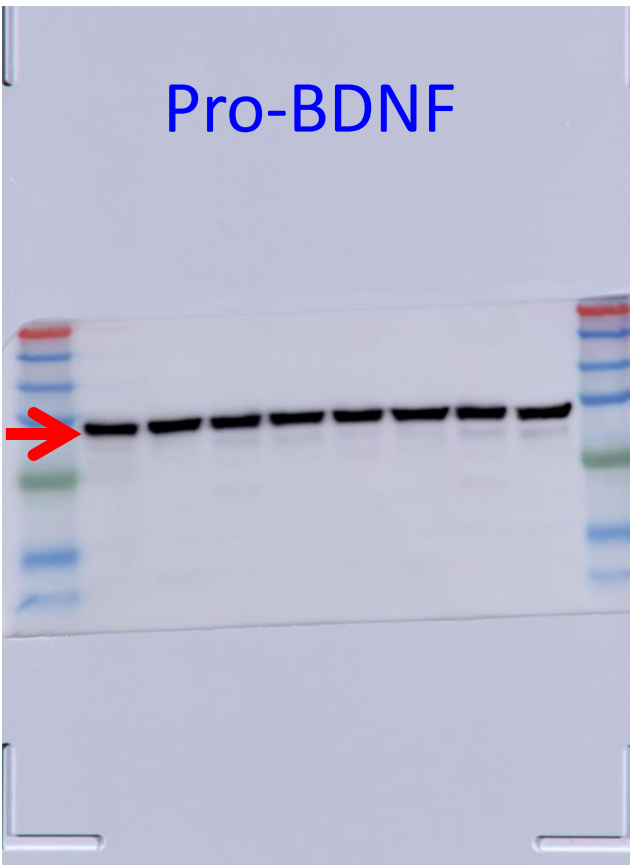

Supplement: Supplementary file 1 [file molecules-25-03667-s001.zip › molecules-891615 - proofread supplementary/Raw data of the Blots/Figure 4C/Fig 4C-Description for Figure 4C.pdf]

## Mice body weight

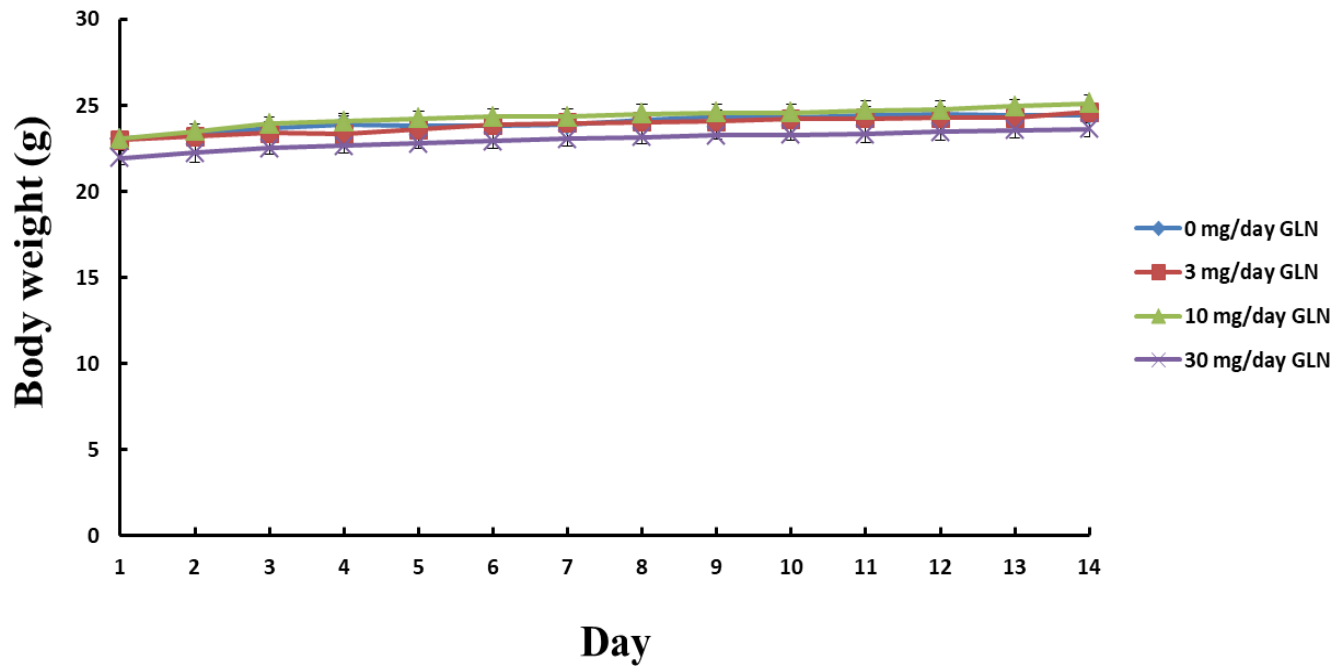

Supplement: Supplementary file 1 [file molecules-25-03667-s001.zip › molecules-891615 - proofread supplementary/Supplementary Figure 1.pdf]

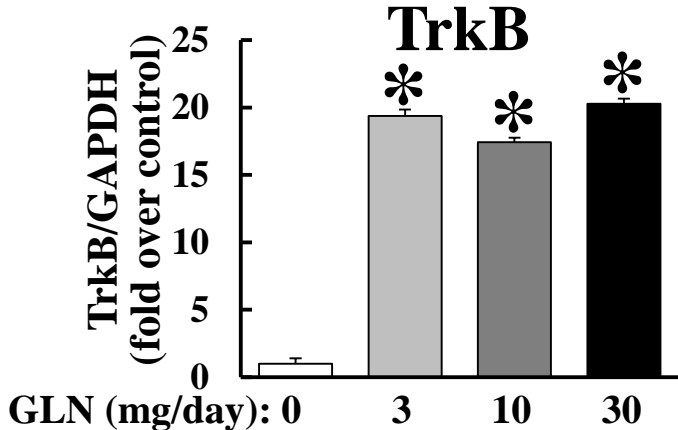

Supplement: Supplementary file 1 [file molecules-25-03667-s001.zip › molecules-891615 - proofread supplementary/Supplementary Figure 2.pdf]

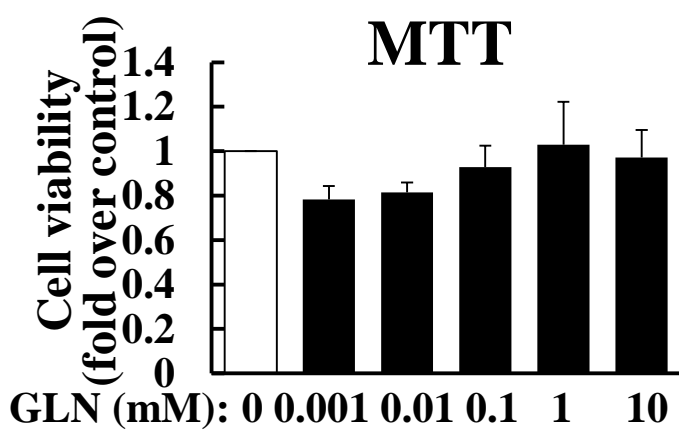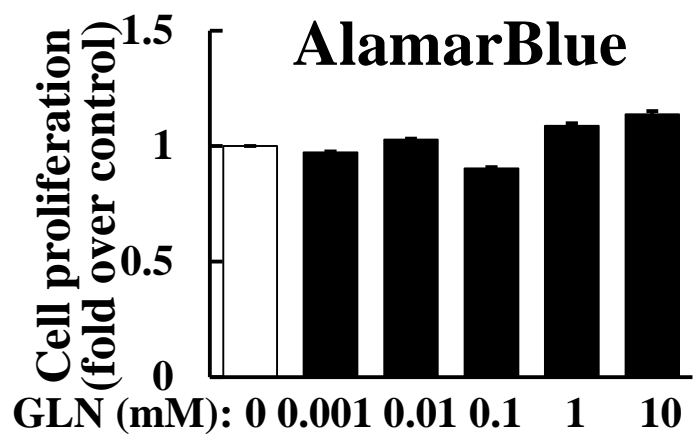

Supplement: Supplementary file 1 [file molecules-25-03667-s001.zip › molecules-891615 - proofread supplementary/Supplementary Figure 3.pdf]
